# Black phosphorus-loaded mineralized UV-responsive chitosan hydrogel for enhanced osteogenesis and antibacterial activity

**DOI:** 10.1093/rb/rbag128

**Published:** 2026-06-17

**Authors:** Xiaoying Xu, Xiaofeng Yang, Tiantian Gao, Jinjin Zhu, Chao He, Tuwei Jin, Jiuzhou Dong, In-Seop Lee, Cen Chen, Fan Liu

**Affiliations:** College of Life Sciences and Medicine, Zhejiang Sci-Tech University, Hangzhou 310018, China; Department of Orthodontics, School of Stomatology, China Medical University, Shenyang 110002, China; College of Life Sciences and Medicine, Zhejiang Sci-Tech University, Hangzhou 310018, China; Department of Orthopaedic Surgery, Sir Run Shaw Hospital, Zhejiang University School of Medicine & Zhejiang Key Laboratory of Mechanism Research and Precision Repair of Orthopedic Trauma and Aging Diseases, Hangzhou 310018, China; Department of Orthodontics, School of Stomatology, China Medical University, Shenyang 110002, China; College of Life Sciences and Medicine, Zhejiang Sci-Tech University, Hangzhou 310018, China; Department of Orthodontics, School of Stomatology, China Medical University, Shenyang 110002, China; Institute of Human Materials, Suwon 16514, Republic of Korea; College of Life Sciences and Medicine, Zhejiang Sci-Tech University, Hangzhou 310018, China; Department of Orthodontics, School of Stomatology, China Medical University, Shenyang 110002, China

**Keywords:** chitosan, mineralization, osteogenesis, black phosphorus nanosheets, antibacterial, NIR irradiation

## Abstract

Favorable osteogenesis and effective antibacterial activity are two essential requirements for ideal scaffolds in bone tissue engineering, particularly for infected bone defects. Herein, we developed a novel hydrogel system based on mineralized ultraviolet-responsive methacrylated chitosan hydrogel (CSMA) integrated with black phosphorus (BP) nanosheets exhibiting a photothermal effect. BP was incorporated into CSMA and subsequently subjected to *in situ* biomineralization to form a BP-loaded mineralized hydrogel (BP@CSMA/CaCO_3_). The physicochemical and biological properties of BP@CSMA/CaCO_3_ were systematically investigated *in vitro*. BP incorporation and CaCO_3_ mineralization improved hydrogel stability and enabled sustained Ca^2+^ and phosphorus ion release, thereby supporting an osteogenesis-favorable microenvironment. Under high-intensity near-infrared (NIR) irradiation, BP-containing hydrogels exhibited strong antibacterial activity, which was supported by both photothermal heating and singlet oxygen generation. Under mild NIR stimulation, BP@CSMA/CaCO_3_ promoted bone mesenchymal stem cells (BMSCs) proliferation and osteogenic differentiation, with increased ALP activity and osteogenic gene expression. Transcriptomic analysis further suggested the involvement of cytoskeletal and immune-related regulation in the enhanced osteogenic response. The application of BP@CSMA/CaCO_3_ in a calvarial defect model *(in vivo*) indicated its superior bone formation ability. These findings suggested that BP@CSMA/CaCO_3_ integrated antibacterial activity, ion-mediated osteogenic support and NIR-responsive regulation had great potential for bone regeneration.

## Graphical abstract

**Graphical Abstract:**
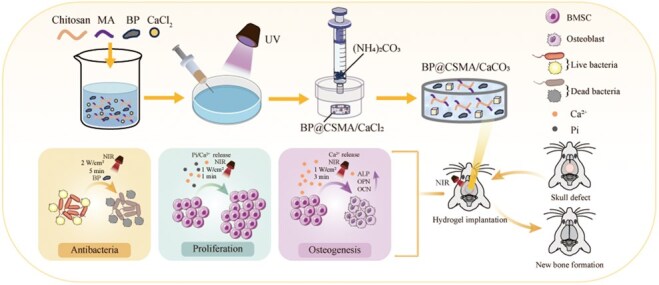

## Introduction

With population aging and increasing prevalence of unhealthy lifestyles, over 200 million people worldwide are affected by skeletal system disorders, including osteoarthritis, osteoporosis, osteonecrosis or fracture, and this number continues to rise at an annual rate of 10% [1]. Clinically, bone defects require surgical intervention using bone graft materials, such as autografts, allografts, xenografts, etc. [2]. It is estimated that the global market shares of bone substitute materials will reach USD 56 billion by 2034. Given the limited source of autografts, the development and application of artificial bone substitutes have become increasingly crucial in orthopedic therapy [3].

Hydrogels are widely utilized in biomedical applications owing to their 3D network structure, which facilitates nutrient transport and metabolic waste exchange [4]. In bone tissue engineering, hydrogels generally demonstrate good biocompatibility but often lack osteoconductive and osteoinductive capabilities [5]. Chitosan (CS), a natural polysaccharide derived from the deacetylation of chitin, has been extensively investigated for bone regeneration due to its low toxicity, excellent biocompatibility and biodegradability [6–9]. Methacrylated chitosan (CSMA) is synthesized through the reaction of amino groups in CS with methacrylic anhydride (MA), introducing photo-crosslinkable moieties that enable a sol-to-gel transition under ultraviolet (UV) irradiation [10]. This property allows CSMA to be injected and gelled *in situ*, adapting to bone defects of various geometries [11]. Despite these advantages, the limited intrinsic osteogenic properties of CSMA still restrict its broader application in bone regeneration [12]. To overcome this limitation, we previously incorporated calcium carbonate (CaCO_3_) particles into CSMA via *in situ* mineralization [13]. The resulting mineralized hydrogel enabled sustained release of Ca^2+^, which effectively enhanced the osteogenic differentiation of bone mesenchymal stem cells (BMSCs) [14].

However, the promotion of osteogenesis alone is insufficient, as implant-related infection, occurring in 0.7–4.2% of orthopedic surgeries, remains another major obstacle to successful bone repair [15]. A competitive relationship exists between the bacterial adhesion and host cell attachment on the surface of bone substitutes after orthopedic surgery, particularly during the critical early period (4–6 h) after implantation [16, 17]. Bacterial colonization is determined by the initial bacterial load, while the adhesion of cells with prominent osteogenic capability is essential for new bone formation, indicating that the simultaneous or sequential antibacterial and osteogenic effects are necessary for bone substitutes [18–21]. Consequently, there is an urgent need to develop modification strategies that impart multifunctional bioactivity to implanted hydrogels.

These challenges highlight the need for biomaterials that can provide antibacterial protection while supporting subsequent bone regeneration [22]. Black phosphorus (BP) nanosheets are representative 2D photothermal nanomaterials composed of layered phosphorus atoms [23]. Their layer-dependent bandgap and broad near-infrared (NIR) absorption endow BP with efficient photothermal and photodynamic properties [24]. BP has attracted growing interest in biomedical applications owing to its non-invasiveness, remote controllability, biodegradability and biocompatibility [25]. BP can exert different biological effects by adjusting NIR irradiation conditions. Specifically, high-intensity NIR irradiation induces antibacterial activity through photothermal heating and reactive oxygen species (ROS) generation, whereas mild thermal stimulation can promote osteogenesis [26–28]. Furthermore, the released phosphorus ions during BP degradation are essential for new bone formation [29]. Based on these considerations, BP was introduced into the mineralized CSMA system to construct a multifunctional hydrogel with both photothermal antibacterial activity and osteogenic potential [30, 31]. However, its inherent instability remains a major obstacle for future clinical translation [32].

To address the limited osteogenic and antibacterial capabilities of CSMA hydrogels [33], we designed a novel mineralized UV-responsive hydrogel by integrating BP nanosheets with an *in situ*-mineralized CSMA matrix (BP@CSMA/CaCO_3_). The selection of BP and CSMA mineralization was based on their complementary functional properties. BP was introduced as the NIR-responsive component due to its photothermal behaviors, which enabled switchable antibacterial and osteogenic effects, and phosphorus release pattern during degradation [34]. Meanwhile, CSMA mineralization was introduced to provide sustained Ca^2+^ release and a biomimetic microenvironment favorable for osteogenesis [13]. We hypothesized that the integration of BP and mineralized CSMA matrix would establish a synergistically regulated microenvironment, in which photothermal stimulation and ionic cues cooperatively enhance antibacterial efficacy and osteogenic differentiation in a controllable manner.

Thus, the physicochemical properties of the composite hydrogels, including surface morphology, chemical composition, hydrophilicity, surface roughness, swelling ratio, mechanical properties, degradation profile and ions release kinetics, were systematically investigated. Their photothermal behavior and singlet oxygen generation were further examined to clarify the NIR-responsive antibacterial mechanism. *In vitro* antibacterial activity was assessed using *Staphylococcus aureus* (*S. aureus*) and *Escherichia coli* (*E. coli*), while cytocompatibility, cell adhesion, proliferation and osteogenic differentiation of BMSCs were evaluated to determine their biological performance. Furthermore, the underlying mechanisms were explored using RNA sequencing analysis. A rat calvarial defect model was used to evaluate the *in vivo* bone formation ability of BP@CSMA/CaCO_3_.

## Materials and methods

### Materials

CS powder (C766420) and calcium chloride (4 M, C915443) were obtained from Shanghai Macklin Biochemical Technology. MA (M102519), photoinitiator I2959, and sodium bicarbonate (S432111) were sourced from Aladdin Scientific Inc. Black phosphorus (100 μg/mL, 7723-14-0) was supplied by Hefei Keliao New Material Technology. Calcium (E-BC-K103-M) and Phosphorus (E-BC-K245-M) Colorimetric Assay Kits were obtained from Elabscience. Ammonium carbonate (10001418) came from Sinopharm Chemical Reagent. Singlet Oxygen Sensor Green (SOSG) was obtained from Meilunbio. *Staphylococcus aureus* and *E. coli* were provided by the China General Microbiological Culture Collection Center. Cell culture media, including growth medium (C11995500BT) and BMSC basal medium (BLDM-03011), were purchased from Cyagen Biosciences. The following kits and reagents were acquired from Beyotime Biotechnology: CCK-8 (C0039), Calcein-AM/PI Double Stain Kit (C2015S), DAPI (C1006), ALP Assay Kit (P0321S), and BCA Protein Assay Kit (P0010S). Rhodamine-phalloidin (40734ES75) was from Yeasen Biotechnology. The ALP Staining Kit (C3250S) was procured from Solarbio. TRIzol reagent (15596026CN) was obtained from Thermo Fisher Scientific. Isoflurane (R510-22-10) was purchased from Shenzhen Ruiwode Life Technology. H&E (G1076), Goldner’s trichrome (G1064), and Masson’s trichrome (G1006) staining kits were acquired from Wuhan Servicebio Technology.

### Synthesis of UV-responsive chitosan (CSMA)

**Figure 1:**
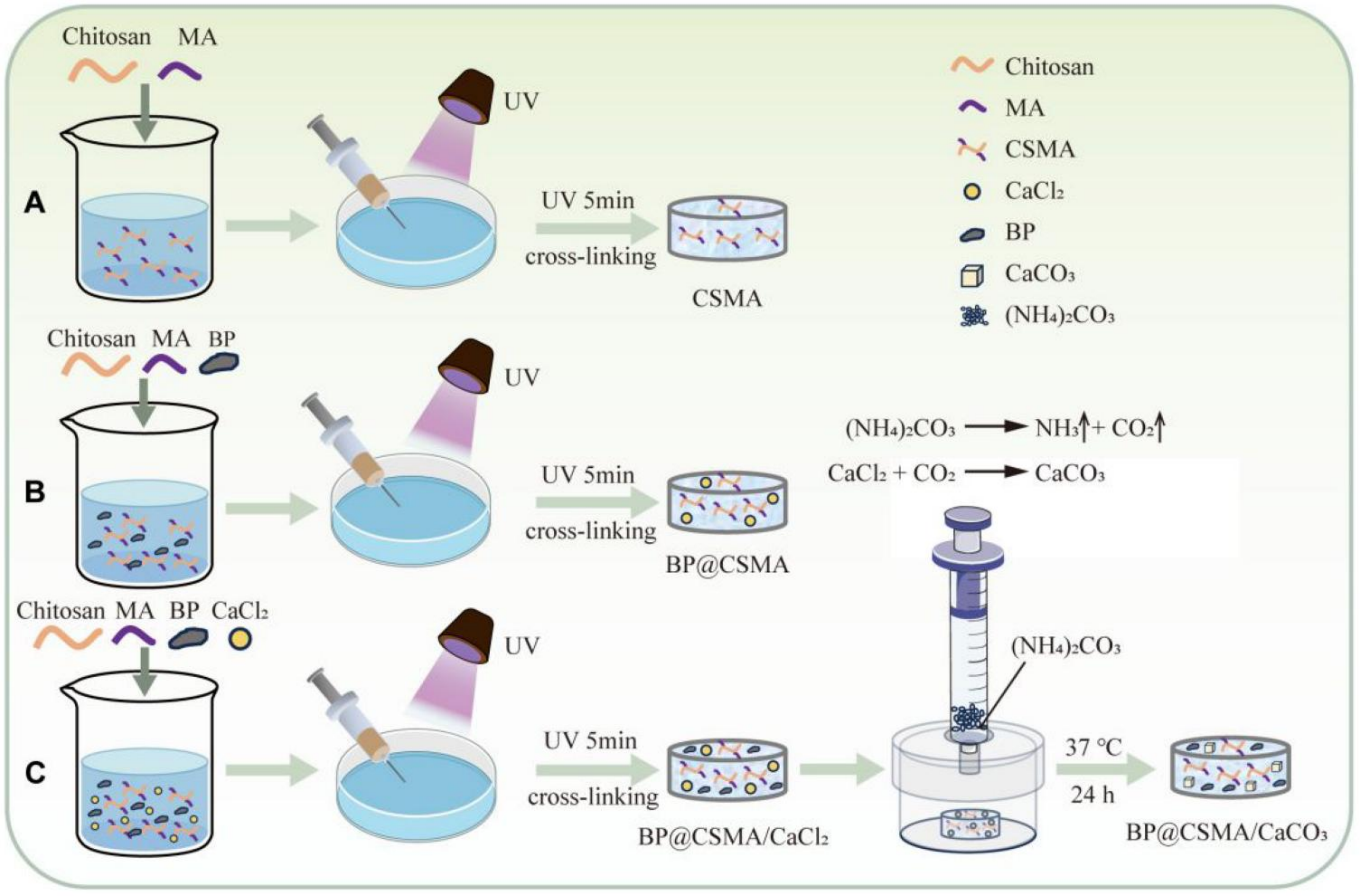
Schematic illustration of BP@CSMA/CaCO_3_ hydrogel preparation. (**A**) CSMA fabrication. (**B**) BP-loaded CSMA fabrication. (**C**) *In situ* mineralization to form BP@CSMA/CaCO_3_.

Based on preliminary optimization, a 3% (w/v) CSMA solution was prepared for this study. Briefly, CS powder was dissolved in 100 mL 1% (v/v) acetic acid solution to obtain a 3% (w/v) CS solution. MA solution was then added dropwise into the well-dissolved CS solution under constant stirring. The reaction was carried out at 60°C for 12 h, after which the mixture was adjusted to pH = 7 using NaHCO_3_ to terminate the reaction. The resulting product was dialyzed for 3 days to obtain the purified CSMA solution (Figure 1A).

### Preparation of BP-loaded CSMA hydrogels

CSMA solution (200 μL) was mixed with 0.2 wt.% photoinitiators and crosslinked by UV irradiation (GGY250, Wuxi Changya Lighting Co., Ltd., Wuxi, China) for 3 min to form CSMA hydrogel. BP (100 μg/mL) was added to CSMA solution containing I2959, stirred for 2 h, and subsequently crosslinked via UV irradiation for 3 min, known as BP@CSMA (Figure 1B). The BP@CSMA/CaCO_3_ hydrogel was synthesized through *in situ* mineralization of BP@CSMA hydrogel, according to our previous research. Briefly, CaCl_2_ solution (4 mol/L) was added into BP@CSMA mixture to provide Ca^2+^ ions, after which the solution was irradiated for 3 min to obtain the CSMA/CaCl_2_ precursor. Mineralization of CSMA/CaCl_2_ proceeded via CO_2_ diffusion, which was decomposed by (NH_4_)_2_CO_3._ The CO_2_ was gradually diffused into BP@CSMA/CaCl_2_ matrix, where it reacted with Ca^2+^ ions to form homogeneously dispersed CaCO_3_ within the hydrogel, resulting in the final BP@CSMA/CaCO_3_ composite (Figure 1C).

Freeze-dried samples were used for SEM observation, mechanical and degradation tests, osteogenesis evaluation and *in vivo* bone formation assessment. Hydrated gel samples were used for photothermal performance, antibacterial efficacy, cytotoxicity and cell proliferation assays.

### Characterization of the composite hydrogels

#### Microstructure of BP and composite hydrogels

The morphology of BP was observed by transmission electron microscopy (TEM; JEM-2100, JEOL Ltd., Tokyo, Japan). The microstructure and elemental distribution of lyophilized BP@CSMA and BP@CSMA/CaCO_3_ hydrogels were characterized by field-emission scanning electron microscopy equipped with energy-dispersive X-ray spectroscopy (FE-SEM/EDS; MERLIN, Carl Zeiss, Oberkochen, Germany). Prior to imaging, samples were sputter-coated with gold for 90 s and imaged at an acceleration voltage of 3 kV.

#### Chemical composition

The chemical composition of the samples was characterized by X-ray diffraction (XRD; PW1700, Philips, the Netherlands) and attenuated total reflectance-Fourier transform infrared spectroscopy (ATR-FTIR; Vertex, Bruker, USA). XRD measurements were performed using Cu-Kα radiation at 40 kV and 35 mA, with a scanning rate of 2° min^−1^ over the 2θ range of 10°–80°. ATR-FTIR spectra were collected in the wavenumber range of 675–4000 cm^−1^ at a resolution of 2 cm^−1^.

#### Surface roughness

Surface roughness of freeze-dried samples was assessed by using a laser scanning confocal microscope (VK-X100 series, Keyence Corporation, Osaka, Japan). In brief, the surface topography was scanned point by point using the system, and the corresponding roughness parameters were derived from the acquired height data.

#### Wettability

The surface wettability of the hydrogels was characterized by water contact angle measurements using a contact angle goniometer (DSA100s, Krüss GmbH, Hamburg, Germany). 2 μL droplet of deionized water was gently deposited onto the hydrogel surface, and the static contact angle was automatically determined through image analysis of the droplet profile.

#### Swelling ability

The weight of the lyophilized hydrogel was recorded as *W*_0_. The hydrogel was immersed in phosphate-buffered saline (PBS) at 37°C. At predetermined time intervals, the swollen hydrogel was removed, gently blotted to remove excess surface water, and weighed to obtain the wet weight (*W_t_*). The water absorption capacity (*W*_a_) was calculated using the following formula [35]:


$$W_{a}\;(\%)=\frac{W_{t}-W_{0}}{W_{0}}\times 100\%.$$

#### Mechanical properties

Compression tests were carried out using a universal testing machine (Instron 5967). Lyophilized samples were compressed at a constant rate of 1 mm/min, and the corresponding stress-strain curves were recorded. The compressive modulus was determined from the slope of the stress-strain curve.

#### Degradation behavior

The *in vitro* degradation behavior of CSMA, BP@CSMA and BP@CSMA/CaCO_3_ hydrogels was evaluated by a gravimetric method. Briefly, lyophilized hydrogel samples were weighed as the initial dry weight (W_0_) and immersed in 5 mL PBS containing 0.25 mg/mL lysozyme. The samples were incubated at 37°C and 100 rpm, and the medium was replaced every 2 days. At predetermined time points (1, 2, 3, 4, 5, 6, 7, 8, 9, 10, 14 and 21 days), samples were collected, gently rinsed with deionized water, lyophilized to constant weight and weighed as *W_x_*. The residual weight ratio (*Rr*) was calculated as:


$$Rr\;(\%)=\frac{Wx-W_{0}}{W_{0}}\times 100\%$$

### Photothermal properties of the composite hydrogels

The photothermal properties of BP@CSMA and BP@CSMA/CaCO_3_ hydrogels were evaluated under 808 nm NIR laser irradiation (HW808AD2000-34F, Shenzhen Fuzhe Fulei Technology Co., Ltd., China) at power densities of 1 or 2 W/cm^2^ for 5 min. For photothermal measurement, hydrated hydrogel samples were placed under non-immersed conditions, and the temperature was recorded directly from the exposed hydrogel surface using an infrared thermal camera (Research-N1, Hangzhou Meisheng Infrared Electro-Optic Technology Co., Ltd., China) at 1-min intervals during irradiation. Therefore, the measured temperature primarily reflected the surface photothermal response of the hydrogel samples rather than the temperature of the surrounding solution.

The photothermal stability of both hydrogels was evaluated through repeated heating-cooling cycles, consisting of 5 min of NIR irradiation at 2 W/cm^2^ followed by 3 min of natural cooling. The temperature profiles were recorded over three consecutive cycles to assess the durability of the photothermal response.

To provide a preliminary assessment of trans-tissue photothermal behavior, a 5-mm-thick agar block was used as a simplified tissue-mimicking barrier. The agar block was placed over the hydrogel samples, which were then irradiated with the 808 nm NIR laser at 2 W/cm^2^ for 5 min. The temperatures of both the upper agar surface and the hydrogel–agar interface were recorded to evaluate heat transfer across the hydrated agar layer.

### Ion release assay

The ion release behavior of the hydrogels was evaluated by measuring calcium (Ca^2+^) and inorganic phosphate (Pi) concentrations in the extracts. For Ca^2+^ release, extracts from BP@CSMA/CaCO_3_ hydrogels were collected on days 1, 3, 5, 7, 9, 11 and 13. Ca^2+^ concentration was quantified using a calcium colorimetric assay kit (E-BC-K103-M, Elabscience, Wuhan, China) at 610 nm. Ca^2+^ concentration was calculated based on the standard curve using the following equation:


$${\mathrm{Ca}}^{2+}=\frac{\Delta A_{610}-b}{a}\times f,$$

where Δ*A*_610_ represents the difference between sample and blank absorbance, *a* and *b* are the slope and intercept of the Ca^2+^ standard curve and *f* is the dilution factor.

For Pi release, extracts from BP@CSMA, BP@CSMA + NIR, BP@CSMA/CaCO_3_ and BP@CSMA/CaCO_3_ + NIR were collected at the same time points. The NIR-treated groups were irradiated with an 808 nm laser at 2 W/cm^2^ for 5 min. Pi concentration was measured using a Phosphorus Colorimetric Assay Kit (E-BC-K245-M, Elabscience, Wuhan, China) at 660 nm. P concentrations were calculated from the corresponding standard curves using the following equation:


$$\mathrm{Pi}=\frac{\Delta A_{660}-b}{a}\times 5\times f,$$

where Δ*A*_660_ represents the blank-corrected absorbance, *a* and *b* are the slope and intercept of the Pi standard curve, respectively, 5 is the kit conversion factor and *f* is the dilution factor.

### *In vitro* antibacterial assessments of the composite hydrogels

#### Antibacterial rates

The antibacterial activity of the composite hydrogels was evaluated using *S. aureus* and *E. coli*. The experiment included four groups: control (tissue-culture polystyrene (TCP)), CSMA, BP@CSMA and BP@CSMA/CaCO_3_, with or without NIR laser irradiation (2.0 W/cm^2^, 5 min). After sterilization at 115°C for 30 min, each sample was incubated with 500 μL bacterial suspension (1 × 10^6^ CFU/mL) at 37°C for 3 h. Adherent bacteria were detached by ultrasonication for 2 min, and 100 μL of the collected suspension was spread onto agar plates and incubated at 37°C for 24 h. The number of bacterial colonies was counted according to ISO 22196, and the antibacterial rate was calculated using the following formula [36]:


$$A\text{ntibacterial}\;\text{rate}\;(\%)=\frac{\text{CFU}\;\text{of}\;\text{control}\;\text{group}-\text{CFU}\;\text{of}\;\text{experimental}\;\text{group}}{\text{CFU}\;\text{of}\;\text{control}\;\text{group}}\times 100\%.$$

After NIR irradiation, the collected bacterial suspension was fixed with 2.5% glutaraldehyde for 2 h, washed with PBS, and dehydrated through a graded ethanol series for 30 min at each concentration. The bacterial morphology was then observed by SEM.

#### Singlet oxygen generation

Singlet oxygen generation was quantified using Singlet Oxygen Sensor Green (SOSG, Meilunbio). Hydrogels from five groups, including CSMA, BP@CSMA, BP@CSMA + NIR, BP@CSMA/CaCO_3_ and BP@CSMA/CaCO_3_ + NIR, were incubated with the SOSG working solution. The NIR-treated groups were exposed to an 808 nm laser at 2 W/cm^2^. At predetermined time points, the supernatant was collected, and fluorescence intensity was measured at an excitation wavelength of 504 nm and an emission wavelength of 525 nm to quantify singlet oxygen generation.

### *In vitro* biocompatibility of the composite hydrogels

#### The cytotoxicity

The cytotoxicity of BP@CSMA and BP@CSMA/CaCO_3_ was evaluated based on the international standard (ISO10993-5) [37]. Briefly, hydrogel extracts were collected after incubating each sample in growth medium at 37°C for 3 days. BMSCs were first cultured on TCP overnight, after which the culture medium was replaced with the corresponding extract medium. BMSCs cultured on TCP in fresh growth medium served as the negative control, representing the baseline culture condition and defined as 100% cell viability. After 24 and 48 h of exposure, cell viability was assessed using the CCK-8 assay by measuring absorbance at 450 nm. Live/dead staining was further performed using a calcein-AM/PI double-staining kit. At 24 and 48 h, cells were imaged under a confocal laser scanning microscope (CLSM, IX81-FV1000, Olympus Corporation, Tokyo, Japan).

Furthermore, to evaluate the influence of NIR irradiation on cell viability, BMSCs were seeded on BP@CSMA and BP@CSMA/CaCO_3_ hydrogels and cultured for 24 h, with or without NIR laser irradiation (1.0 W/cm^2^, 1 min). Cell viability was assessed using the CCK-8 assay at 24 h and 48 h post-irradiation. The relative cell viability was defined as the ratio of the OD value in irradiated samples to that in non-irradiated controls.

#### Cell proliferation rate

BMSCs were seeded onto the samples (CSMA, BP@CSMA and BP@CSMA/CaCO_3)_ at a density of 2 × 10^4^ cells per well. Cell proliferation was assessed on days 1, 3, 5 and 7 using the CCK-8 assay. NIR irradiation (1 W/cm^2^, 1 min) was applied to BP@CSMA and BP@CSMA/CaCO_3_ every three days. For cell adhesion and morphology observation, BMSCs were cultured on the samples for 12 h, fixed and stained with rhodamine-phalloidin and DAPI to visualize F-actin and nuclei, respectively. The stained cells were visualized using a confocal microscope.

### Osteogenesis of the composite hydrogels

The osteogenic differentiation of BMSCs cultured on CSMA, BP@CSMA and BP@CSMA/CaCO_3_ hydrogels was evaluated by quantifying ALP activity and the expression of osteogenesis-related genes, including osteocalcin (OCN) and osteopontin (OPN). The complete osteogenic medium consisted of BMSCs’ basal medium supplemented with 10% fetal bovine serum, 1% antibiotic-antimycotic, 3 mmol/L β-glycerophosphate, 50 μg/mL ascorbic acid, and 1 μmol/L dexamethasone (Dex). The mineralizing medium was defined as the same components but without Dex. In detail, the complete osteogenic medium was used as the positive control, whereas the Dex-free mineralizing medium was used to reduce the influence of strong exogenous osteogenic induction, thereby better revealing the intrinsic osteogenic effects of the hydrogels. β-Glycerophosphate and ascorbic acid were retained to support extracellular matrix maturation and mineral deposition. For photothermal stimulation, BP@CSMA and BP@CSMA/CaCO_3_ were exposed to 808 nm NIR irradiation at 1 W/cm^2^ for 1 min once every three days.

#### ALP activity

ALP activity is a well-established early-stage marker of osteogenic differentiation. Briefly, BMSCs were seeded on the composite hydrogels at a density of 2 × 10^4^ cells/mL and cultured in growth medium until reaching 60–70% confluence, after which the medium was replaced with mineralizing medium. On days 5 and 7, cells were washed with PBS, lysed with RIPA buffer, and centrifuged at 5000 rpm for 10 min. The supernatants were collected for ALP activity measurement using a commercial pNPP-based ALP assay kit. After incubation with pNPP substrate at 37°C for 30 min, the reaction was stopped, and absorbance was measured spectrophotometrically. ALP activity was calculated from a p-nitrophenol standard curve and normalized to total protein content determined by BCA assay.

Meanwhile, ALP staining was performed on BMSCs cultured on each sample according to a standard protocol. On days 5 and 7, cells were fixed with 10% neutral formalin, washed with PBS, and incubated with ALP staining solution in the dark for 20 min. After two additional PBS washes, images were acquired using an inverted microscope (DS-Ri2, Nikon Corporation, Tokyo, Japan) equipped with a color camera.

#### The expression of osteogenesis-related genes

Quantitative real-time reverse transcription polymerase chain reaction (qRT‑PCR) was performed to analyze the mRNA expression levels of OPN and OCN in BMSCs cultured on the samples on days 7 and 14. Total RNA was extracted using TRIzol reagent, and RNA concentration was measured spectrophotometrically. The primer sequences used for PCR amplification are listed in Supplementary Table S2. Gene expression levels were quantified using the 2^−ΔΔCT^ method.

### RNA sequencing analysis

BMSCs were cultured on CSMA and BP@CSMA/CaCO_3_ hydrogels for 7 days. The BP@CSMA/CaCO_3_ group was exposed to 808-nm NIR irradiation at 1 W/cm^2^ for 1 min once every three days. Total RNA was extracted using TRIzol reagent and subjected to RNA sequencing by Aksomics Biotech Co., Ltd. (Shanghai, China). Differentially expressed genes were identified using an adjusted *P*-value < 0.05 and an absolute fold change > 2 as the cutoff criteria.

### *In vivo* bone formation ability

#### Surgical procedures

Male Sprague–Dawley rats (8 weeks old, weighing 200 ± 15 g) were randomly divided into three groups: control (defects implanted with CSMA hydrogel only), BP@CSMA and BP@CSMA/CaCO_3_ (*n* = 6 per group). After induction of anesthesia via isoflurane inhalation, a circular calvarial defect (5 mm in diameter and 1.5 mm in depth) was created using a 5.0 mm trephine drill at 800 rpm under aseptic conditions. The corresponding hydrogel implants were placed into the defects, and the surgical area was rinsed with sterile saline. For BP@CSMA and BP@CSMA/CaCO_3_, postoperative photothermal treatment was administered using an 808-nm laser at 1 W/cm^2^ for 3 min at 3-day intervals for 2 weeks [38]. At 4 and 8 weeks after implantation, all animals were euthanized for subsequent radiographical and histological analyses.

#### Micro-CT analysis

Block sections containing the implanted samples and surrounding tissues were harvested and fixed in a 10% neutral formalin solution for 14 days. The samples were then scanned using a micro-CT system (VIVA-40, Scanco Medical AG, Bruttisellen, Switzerland), and the acquired images were reconstructed using TRI/3D-BON software. Morphometric parameters, including bone volume (BV), bone surface (BS) and trabecular thickness (Tb.Th), were quantified to evaluate new bone formation within the region of interest.

#### Histological analysis

Tissue blocks were dehydrated through a graded ethanol series, cleared in xylene twice for 1 h each, and embedded in paraffin wax. Serial sections of 40-μm thickness were cut along the coronal plane and subjected to HE staining, Goldner’s trichrome or Masson’s staining for histological observation. For Goldner’s trichrome staining, osteoid tissue was identified by its orange-red coloration, whereas mature bone appeared green. In terms of Masson staining, collagen fibers were highlighted in blue, and soft tissues were stained in red or orange.

### Statistical analysis

All data were depicted as mean ± standard deviation. Statistical significance among groups was determined by one-way ANOVA followed by Tukey’s *post hoc* test. The difference was considered statistically significant at ^*^P < 0.05 and ^**^P < 0.01.

## Results and discussion

### Characterization of BP and composite hydrogels

**Figure 2:**
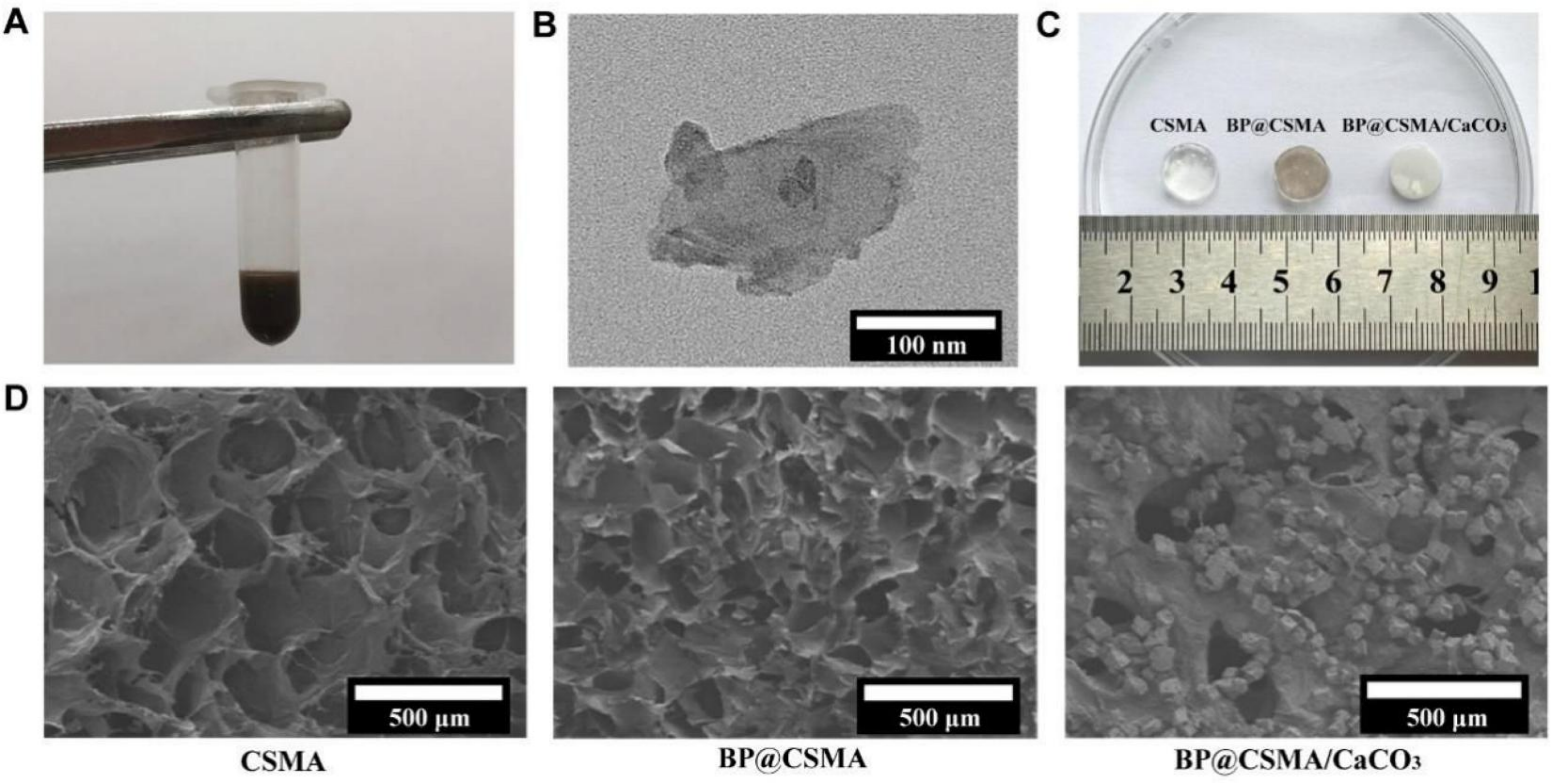
Morphological characteristics of BP and the composite hydrogels. (**A**) Representative photograph of BP dispersion. (**B**) Representative image of BP by TEM observation. (**C**) Representative photographs of lyophilized CSMA, BP@CSMA and BP@CSMA/CaCO_3_ hydrogels. (**D**) Representative SEM images of CSMA, BP@CSMA and BP@CSMA/CaCO_3_.

BP was well dispersed in the solution without obvious aggregation or sedimentation (Figure 2A). TEM image revealed a typical 2D layered morphology with a lateral size of approximately 215 nm (Figure 2B) [39, 40]. As shown in Figure 2C, lyophilized CSMA and CSMA/CaCO_3_ hydrogels appeared transparent and milky, respectively, whereas BP incorporation resulted in a visibly darker appearance. SEM images showed that all samples exhibited interconnected microporous structures (Figure 2D). Compared with CSMA, BP@CSMA displayed smaller pores, which might be related to interactions between BP and the CSMA matrix [41]. BP@CSMA/CaCO_3_ exhibited a porous architecture with embedded particulate deposits. These mineral aggregates, being randomly dispersed within the CSMA matrix, were identified as calcite crystals in our previous study [13]. EDS mapping (Supplementary Figure S1) revealed a homogeneous distribution of carbon (C), nitrogen (N) and oxygen (O) on the surface of the CSMA hydrogel. BP@CSMA exhibited an additional P signal with a content of 2.54%. In the BP@CSMA/CaCO_3_ sample, the elemental mapping demonstrated a uniform presence of C, N, O, P and calcium (Ca). The measured contents of P (2.77%) and Ca (25.85%) further verified the co-existence of BP and CaCO_3_ within the CSMA matrix.

**Figure 3:**
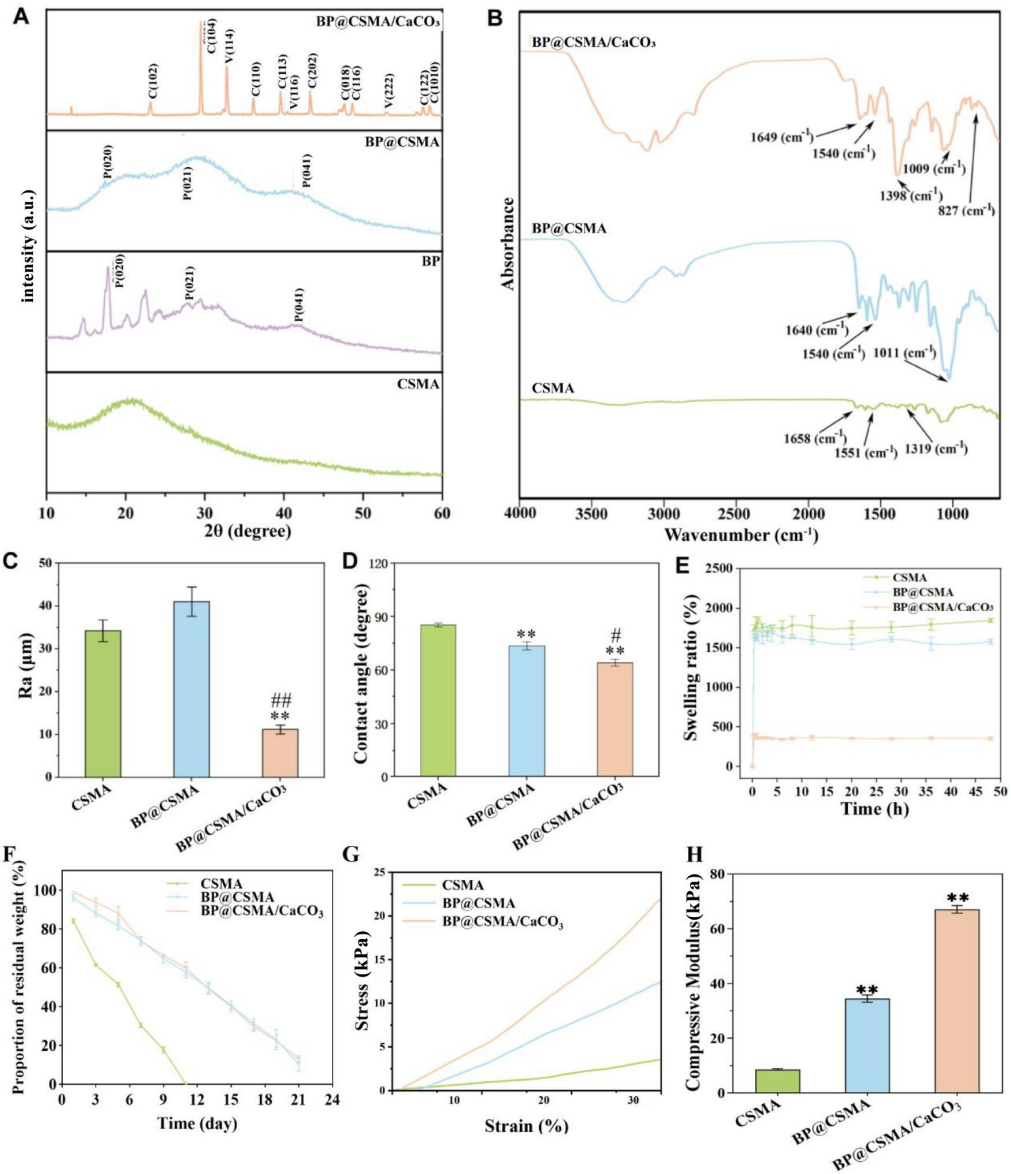
Physicochemical characterization of the composite hydrogels. (**A**) XRD pattern. (**B**) AIR-FTIR spectra. (**C**) Surface roughness. (**D**) Water contact angle. (**E**) Swelling ratio. (**F**) *In vitro* degradation behavior. (**G**) Compressive stress-strain curves. (**H**) Compressive modulus. Data are presented as mean ± SD, *n* = 3. *P < 0.01 vs. CSMA; ^#^*P* < 0.05 and ^##^*P* < 0.01 vs. BP@CSMA.

XRD and ATR-FTIR analyses were performed to verify the phase composition and chemical structure of the CSMA-based hydrogels. As shown in Figure 3A, CSMA exhibited a broad and diffuse peak at approximately 20.3°, indicating its amorphous structure associated with hydrogen bonding and N-acylation within the polymer network [36, 42]. In CSMA/CaCO_3_, distinct diffraction peaks at 23.5° and 29.7° were assigned to the (012) and (104) planes of calcite, respectively, confirming the successful mineralization of CaCO_3_ in the calcite phase [13]. In addition, characteristic BP peaks at 16.9°, 26.9° and 40.5° were observed in BP-containing samples, indicating that the BP crystal structure was retained within the CSMA hydrogel. ATR-FTIR spectra further confirmed the successful fabrication of BP@CSMA/CaCO_3_ (Figure 3B). The characteristic peaks of the CSMA matrix were the amide I (1658 cm^−1^), amide II (1551 cm^−1^) and amide III (1319 cm^−1^) bands [36]. Critically, the presence of CaCO_3_ was confirmed by the appearance of peaks at 1398 cm^−1^ (asymmetric stretching of CO_3_^2−^), 827 cm^−1^ (out-of-plane deformation of CO_3_^2−^), and 717 cm^−1^ (in-plane deformation of O–C–O) [13]. Furthermore, the BP incorporation was supported by a new peak at 1009 cm^−1^, which was attributed to the P=O stretching vibration [43].

The average surface roughness (Ra) values of CSMA, BP@CSMA and BP@CSMA/CaCO_3_ were measured as 34.22 ± 2.55 μm, 41.02 ± 3.43 μm and 11.19 ± 1.03 μm, respectively (Figure 3C). CaCO_3_ mineralization markedly reduced Ra, which might be attributed to mineral deposits filling the porous structure and producing a more homogeneous surface [44]. In contrast, the introduction of BP led to a moderate increase in Ra values, suggesting that the distinct morphology of BP contributed to greater surface texture in the composite hydrogel [45].

BP@CSMA showed enhanced surface wettability compared with CSMA, while BP@CSMA/CaCO_3_ exhibited the strongest hydrophilicity (Figure 3D). The initial enhancement in wettability from CSMA to BP@CSMA was primarily governed by an increase in surface roughness, which amplified the intrinsic hydrophilicity of the polymer matrix. The stronger hydrophilicity of BP@CSMA/CaCO_3_ might be attributed to the mineralized layer formed during biomineralization, which created a more wettable surface interface.

Adequate swelling is important for hydrogels to maintain a moist microenvironment and support nutrient diffusion [46]. As illustrated in Figure 3E, all samples exhibited rapid swelling within the first 2 h, followed by a slower swelling phase by 50 h. Overall, BP@CSMA/CaCO_3_ exhibited significantly lower swelling ratios compared to CSMA and BP@CSMA. In this study, CaCO_3_ particles served as a physical filler within the CSMA network, occupying pore spaces and thereby reducing the available volume for water absorption and retention [47, 48]. Furthermore, the addition of BP and CaCO_3_ effectively reinforced the polymer network, enhancing the cross-linking density and structural integrity, which collectively restricted hydrogel expansion and led to the observed reduction in swelling capacity [49, 50].

For degradation behavior, CSMA degraded rapidly and was almost completely degraded within approximately 11 days. In contrast, BP@CSMA and BP@CSMA/CaCO_3_ exhibited slower degradation profiles, with approximately 10 and 15% of residual weight remaining at day 21, respectively. The delayed degradation of BP@CSMA might be attributed to the reinforcement of BP within the CSMA network [51, 52]. BP acted as physical nanofillers and interacted with CSMA chains, thereby improving network stability. In BP@CSMA/CaCO_3_, the mineralized phase might further occupy pores and strengthen the hydrogel matrix [44].

The mechanical properties of CSMA, BP@CSMA and BP@CSMA/CaCO_3_ hydrogels were evaluated by compression testing. As shown in Figure 3G, BP@CSMA/CaCO_3_ exhibited higher compressive stress than BP@CSMA and CSMA under the same strain. Consistently, the compressive modulus increased from 8.62 ± 0.20 kPa for CSMA to 34.48 ± 1.09 kPa for BP@CSMA, and further to 67.10 ± 1.12 kPa for BP@CSMA/CaCO_3_ (Figure 3H), indicating progressively enhanced mechanical strength. The improvement in BP@CSMA might be related to the reinforcing role of BP within the CSMA matrix, as BP has been reported to strengthen hydrogel-based constructs by facilitating stress transfer within polymer networks [51]. The introduction of CaCO_3_ provided rigid inorganic phases into the hydrogel, further increasing its resistance to deformation, consistent with previous reports on CaCO_3_-reinforced hydrogels [53].

Ca^2+^ release from BP@CSMA/CaCO_3_ was measured to verify the ion-supplying capacity of the mineralized hydrogel. As shown in Supplementary Figure S2A, BP@CSMA/CaCO_3_ exhibited sustained Ca^2+^ release over time. The cumulative Ca^2+^ concentration increased from 0.49 ± 0.11 mmol/L on day 1 to 2.61 ± 0.13 mmol/L on day 13, indicating a gradual release behavior. This sustained release was likely derived from the progressive dissolution of the mineralized CaCO_3_ phase, which might contribute to the formation of a favorable ionic microenvironment for osteogenic differentiation.

As shown in Supplementary Figure S2B, BP@CSMA exhibited continuous Pi accumulation, reaching approximately 65 mmol/L by day 13, whereas BP@CSMA/CaCO_3_ released a lower amount of Pi, approximately 45.3 ± 4.63 mmol/L at the same time point. After NIR irradiation, Pi release was markedly promoted, increasing to approximately 95 mmol/L in BP@CSMA and 61.89 ± 3.67 mmol/L in BP@CSMA/CaCO_3_ by day 13. This NIR-enhanced Pi release indicated that photothermal stimulation might accelerate BP degradation and phosphorus diffusion. The lower Pi release from BP@CSMA/CaCO_3_ compared with BP@CSMA suggested that CaCO_3_ mineralization could modulate the Pi release behavior within the hydrogel matrix.

### Photothermal performance of BP-loaded hydrogels

**Figure 4:**
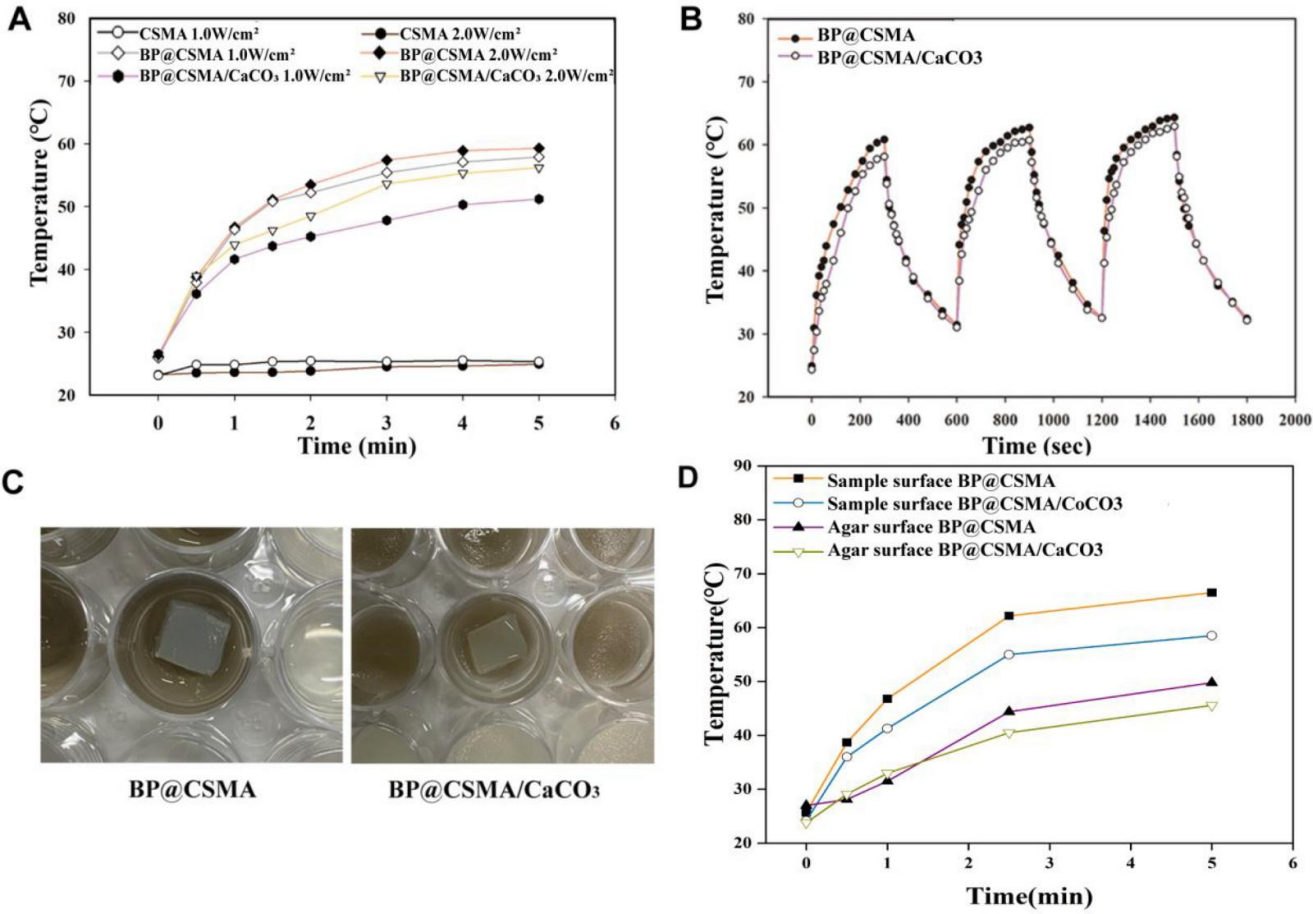
Photothermal performance of the composite hydrogels. (**A**) Temperature profiles of CSMA, BP@CSMA and BP@CSMA/CaCO_3_ under 808-nm NIR irradiation at different laser power densities. (**B**) Photothermal stability of BP@CSMA and BP@CSMA/CaCO_3_ over three heating-cooling cycles under 808-nm NIR irradiation at 2 W/cm^2^. (**C**) Representative photographs of BP@CSMA and BP@CSMA/CaCO_3_ covered with a 5-mm-thick agar block as a simplified tissue-mimicking barrier. (**D**) Temperature profiles of the corresponding samples irradiated through the agar barrier under 808-nm NIR irradiation at 2 W/cm^2^. Data are presented as mean ± SD, *n* = 3.

BP typically exhibits excellent photothermal capacity under NIR irradiation [54]. As shown in Figure 4A, the temperatures of CSMA remained near ambient temperature (∼20°C) under both power densities. In contrast, BP@CSMA and BP@CSMA/CaCO_3_ displayed a rapid temperature increase from 20°C to approximately 60°C under 2 W/cm^2^ NIR irradiation, reaching final temperatures of 59.3°C and 56.1°C after 5 min, respectively. Moreover, the temperature increase was power- and time-dependent, suggesting that the photothermal response could be regulated by adjusting NIR parameters. It was noteworthy that CaCO_3_ reduced the photothermal conversion efficiency of BP, presumably due to increased light scattering and reflection within the mineral-composite matrix (Figure 4A and D) [55]. Additionally, BP@CSMA and BP@CSMA/CaCO_3_ also demonstrated consistent photothermal stability over three on/off heating-cooling cycles (Figure 4B). It should be noted that the photothermal test was based on direct surface temperature measurement of the hydrated hydrogels rather than on bulk solution temperature. Therefore, the results mainly reflected the intrinsic photothermal response of the hydrogel surface. Although released BP in a surrounding liquid phase could theoretically contribute to NIR-induced heating, such an effect was expected to be minimal under the present measurement conditions.

To preliminarily evaluate trans-tissue photothermal performance, a 5-mm-thick agar block was used as a simplified tissue-mimicking barrier (Figure 4C). Agar phantoms are widely used in biomedical optical and thermal studies owing to their water-rich composition, reproducibility, and tunable properties [56]. Under this condition, the temperatures measured at the hydrogel surfaces remained close to those obtained without the agar barrier, indicating that agar did not markedly attenuate the photothermal effect of BP@CSMA or BP@CSMA/CaCO_3_. This might be attributed to the effective penetration of 808-nm NIR light within the biological window [57]. A clear thermal gradient was observed, with the upper agar surface reaching 40–45°C and the hydrogel–agar interface reaching 55–65°C (Figure 4D), confirming efficient photothermal conversion and heat conduction through the hydrated agar matrix. Importantly, this profile suggested that bactericidal temperatures could be maintained at the implant surface while the superficial tissue-facing side remained at a relatively tolerable temperature (∼40°C) [58].

### *In vitro* antibacterial performance of the composite hydrogels

**Figure 5:**
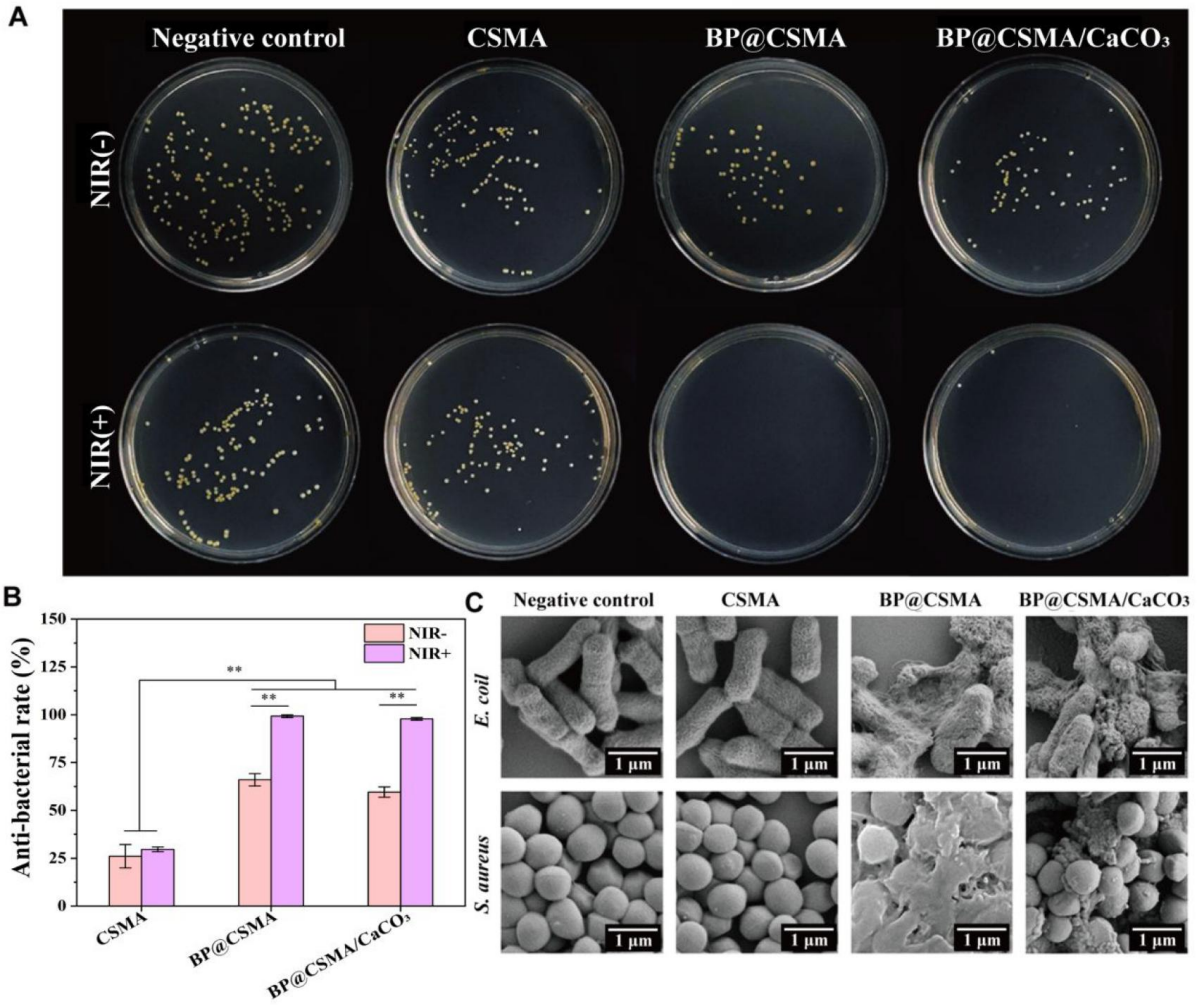
Antibacterial performance of the composite hydrogels. (**A**) Representative images of *S. aureus* colonies collected from hydrogels with or without NIR irradiation (808 nm, 2 W/cm^2^, 5 min). (**B**) Quantitative antibacterial rates calculated from colony-forming unit (CFU) counts. (**C**) Representative SEM images of bacterial morphologies on the composite hydrogels. Data are presented as mean ± SD, *n* = 3. ^**^P < 0.01 indicate significant differences between groups.

As shown in Figure 5A and B, CSMA exhibited moderate antibacterial activity compared with the negative control group, which could be attributed to the inherent antibacterial properties of CS. Upon incorporation of BP, the antibacterial rates of BP@CSMA and BP@CSMA/CaCO_3_ increased to 66.03 and 59.57%, respectively, even without NIR irradiation. This improvement might be related to the inherent antibacterial activity of BP, including physical disruption of bacterial membranes by its ultrathin layered structure and sharp edges [59]. SEM images further showed distorted and ruptured bacterial membranes after exposure to BP-containing hydrogels (Figure 5C). Under NIR irradiation, the corresponding antibacterial rates reached 99.28 and 97.84%, exhibiting superior antibacterial effects. This enhanced antibacterial effect was mainly associated with BP-mediated photothermal heating, which rapidly increased the local temperature to approximately 60°C and induced bacterial damage through protein denaturation and membrane disruption [60].

To further clarify the antibacterial mechanism, singlet oxygen generation was quantified using SOSG fluorescence (Supplementary Figure S3). The CSMA group showed only a weak and stable background signal, whereas BP-containing hydrogels exhibited gradually increased fluorescence intensity, indicating that BP contributed to singlet oxygen generation in composite hydrogels. Upon NIR irradiation, both BP@CSMA and BP@CSMA/CaCO_3_ displayed enhanced fluorescence signals, confirming the NIR-responsive ROS-generating capability of BP. Notably, the signal intensity of BP@CSMA was higher than that of BP@CSMA/CaCO_3_ under the same conditions, which might be attributed to the modulatory effect of CaCO_3_ mineralization on BP photoactivity. Therefore, the antibacterial activity of BP-containing hydrogels under NIR irradiation can be attributed to the combined effects of photothermal heating and ROS generation [28, 61].

### *In vitro* biocompatibility of the composite hydrogels

The cytocompatibility of BP@CSMA and BP@CSMA/CaCO_3_ was illustrated in Supplementary Figure S4. All samples showed cell viabilities above 90%, exceeding the 70% threshold required by ISO 10993-5 (Supplementary Figure S4A) [62]. Live/dead staining was consistent with the CCK-8 results (Supplementary Figure S4C). The presence of live cells (green) in all groups indicated that BP@CSMA and BP@CSMA/CaCO_3_ were non-cytotoxic. To verify the suitability of the NIR parameter used for osteogenic stimulation, the effects of irradiation at 1 W/cm^2^ for 1 min on BMSC viability were evaluated (Supplementary Figure S4B). An obvious reduction in cell viability was observed at 24 h after irradiation in both BP@CSMA and BP@CSMA/CaCO_3_, which might be attributed to the acute photothermal effect and singlet oxygen generated by BP under NIR exposure [63]. However, cell viability recovered significantly by 48 h, particularly in the BP@CSMA/CaCO_3_, indicating that this irradiation condition did not cause irreversible cytotoxicity. Combined with the enhanced cell proliferation and osteogenic differentiation observed in subsequent assays, these findings supported that 1 W/cm^2^ for 1 min represented an appropriate mild photothermal stimulation parameter for promoting osteogenesis while maintaining acceptable cytocompatibility, which was in line with previous studies [64, 65].

**Figure 6:**
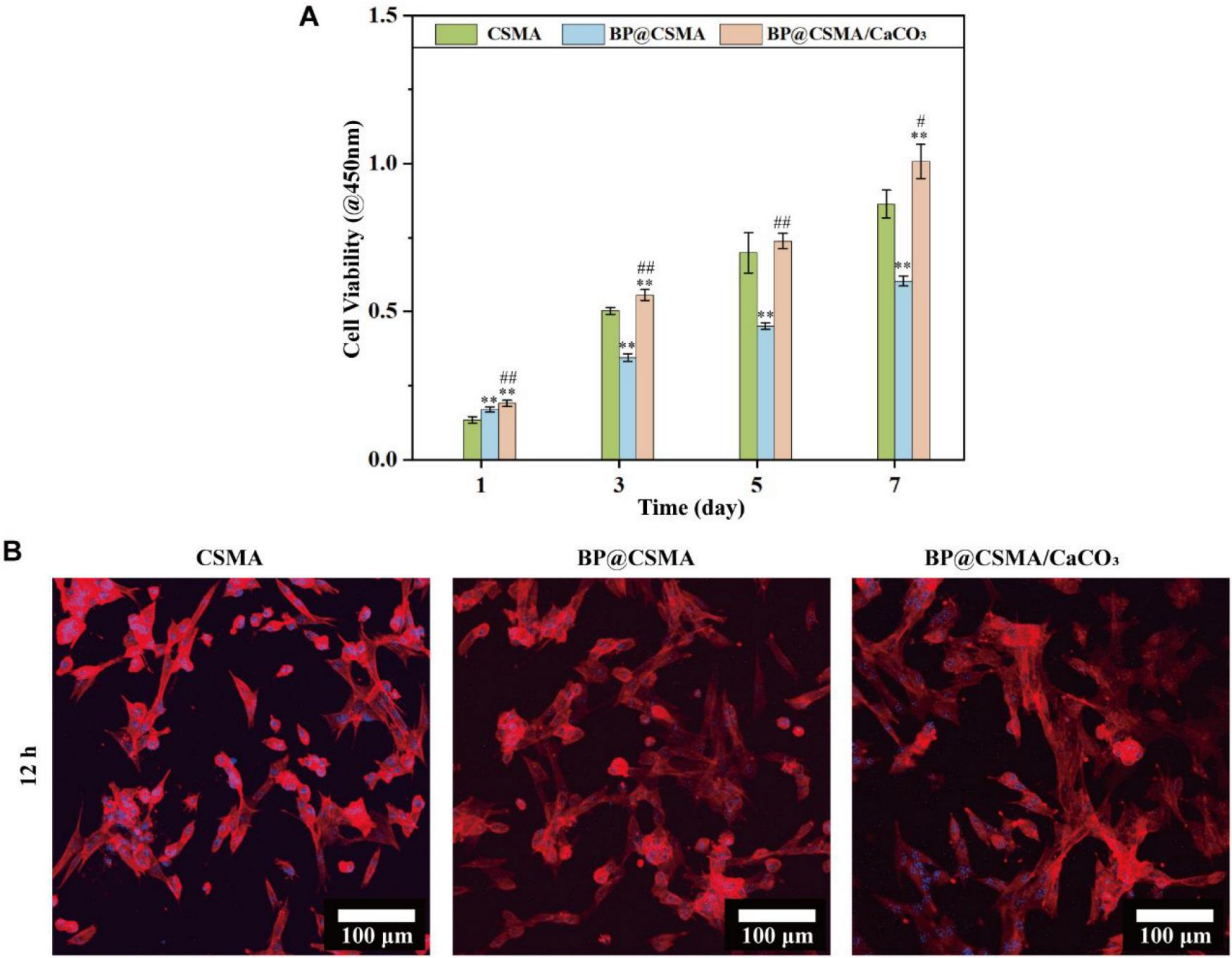
Proliferation and morphology of BMSCs cultured on composite hydrogels. (**A**) Quantitative analysis of BMSC proliferation on CSMA, BP@CSMA and BP@CSMA/CaCO_3_ hydrogels for 7 days using CCK-8 assay. (**B**) Representative fluorescence images of BMSCs cultured on CSMA, BP@CSMA and BP@CSMA/CaCO_3_ hydrogels for 12 h. F-actin was stained with rhodamine-phalloidin, and nuclei were stained with DAPI. Data are presented as mean ± SD, *n* = 3. ^**^P < 0.01 vs. CSMA; ^#^*P* < 0.05 and ^##^*P *< 0.01 vs. BP@CSMA.

The initial adhesion and subsequent proliferation of cells at the material–tissue interface are critical for the early stages of bone regeneration. As shown in Figure 6B, BMSCs displayed characteristic cytoskeletal morphology and were evenly distributed across all hydrogel surfaces. Notably, cells cultured on BP@CSMA/CaCO_3_ exhibited more developed actin filaments with extensive filopodia extensions. The formation of this interconnected cellular network demonstrated favorable adhesion and effective spreading on the composite hydrogel surface.

Since NIR irradiation was confirmed to have negligible side effects on cell viability (Supplementary Figure S4B), the photothermal treatment (1 W/cm^2^, 1 min) was applied to all groups at 3-day intervals. Over a 7-day culture period, all groups exhibited time-dependent increases (Figure 6A). On day 1, both BP-loaded hydrogels exhibited significantly higher proliferation rates than the CSMA group. By day 3, BP@CSMA showed a relatively low proliferation rate, while BP@CSMA/CaCO_3_ maintained a consistent increase, exhibiting significantly higher metabolic activity than both BP@CSMA and CSMA. Since day 5, BP@CSMA/CaCO_3_ and CSMA groups exhibited similar proliferation rates, both significantly exceeding that of BP@CSMA. Notably, on day 7, BP@CSMA/CaCO_3_ achieved the highest proliferation level, reaching approximately 1.7-fold that of the BP@CSMA group. The relatively lower proliferation observed in the BP@CSMA might be mainly related to its higher photothermal response under the same NIR irradiation conditions. As discussed above, BP@CSMA reached a higher temperature than BP@CSMA/CaCO_3_, which might impose greater thermal stress on adherent cells during the early stage of culture. In contrast, the incorporation of CaCO_3_ moderated the photothermal effect and simultaneously provided a more favorable mineral-associated microenvironment, which together supported the enhanced cell proliferation in the BP@CSMA/CaCO_3_.

### Osteogenesis of the composite hydrogels

**Figure 7:**
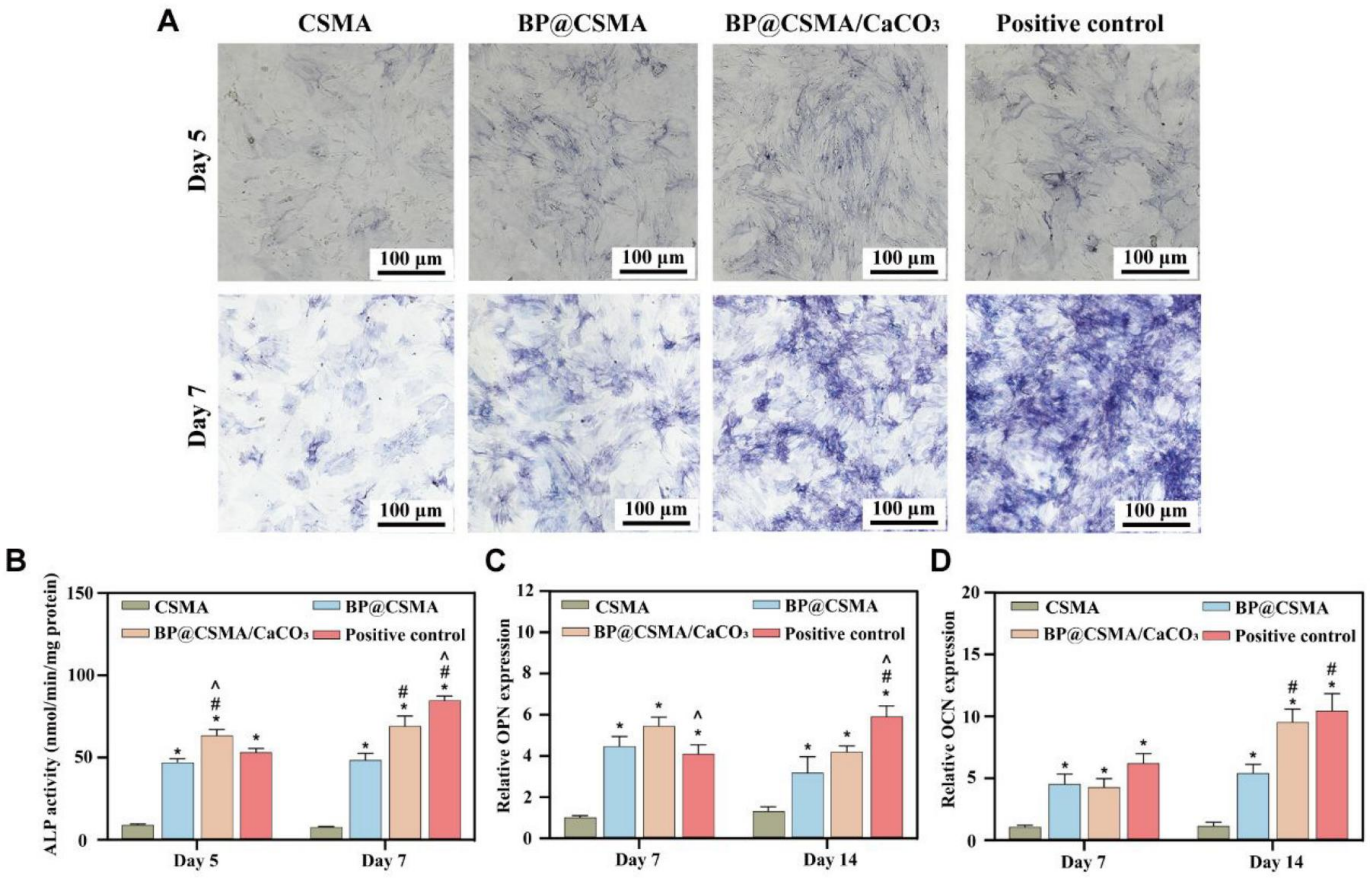
Osteogenic differentiation of BMSCs cultured on composite hydrogels. (**A**) Representative ALP staining images of BMSCs cultured on CSMA, BP@CSMA and BP@CSMA/CaCO_3_ hydrogels. (**B**) Quantitative analysis of ALP activity normalized to total protein content. (**C**, **D**) Relative mRNA expression levels of OPN and OCN in BMSCs cultured on CSMA, BP@CSMA, and BP@CSMA/CaCO_3_ hydrogels, determined by RT-qPCR. Data are presented as mean ± SD, *n* = 3. *P < 0.05 vs. CSMA; ^#^*P *< 0.05 vs. BP@CSMA; ^^^*P *< 0.05 vs. BP@CSMA/CaCO_3_.

ALP, a secreted enzyme produced during early osteogenic differentiation of stem cells, plays a critical role in bone regeneration [66]. Semi-quantitative analysis of ALP activity in BMSCs cultured on all samples was presented in Figure 7B. On day 5, CSMA showed the lowest ALP activity due to its lack of intrinsic osteoinductive properties. In contrast, BP@CSMA/CaCO_3_ exhibited significantly higher ALP levels than all other groups. By day 7, the positive control demonstrated the most pronounced increase in ALP activity, reaching significantly higher levels when compared with BP@CSMA and CSMA. Similarly, BP@CSMA/CaCO_3_ achieved nearly 1.3-fold higher levels than BP@CSMA. Additionally, the result of ALP staining (Figure 7A) was consistent with these semi-quantitative analyses, indicating that both BP@CSMA and BP@CSMA/CaCO_3_ promoted ALP expression in BMSCs, suggesting their beneficial role in facilitating early osteogenic differentiation.

OPN, a secreted and phosphorylated glycoprotein, facilitates the deposition of mineralized extracellular matrix, which demonstrates a positive correlation with osteogenic activity [67]. As early as day 7, both BP@CSMA and BP@CSMA/CaCO_3_ exhibited elevated OPN expression compared with CSMA. BP@CSMA/CaCO_3_ reached its peak OPN level on day 7, reaching approximately 1.3-fold that of the positive control group (Figure 7C). By day 14, OPN expression in BP@CSMA and BP@CSMA/CaCO_3_ had decreased slightly, whereas the positive control exhibited a continuous increase and became significantly higher than the remaining groups. The decline in OPN levels by day 14 in BP@CSMA and BP@CSMA/CaCO_3_ might be explained by its role in biomineralization. This temporal change might reflect the dynamic role of OPN during osteogenic differentiation. The early upregulation of OPN in BP-containing hydrogels suggested accelerated initiation of matrix mineralization, while the subsequent decrease might be associated with progression toward a more mature mineralized matrix.

Regarding OCN, which is a marker of late-stage osteogenic differentiation secreted by mature osteoblasts [67], the positive control exhibited the highest level on day 7, and continued to increase until day 14. The BP@CSMA displayed similar OCN levels at two time points (Figure 7D). Whereas BP@CSMA/CaCO_3_ group displayed a steady rise in OCN expression, reaching approximately twice the level of the BP@CSMA group by day 14. These results suggested that BP promoted BMSC osteogenic differentiation under mild NIR irradiation, which was further supported by ion release from the mineralized hydrogel. BP-mediated mild photothermal stimulation may activate thermal-response pathways and enhance osteogenic marker expression. In parallel, Ca^2+^ released from CaCO_3_ and phosphorus from BP degradation provided complementary ionic cues for matrix mineralization and osteogenic regulation. Thus, BP@CSMA/CaCO_3_ created a coordinated microenvironment that combined photothermal stimulation with Ca^2+^/Pi-related ionic support to promote bone regeneration.

It should be noted that all hydrogel groups were evaluated in a reduced-induction mineralizing medium without dexamethasone, whereas the positive control was cultured in complete osteogenic medium. This design was intended to minimize the dominant osteoinductive effect of Dex, thereby allowing a clear assessment of the intrinsic osteogenic contribution of the hydrogels. Since all hydrogel groups were cultured under the same medium conditions, the differences observed among CSMA, BP@CSMA, and BP@CSMA/CaCO_3_ were mainly attributed to BP incorporation, CaCO_3_ mineralization and their combined regulation of the cellular microenvironment.

Although BP-based materials and mineralized CSMA systems have both been reported previously [25, 68], the present study was designed for infected bone defect repair, where antibacterial activity and osteogenic support were needed at different stages. In our system, BP served as the NIR-responsive component, while the introduction of CaCO_3_ did more than provide mineral support. It also changed the physicochemical properties of the hydrogel, including its mechanical behavior, degradation behavior, phosphorus release profile and photothermal response. As a result, the role of CaCO_3_ was not limited to simple composition addition but extended to regulating the overall microenvironment and biological performance of the composite hydrogel.

### Potential regulatory mechanism of osteogenesis effect of BP@CSMA/CaCO_3_

**Figure 8:**
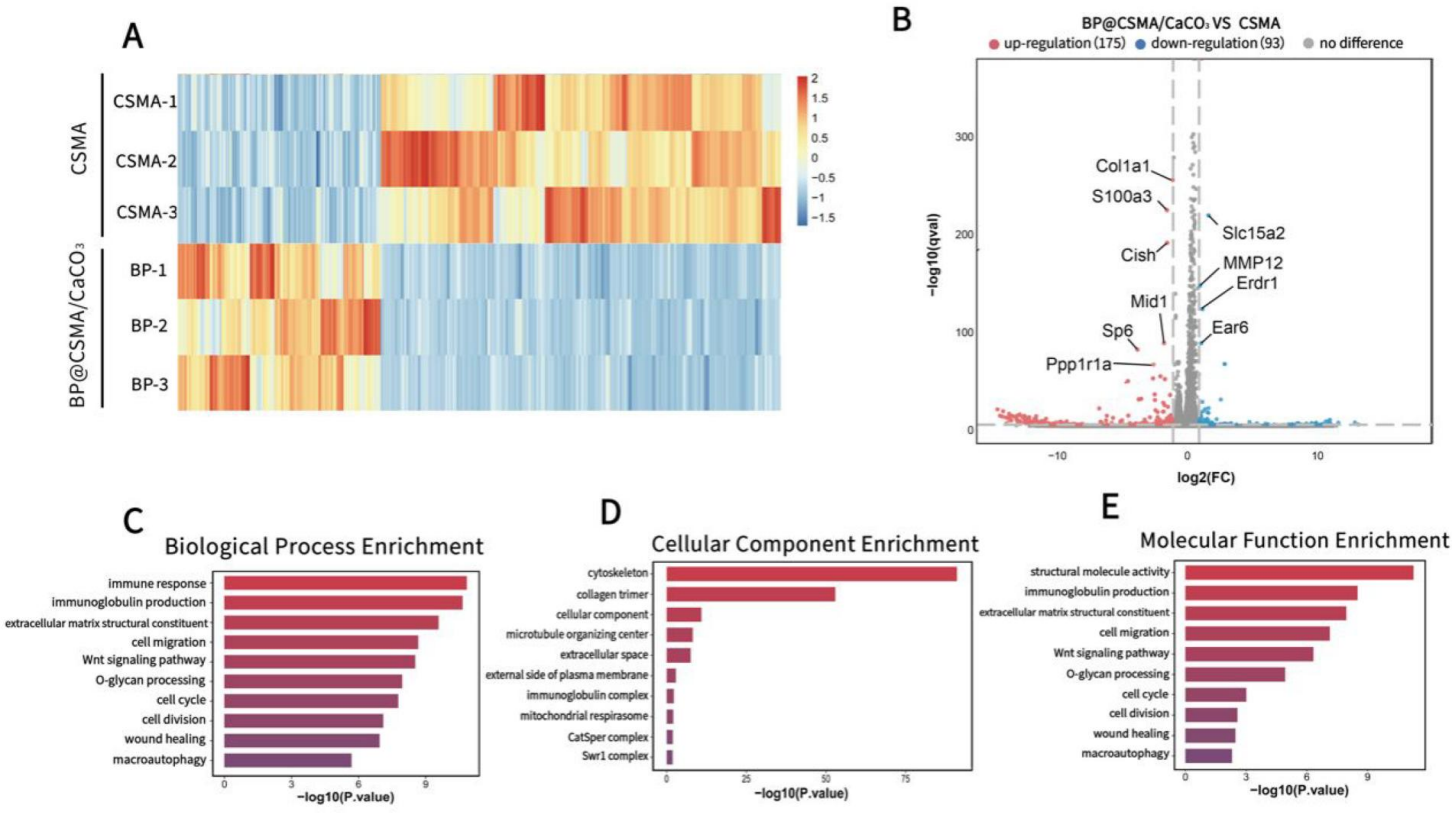
RNA sequencing analysis of BMSCs cultured on composite hydrogels. (**A**) Heatmap of differentially expressed genes between the CSMA and BP@CSMA/CaCO_3_ groups. (**B**) Volcano plot of upregulated and downregulated genes in BP@CSMA/CaCO_3_ compared with CSMA; (**C**–**E**) gene ontology (GO) enrichment analysis of differentially expressed genes, including biological process, cellular component and molecular function categories.

To elucidate the mechanism by which BP@CSMA/CaCO_3_ enhanced osteogenic differentiation, RNA sequencing was performed to identify differentially expressed genes in BMSCs cultured on BP@CSMA/CaCO_3_ compared with the CSMA (Figure 8A). As shown in Figure 8B, 175 genes were upregulated, and 93 genes were downregulated in the BP@CSMA/CaCO_3_ group. Gene Ontology (GO) enrichment analysis revealed that the upregulated genes were primarily associated with immune response, extracellular matrix structural constitution, and cell migration (Figure 8C). Molecular functions critically involved in osteogenic regeneration, including structural molecule activity, extracellular matrix structural constituent binding, immunoglobulin receptor binding, and beta-tubulin binding, were significantly enriched (Figure 8E) [69]. Regarding cellular components, BP@CSMA/CaCO_3_ exhibited pronounced regulation of cytoskeletal elements, collagen trimers, and other key cellular structures (Figure 8D).

### Bone formation ability of the composite hydrogels

**Figure 9:**
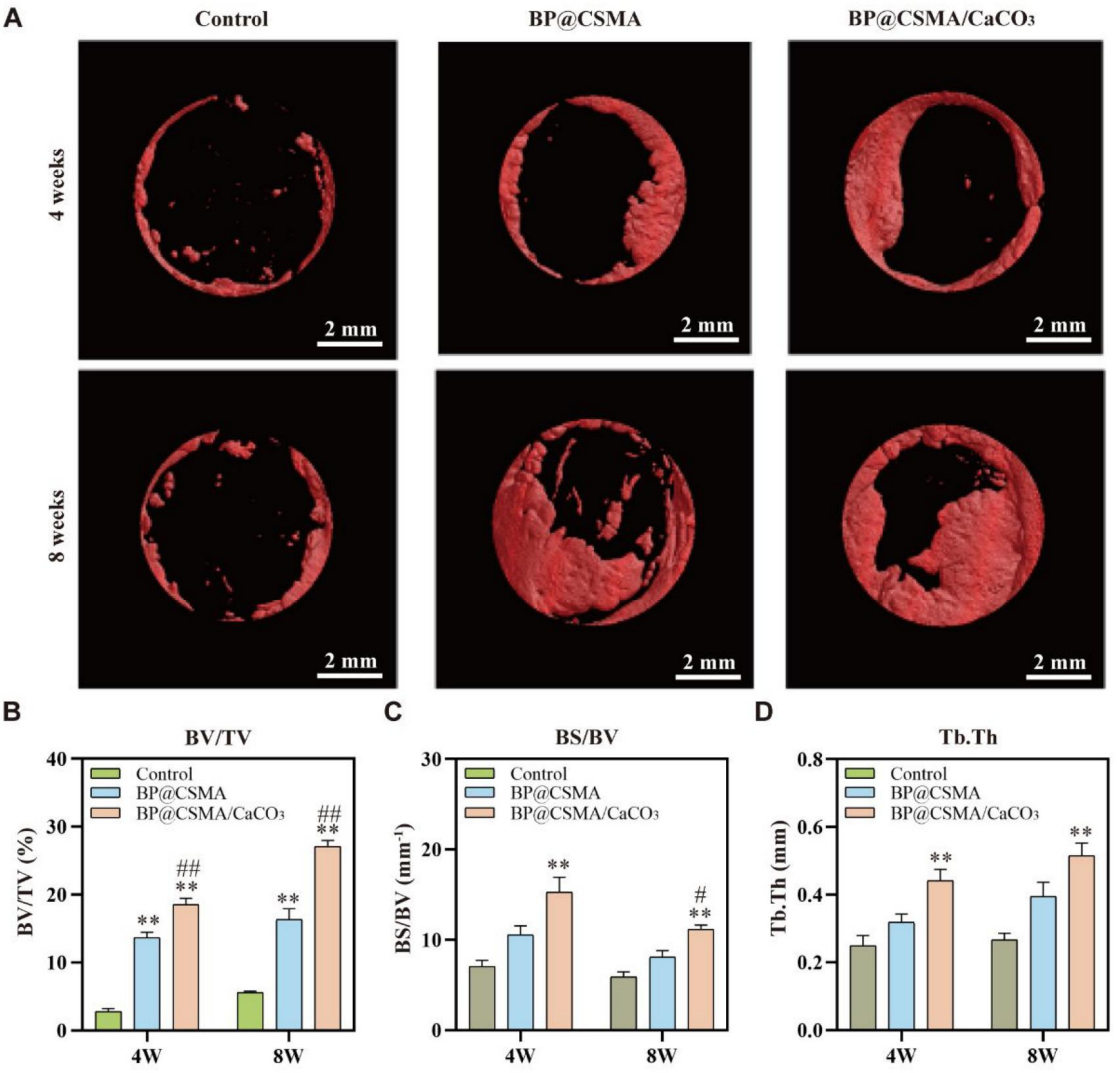
*In vivo* bone regeneration capacity of composite hydrogels by micro-CT. (**A**) Representative micro-CT images of calvarial defects in the control, BP@CSMA and BP@CSMA/CaCO_3_ groups at 4 and 8 weeks after implantation. (**B**–**D**) Quantitative micro-CT analysis of bone volume fraction (BV/TV), bone surface density (BS/BV) and trabecular thickness (Tb.Th), respectively. Data are presented as mean ± SD, *n* = 6. ^**^P < 0.01 vs. control; ^#^*P *< 0.05 and ^##^*P *< 0.01 vs. BP@CSMA.

The bone regeneration capacity was initially evaluated by micro-CT at 4 and 8 weeks post-implantation (Figure 9A). At 4 weeks, bone remodeling was primarily observed at the margins of the original defect across all groups. The control group displayed largely open defects with only 16.7% of the area filled by newly formed bone and had little change by the 8-week time point. In contrast, both the BP@CSMA and BP@CSMA/CaCO_3_ groups exhibited a time-dependent increase in new bone formation. Notably, the BP@CSMA/CaCO_3_ group demonstrated the most substantial bone regeneration, with new bone extending from the defect margins toward the center and progressively filling the implantation site.

Both BP@CSMA and BP@CSMA/CaCO_3_ exhibited significantly higher BV/TV values than the control group at both 4 and 8 weeks, with the highest value observed in the BP@CSMA/CaCO_3_ (Figure 9B). The BS/BV value of BP@CSMA/CaCO_3_ was also significantly higher than that of the other groups, especially at 4 weeks (Figure 9C), which might reflect abundant newly formed BSs and active early remodeling within the defect region. In addition, Tb.Th in the BP@CSMA/CaCO_3_ was significantly greater than that in the control group at both time points (Figure 9D). The further increases in BV/TV and Tb.Th at 8 weeks suggested progressive trabecular maturation and enhanced mineralized bone deposition. Together with the micro-CT reconstruction results, these findings supported the superior bone regeneration capacity of BP@CSMA/CaCO_3_.

**Figure 10:**
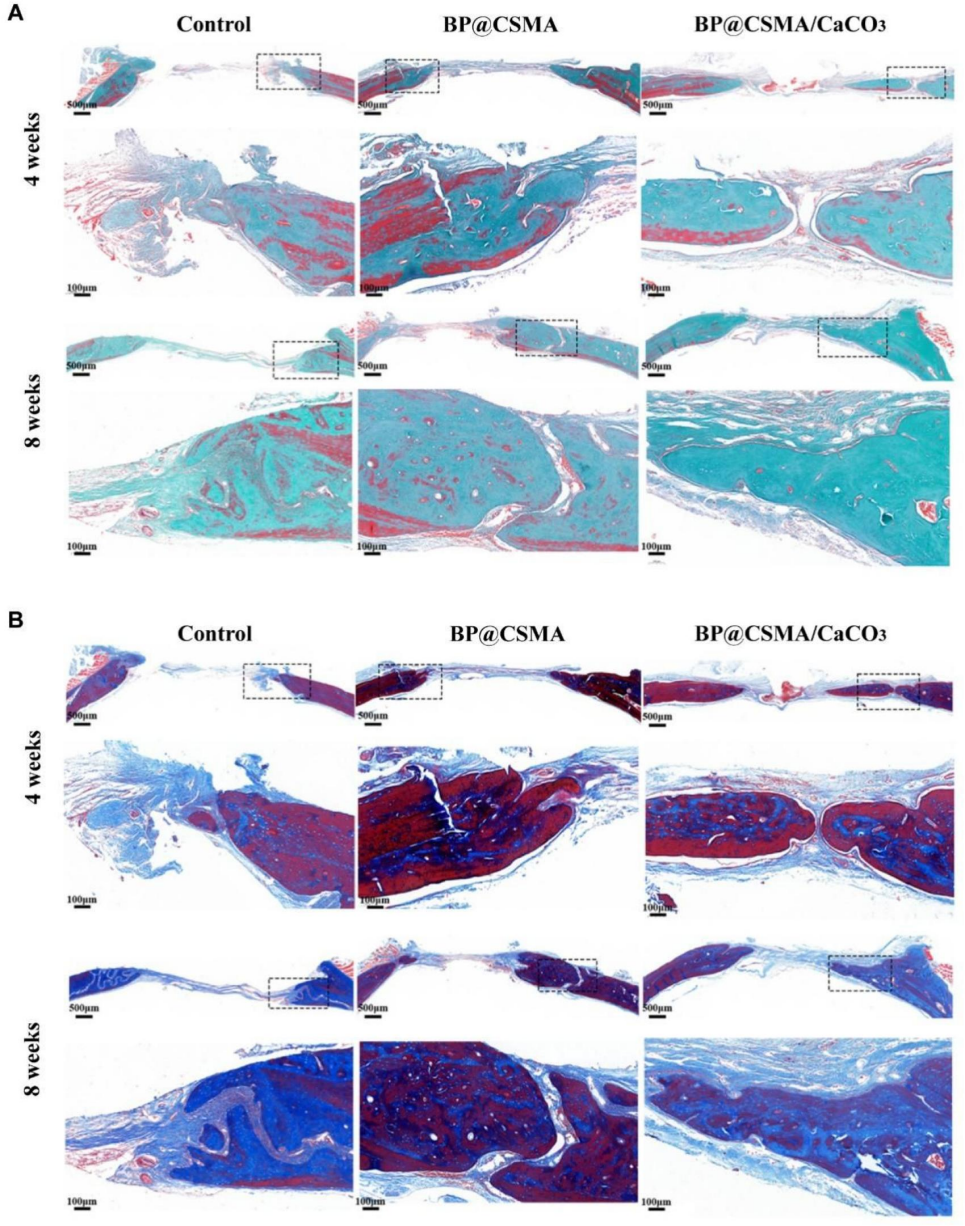
Histological evaluation of newly formed bone after composite hydrogel implantation. (**A**) Representative Goldner’s trichrome staining images of calvarial defects in the control, BP@CSMA, and BP@CSMA/CaCO_3_ groups at 4 and 8 weeks. (**B**) Representative Masson’s trichrome staining images of calvarial defects in the control, BP@CSMA and BP@CSMA/CaCO_3_ groups at 4 and 8 weeks. *n* = 6.

H&E staining showed no obvious inflammation, tissue necrosis, or rejection reaction in any group (Supplementary Figure S5), indicating good *in vivo* biocompatibility. As illustrated in Figure 10A, newly formed bone was seldom observed in the control group; the defect area was mostly occupied by fibrous tissue at 4 weeks. In contrast, BP@CSMA/CaCO_3_ exhibited active osteogenesis at the host bone margins, with osteoid tissue distributed along the defect region. At 8 weeks, osteoid gradually transitioned into mineralized bone (green) in all groups. Specifically, greater bone thickness with a higher degree of mineralization was observed in BP@CSMA/CaCO_3_ group, indicating its superior bone formation ability.

A similar osteogenic trend was observed using Masson staining (Figure 10B). At 4 weeks, only sparse and disorganized collagen fibers were observed in control and BP@CSMA groups, while BP@CSMA/CaCO_3_ displayed more abundant collagen deposition and early mineralized bone formation. By 8 weeks, collagen fibers gradually became organized as time progressed. BP@CSMA/CaCO_3_ exhibited a highly ordered and layered arrangement of collagen fibers.

Nevertheless, several limitations should be acknowledged. The mechanistic investigation in this study remained preliminary, which required further in-depth pathway verification in our future work. Additionally, although BP@CSMA/CaCO_3_ exhibited superior bone regeneration compared with CSMA and BP@CSMA, the respective *in vivo* contributions of CaCO_3_ mineralization and BP incorporation could not be fully resolved since the CSMA/CaCO_3_-only group was not included. In addition, the agar-based trans-tissue model was only a simplified proof-of-concept system *in vitro*, which could not reproduce the multilayer structure, vascular perfusion or heterogeneous properties of real tissues.

Despite these limitations, the present findings indicated that the BP@CSMA/CaCO_3_ hydrogel might represent a promising candidate for the treatment of bone defects complicated by infection, where simultaneous antibacterial activity and osteogenic support were both required. Its injectable and photo-responsive properties also facilitated localized administration and on-demand postoperative modulation.

## Conclusion

In this study, BP@CSMA/CaCO_3_ was developed as an NIR-responsive multifunctional hydrogel for infected bone defect repair. The incorporation of BP and *in situ*-mineralized CaCO_3_ improved hydrogel hydrophilicity, mechanical strength, degradation stability, and sustained Ca^2+^/Pi release. Under NIR irradiation, the composite hydrogel exhibited strong antibacterial activity against *S. aureus* and *E. coli*, mainly through photothermal heating and ROS generation. Meanwhile, BP@CSMA/CaCO_3_ promoted BMSC adhesion, proliferation and osteogenic differentiation by increasing ALP activity and upregulating OPN and OCN. RNA sequencing analysis revealed the involvement of cytoskeletal organization and immune-related pathways in the enhanced osteogenic process. The calvarial defect model confirmed its superior *in vivo* bone regeneration ability. These findings indicated that BP@CSMA/CaCO_3_ provided a promising platform combining antibacterial protection and osteogenic support for infected bone defect repair.

## Supplementary Material

rbag128_Supplementary_Data
