# Supplementary material for: Black phosphorus-loaded mineralized UV-responsive chitosan hydrogel for enhanced osteogenesis and antibacterial activity
Source: Regen Biomater. 2026 Jun 17;13:rbag128. doi: 10.1093/rb/rbag128 (PMC13363252; doi:10.1093/rb/rbag128)
Supplement: rbag128_Supplementary_Data [file rbag128_supplementary_data.zip › 27-Jun-2026_122454_BP@CSMA-CaCO3_proof.docx]

**Black phosphorus-loaded mineralized UV-responsive chitosan hydrogel for enhanced osteogenesis and antibacterial activity**

Xiaoying Xu^1,†^, Xiaofeng Yang^2,†^, Tiantian Gao^1^, Jinjin Zhu^3^, Chao He^2^, Tuwei Jin^1^, Jiuzhou Dong^2^, In-seop Lee^4^, Cen Chen^1,*^, Fan Liu^2,*^

^1^ College of Life Sciences and Medicine, Zhejiang Sci-Tech University, Hangzhou, 310018, PR China

^2^ Department of Orthodontics, School of Stomatology, China Medical University, Shenyang, 110002, PR China

^3^ Department of Orthopaedic Surgery, Sir Run Run Shaw Hospital, Zhejiang University School of Medicine & Zhejiang Key Laboratory of Mechanism Research and Precision Repair of Orthopedic Trauma and Aging Diseases, Hangzhou, 310018, PR China

^4^ Institute of Human Materials, Suwon, 16514, Republic of Korea

^†^ These authors contributed equally to this work

Co-corresponding authors:

Cen Chen

Address: College of Life Sciences and Medicine, Zhejiang Sci-Tech University, No 928, 2^nd^ street, Xiasha High Education Area, Hangzhou 310018, PR China

Email: chencen@zstu.edu.cn

Fan Liu

Address: Department of Orthodontics, School of Stomatology, China Medical University, No. 117, North Nanjing Street, Heping District, Shenyang, 110002, PR China

Email: fanliu@cmu.edu.cn

**Abstract**

Favorable osteogenesis and effective antibacterial activity are two essential requirements for ideal scaffolds in bone tissue engineering, particularly for infected bone defects. Herein, we developed a novel hydrogel system based on mineralized ultraviolet (UV)-responsive methacrylated chitosan hydrogel (CSMA) integrated with black phosphorus nanosheets (BP) exhibiting photothermal effect. BP was incorporated into CSMA, and subsequently subjected to *in situ* biomineralization to form a BP-loaded mineralized hydrogel (BP@CSMA/CaCO_3_). The physicochemical and biological properties of BP@CSMA/CaCO_3_ were systematically investigated *in vitro*. BP incorporation and CaCO₃ mineralization improved hydrogel stability and enabled sustained Ca²⁺ and phosphorus ion release, thereby supporting an osteogenesis-favorable microenvironment. Under high-intensity near-infrared (NIR) irradiation, BP-containing hydrogels exhibited strong antibacterial activity, which was supported by both photothermal heating and singlet oxygen generation. Under mild NIR stimulation, BP@CSMA/CaCO₃ promoted BMSC proliferation and osteogenic differentiation, with increased ALP activity and osteogenic gene expression. Transcriptomic analysis further suggested the involvement of cytoskeletal and immune-related regulation in the enhanced osteogenic response. The application of BP@CSMA/CaCO_3_ in a calvarial defect model *(in vivo*) indicated its superior bone formation ability. These findings suggested that BP@CSMA/CaCO_3_ integrated antibacterial activity, ion-mediated osteogenic support and NIR-responsive regulation, which had great potential for bone regeneration.

**Key words**

Chitosan, mineralization, osteogenesis, black phosphorus nanosheets, antibacterial, NIR irradiation

**1. Introduction**

With population aging and increasing prevalence of unhealthy lifestyles, over 200 million people worldwide are affected by skeletal system disorders, including osteoarthritis, osteoporosis, osteonecrosis or fracture, and this number continues to rise at an annual rate of 10% [1]. Clinically, bone defects require surgical intervention using bone graft materials, such as autografts, allografts, and xenografts, etc [2]. It is estimated the global market shares of bone substitute materials will reach USD 56 billion by 2034. Given the limited source of autografts, the development and application of artificial bone substitutes have become increasingly crucial in orthopedic therapy [3].

Hydrogels are widely utilized in biomedical applications owing to their three-dimensional network structure, which facilitates nutrient transport and metabolic waste exchange [4]. In bone tissue engineering, hydrogels generally demonstrate good biocompatibility but often lack osteoconductive and osteoinductive capabilities [5]. Chitosan (CS), a natural polysaccharide derived from the deacetylation of chitin, has been extensively investigated for bone regeneration due to its low toxicity, excellent biocompatibility, and biodegradability [6-9]. Methacrylated chitosan (CSMA) is synthesized through the reaction of amino groups in CS with methacrylic anhydride (MA), introducing photo-crosslinkable moieties that enable a sol-to-gel transition under UV irradiation [10]. This property allows CSMA to be injected and gelled *in situ*, adapting to bone defects of various geometries [11]. Despite these advantages, the limited intrinsic osteogenic properties of CSMA still restrict its broader application in bone regeneration [12]. To overcome this limitation, we previously incorporated calcium carbonate (CaCO₃) particles into CSMA via *in situ* mineralization [13]. The resulting mineralized hydrogel enabled sustained release of Ca²⁺, which effectively enhanced the osteogenic differentiation of bone mesenchymal stem cells (BMSCs) [14].

However, the promotion of osteogenesis alone is insufficient, as implant-related infection, occurring in 0.7-4.2% of orthopedic surgeries, remains another major obstacle to successful bone repair [15]. A competitive relationship exists between the bacterial adhesion and host cell attachment on the surface of bone substitutes after orthopedic surgery, particularly during the critical early period (4-6 h) after implantation [16,17]. Bacterial colonization is determined by the initial bacterial load, while the adhesion of cells with prominent osteogenic capability is essential for new bone formation, indicating the simultaneous or sequential antibacterial and osteogenic effects are necessary for bone substitutes [18-21]. Consequently, there is urgent need to develop modification strategies that impart multifunctional bioactivity to implanted hydrogels.

These challenges highlight the need for biomaterials that can provide antibacterial protection while supporting subsequent bone regeneration [22]. Black phosphorus nanosheets (BP) are representative two-dimensional photothermal nanomaterials composed of layered phosphorus atoms [23]. Their layer-dependent bandgap and broad near-infrared (NIR) absorption endow BP with efficient photothermal and photodynamic properties [24]. BP has attracted growing interest in biomedical applications owing to its non-invasiveness, remote controllability, biodegradability, and biocompatibility [25]. BP can exert different biological effects by adjusting NIR irradiation conditions. Specifically, high-intensity NIR irradiation induces antibacterial activity through photothermal heating and reactive oxygen species (ROS) generation, whereas mild thermal stimulation can promote osteogenesis [26-28]. Furthermore, the released phosphorus ions during BP degradation are essential for new bone formation [29]. Based on these considerations, BP was introduced into the mineralized CSMA system to construct a multifunctional hydrogel with both photothermal antibacterial activity and osteogenic potential [30,31]. However, its inherent instability remains a major obstacle for future clinical translation [32].

To address the limited osteogenic and antibacterial capabilities of CSMA hydrogels [33], we designed a novel mineralized UV-responsive hydrogel by integrating BP nanosheets with an *in situ* mineralized CSMA matrix (BP@CSMA/CaCO₃). The selection of BP and CSMA mineralization was based on their complementary functional properties. BP was introduced as the NIR-responsive component due to its photothermal behaviors, which enabled switchable antibacterial and osteogenic effects, and phosphorus release pattern during degradation [34]. Meanwhile, CSMA mineralization was introduced to provide sustained Ca²⁺ release and a biomimetic microenvironment favorable for osteogenesis [35]. We hypothesized that the integration of BP and mineralized CSMA matrix would establish a synergistically regulated microenvironment, in which photothermal stimulation and ionic cues cooperatively enhance antibacterial efficacy and osteogenic differentiation in a controllable manner.

Thus, the physicochemical properties of the composite hydrogels, including surface morphology, chemical composition, hydrophilicity, surface roughness, swelling ratio, mechanical properties, degradation profile and ions release kinetics were systematically investigated. Their photothermal behavior and singlet oxygen generation were further examined to clarify the NIR-responsive antibacterial mechanism. *In vitro* antibacterial activity was assessed using *Staphylococcus aureus* (*S. aureus*) and *Escherichia coli* (*E. coli*), while cytocompatibility, cell adhesion, proliferation, and osteogenic differentiation of BMSCs were evaluated to determine their biological performance. Furthermore, the underlying mechanisms were explored using RNA sequencing analysis. A rat calvarial defect model was used to evaluate the *in vivo* bone formation ability of BP@CSMA/CaCO₃.

**2. Materials and methods**

2.1 Materials

Chitosan powder (C766420) and calcium chloride (4 M, C915443) were obtained from Shanghai Macklin Biochemical Technology. Methacrylic anhydride (M102519), photoinitiator I2959, and sodium bicarbonate (S432111) were sourced from Aladdin Scientific Inc. Black phosphorus (100 μg/mL, 7723-14-0) was supplied by Hefei Keliao New Material Technology. Calcium (E-BC-K103-M) and Phosphorus (E-BC-K245-M) Colorimetric Assay Kits were obtained from Elabscience. Ammonium carbonate (10001418) came from Sinopharm Chemical Reagent. Singlet Oxygen Sensor Green (SOSG) was obtained from Meilunbio. *Staphylococcus aureus* and *Escherichia coli* were provided by the China General Microbiological Culture Collection Center. Cell culture media, including Growth Medium (C11995500BT) and BMSCs Basal Medium (BLDM-03011), were purchased from Cyagen Biosciences. The following kits and reagents were acquired from Beyotime Biotechnology: CCK-8 (C0039), Calcein-AM/PI Double Stain Kit (C2015S), DAPI (C1006), ALP Assay Kit (P0321S), and BCA Protein Assay Kit (P0010S). Rhodamine-phalloidin (40734ES75) was from Yeasen Biotechnology. ALP Staining Kit (C3250S) was procured from Solarbio. TRIzol reagent (15596026CN) was obtained from Thermo Fisher Scientific. Isoflurane (R510-22-10) was purchased from Shenzhen Ruiwode Life Technology. H&E (G1076), Goldner's Trichrome (G1064), and Masson's Trichrome (G1006) staining kits were acquired from Wuhan Servicebio Technology.

2.2 Synthesis of UV-responsive chitosan (CSMA)

Based on preliminary optimization, a 3% (w/v) CSMA solution was prepared for this study. Briefly, chitosan powder was dissolved into 100 mL 1% (v/v) acetic acid solution to obtain a 3% (w/v) chitosan solution. Methacrylic anhydride solution was then added dropwise into the well-dissolved chitosan solution under constant stirring. The reaction was carried out at 60°C for 12 h, after which the mixture was adjusted to pH = 7 using NaHCO₃ to terminate the reaction. The resulting product was dialyzed for three days to obtain the purified CSMA solution (Figure 1A).

2.3 Preparation of BP-loaded CSMA hydrogels

200 μL of CSMA solution was mixed with 0.2 wt.% photoinitiators and crosslinked by UV irradiation (GGY250, Wuxi Changya Lighting Co., Ltd, Wuxi, China) for 3 min to form CSMA hydrogel. BP (100 μg/mL) was added to CSMA solution containing I2959, stirred for 2 h, and subsequently crosslinked via UV irradiation for 3 min, known as BP@CSMA (Figure 1B). The BP@CSMA/CaCO_3_ hydrogel was synthesized through *in situ* mineralization of BP@CSMA hydrogel, according to our previous research. Briefly, CaCl_2_ solution (4 mol/L) was added into BP@CSMA mixture to provide Ca^2+^ ions, after which the solution was irradiated for 3 min to obtain the CSMA/CaCl₂ precursor. Mineralization of CSMA/CaCl_2_ proceeded via CO_2_ diffusion, which was decomposed by (NH_4_)_2_CO_3._ The CO_2_ was gradually diffused into BP@CSMA/CaCl_2_ matrix, where it reacted with Ca²⁺ ions to form homogeneously dispersed CaCO₃ within the hydrogel, resulting in the final BP@CSMA/CaCO₃ composite (Figure 1C).

Freeze-dried samples were used for SEM observation, mechanical and degradation tests, and osteogenesis evaluation and *in vivo* bone formation assessment. Hydrated gel samples were used for photothermal performance, antibacterial efficacy, cytotoxicity and cell proliferation assays.

**
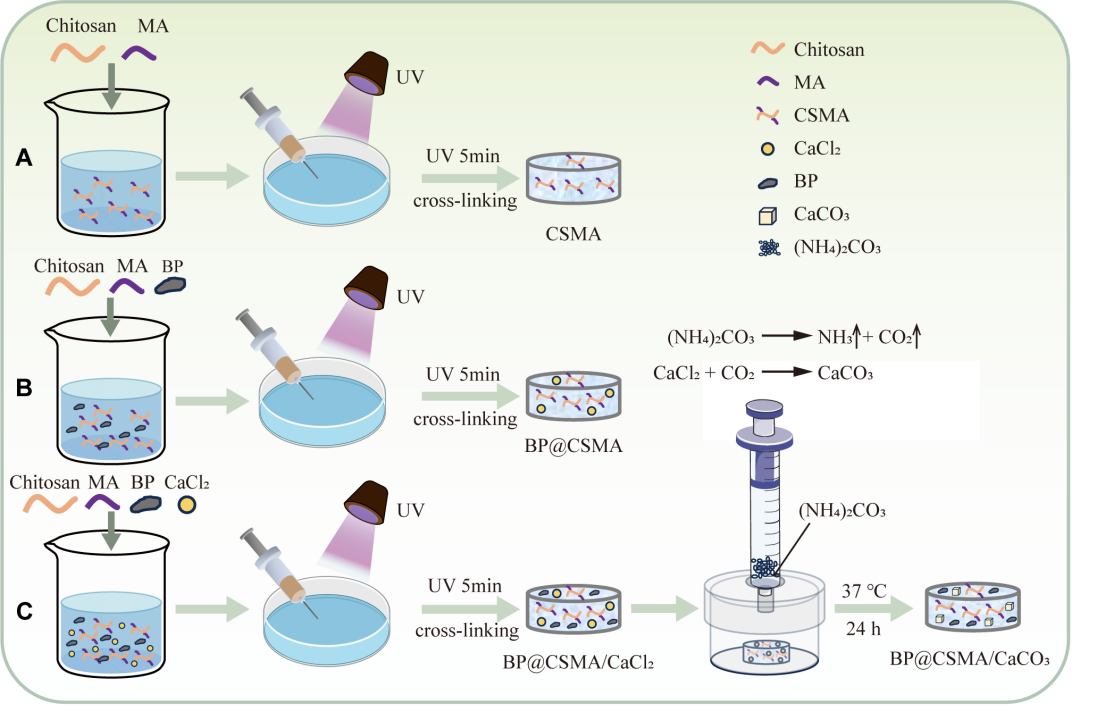
**

Figure 1. Schematic illustration of BP@CSMA/CaCO₃ hydrogel preparation.

2.4 Characterization of the composite hydrogels

2.4.1 Microstructure of BP and composite hydrogels

The morphology of BP was observed by transmission electron microscopy (TEM; JEM-2100, JEOL Ltd., Tokyo, Japan). The microstructure and elemental distribution of lyophilized BP@CSMA and BP@CSMA/CaCO₃ hydrogels were characterized by field-emission scanning electron microscopy equipped with energy-dispersive X-ray spectroscopy (FE-SEM/EDS; MERLIN, Carl Zeiss, Oberkochen, Germany). Prior to imaging, samples were sputter-coated with gold for 90 s and imaged at an acceleration voltage of 3 kV.

2.4.2 Chemical composition

The chemical composition of the samples was characterized by X-ray diffraction (XRD; PW1700, Philips, the Netherlands) and attenuated total reflectance-Fourier transform infrared spectroscopy (ATR-FTIR; Vertex, Bruker, USA). XRD measurements were performed using Cu-Kα radiation at 40 kV and 35 mA, with a scanning rate of 2° min⁻¹ over the 2θ range of 10° to 80°. ATR-FTIR spectra were collected in the wavenumber range of 675-4000 cm^-^¹ at a resolution of 2 cm^-^¹.

2.4.3 Surface roughness

Surface roughness of freeze-dried samples was assessed by using a laser scanning confocal microscope (VK-X100 series, Keyence Corporation, Osaka, Japan). In brief, the surface topography was scanned point by point using the system, and the corresponding roughness parameters were derived from the acquired height data.

2.4.4 Wettability

The surface wettability of the hydrogels was characterized by water contact angle (WCA) measurements using a contact angle goniometer (DSA100s, Krüss GmbH, Hamburg, Germany). 2 μL droplet of deionized water was gently deposited onto the hydrogel surface, and the static contact angle was automatically determined through image analysis of the droplet profile.

2.4.5 Swelling ability

The weight of the lyophilized hydrogel was recorded as W_0_. The hydrogel was immersed in phosphate-buffered saline (PBS) at 37°C. At predetermined time intervals, the swollen hydrogel was removed, gently blotted to remove excess surface water, and weighed to obtain the wet weight (Wₜ). The water absorption capacity (Wₐ) was calculated using the following formula [36]:

$$W_{a}=\frac{W_{t}-W_{0}}{W_{0}}\times100\%$$

2.4.6 Mechanical properties

Compression tests were carried out using a universal testing machine (Instron 5967). Lyophilized samples were compressed at a constant rate of 1 mm/min, and the corresponding stress-strain curves were recorded. The compressive modulus was determined from the slope of the stress-strain curve.

2.4.7 Degradation behavior

The *in vitro* degradation behavior of CSMA, BP@CSMA and BP@CSMA/CaCO₃ hydrogels was evaluated by a gravimetric method. Briefly, lyophilized hydrogel samples were weighed as the initial dry weight (W₀) and immersed in 5 mL PBS containing 0.25 mg/mL lysozyme. The samples were incubated at 37°C and 100 r/min, and the medium was replaced every 2 days. At predetermined time points (1, 2, 3, 4, 5, 6, 7, 8, 9, 10, 14, and 21 days), samples were collected, gently rinsed with deionized water, lyophilized to constant weight, and weighed as Wₓ. The residual weight ratio (Rr) was calculated as:

$$Rr=\frac{Wx-W_{0}}{W_{0}}\times100\%$$

2.5 Photothermal properties of the composite hydrogels

The photothermal properties of BP@CSMA and BP@CSMA/CaCO₃ hydrogels were evaluated under 808 nm NIR laser irradiation (HW808AD2000-34F, Shenzhen Fuzhe Fulei Technology Co., Ltd., China) at power densities of 1 or 2 W/cm² for 5 min. For photothermal measurement, hydrated hydrogel samples were placed under non-immersed conditions, and the temperature was recorded directly from the exposed hydrogel surface using an infrared thermal camera (Research-N1, Hangzhou Meisheng Infrared Electro-Optic Technology Co., Ltd., China) at 1-min intervals during irradiation. Therefore, the measured temperature primarily reflected the surface photothermal response of the hydrogel samples rather than the temperature of a surrounding solution.

The photothermal stability of both hydrogels was evaluated through repeated heating-cooling cycles, consisting of 5 min of NIR irradiation at 2 W/cm² followed by 3 min of natural cooling. The temperature profiles were recorded over three consecutive cycles to assess the durability of the photothermal response.

To provide a preliminary assessment of trans-tissue photothermal behavior, a 5-mm-thick agar block was used as a simplified tissue-mimicking barrier. The agar block was placed over the hydrogel samples, which were then irradiated with the 808 nm NIR laser at 2 W/cm² for 5 min. The temperatures of both the upper agar surface and the hydrogel-agar interface were recorded to evaluate heat transfer across the hydrated agar layer.

2.6 Ion release assay

The ion release behavior of the hydrogels was evaluated by measuring calcium (Ca²⁺) and inorganic phosphate (Pi) concentrations in the extracts. For Ca²⁺ release, extracts from BP@CSMA/CaCO₃ hydrogels were collected on days 1, 3, 5, 7, 9, 11, and 13. Ca²⁺ concentration was quantified using a calcium colorimetric assay kit (E-BC-K103-M, Elabscience, Wuhan, China) at 610 nm. Ca²⁺ concentration was calculated based on the standard curve using the following equation:

$$Ca^{2}⁺=\frac{\Delta A₆₁₀ - b}{a}\times f$$

where ΔA₆₁₀ represented the difference between sample and blank absorbance, a and b were the slope and intercept of the Ca²⁺ standard curve, and f was the dilution factor.

For Pi release, extracts from BP@CSMA, BP@CSMA + NIR, BP@CSMA/CaCO₃ and BP@CSMA/CaCO₃ + NIR were collected at the same time points. The NIR-treated groups were irradiated with an 808 nm laser at 2 W/cm² for 5 min. Pi concentration was measured using a Phosphorus Colorimetric Assay Kit (E-BC-K245-M, Elabscience, Wuhan, China) at 660 nm. P concentrations were calculated from the corresponding standard curves using the following equation:

$$Pi=\frac{\Delta A₆₆₀ - b}{a}\times5\times f$$

where ΔA₆₆₀ represented the blank-corrected absorbance, a and b were the slope and intercept of the Pi standard curve, respectively, 5 was the kit conversion factor, and f was the dilution factor.

2.7 *In vitro* antibacterial assessments of the composite hydrogels

2.7.1 Antibacterial rates

The antibacterial activity of the composite hydrogels was evaluated using *Staphylococcus aureus* (*S. aureus*) and *Escherichia coli* (*E. coli*). The experiment included four groups: control (tissue-culture polystyrene, TCP), CSMA, BP@CSMA, and BP@CSMA/CaCO₃, with or without NIR laser irradiation (2.0 W/cm², 5 min). After sterilization at 115°C for 30 min, each sample was incubated with 500 μL bacterial suspension (1 × 10⁶ CFU/mL) at 37°C for 3 h. Adherent bacteria were detached by ultrasonication for 2 min, and 100 μL of the collected suspension was spread onto agar plates and incubated at 37°C for 24 h. The number of bacterial colonies was counted according to ISO 22196, and the antibacterial rate was calculated using the following formula [43]:

$$\text{A}\text{ntibacterial rate }(\%)=\frac{\text{CFU of control group}-\text{CFU of experimental group}}{\text{CFU of control group}}\times100\%$$

After NIR irradiation, the collected bacterial suspension was fixed with 2.5% glutaraldehyde for 2 h, washed with PBS, and dehydrated through a graded ethanol series for 30 min at each concentration. The bacterial morphology was then observed by SEM.

2.7.2 Singlet oxygen generation

Singlet oxygen generation was quantified using Singlet Oxygen Sensor Green (SOSG, Meilunbio). Hydrogels from five groups, including CSMA, BP@CSMA, BP@CSMA + NIR, BP@CSMA/CaCO₃ and BP@CSMA/CaCO₃ + NIR, were incubated with SOSG working solution. The NIR-treated groups were exposed to an 808 nm laser at 2 W/cm². At predetermined time points, the supernatant was collected, and fluorescence intensity was measured at an excitation wavelength of 504 nm and an emission wavelength of 525 nm to quantify singlet oxygen generation.

2.8 *In vitro* biocompatibility of the composite hydrogels

2.8.1 The cytotoxicity

The cytotoxicity of BP@CSMA and BP@CSMA/CaCO_3_ was evaluated based on the international standard (ISO10993-5) [38]. Briefly, hydrogel extracts were collected after incubating each sample in growth medium at 37°C for 3 days. BMSCs were first cultured on tissue-culture polystyrene (TCP) overnight, after which the culture medium was replaced with the corresponding extract medium. BMSCs cultured on TCP in fresh growth medium served as the negative control, representing the baseline culture condition and defined as 100% cell viability. After 24 and 48 h of exposure, cell viability was assessed using the CCK-8 assay by measuring absorbance at 450 nm. Live/dead staining was further performed using a calcein-AM/PI double-staining kit. At 24 and 48 h, cells were imaged under a confocal laser scanning microscope (CLSM, IX81-FV1000, Olympus Corporation, Tokyo, Japan).

Furthermore, to evaluate the influence of NIR irradiation on cell viability, BMSCs were seeded on BP@CSMA and BP@CSMA/CaCO₃ hydrogels and cultured for 24 h, with or without NIR laser irradiation (1.0 W/cm², 1 min). Cell viability was assessed using the CCK-8 assay at 24 h and 48 h post-irradiation. The relative cell viability was defined as the ratio of the OD value in irradiated samples to that in non-irradiated controls.

2.8.2 Cell proliferation rate

BMSCs were seeded onto the samples (CSMA, BP@CSMA and BP@CSMA/CaCO_3)_ at a density of 2 × 10⁴ cells per well. Cell proliferation was assessed on days 1, 3, 5, and 7 using the CCK-8 assay. NIR irradiation (1W/cm^2^, 1 min) was applied to BP@CSMA and BP@CSMA/CaCO_3_ every three days. For cell adhesion and morphology observation, BMSCs were cultured on the samples for 12 h, fixed, and stained with rhodamine-phalloidin and DAPI to visualize F-actin and nuclei, respectively. The stained cells were visualized using a confocal microscope.

2.9 Osteogenesis of the composite hydrogels

The osteogenic differentiation of BMSCs cultured on CSMA, BP@CSMA and BP@CSMA/CaCO₃ hydrogels was evaluated by quantifying ALP activity and the expression of osteogenesis-related genes, including osteocalcin (OCN) and osteopontin (OPN). The complete osteogenic medium consisted of BMSCs basal medium supplemented with 10% fetal bovine serum, 1% antibiotic-antimycotic, 3 mmol/L β-glycerophosphate, 50 μg/mL ascorbic acid, and 1 μmol/L dexamethasone (Dex). The mineralizing medium was defined as the same components but without Dex. In detail, the complete osteogenic medium was used as the positive control, whereas the Dex-free mineralizing medium was used to reduce the influence of strong exogenous osteogenic induction, thereby better revealing the intrinsic osteogenic effects of the hydrogels. β-Glycerophosphate and ascorbic acid were retained to support extracellular matrix maturation and mineral deposition. For photothermal stimulation, BP@CSMA and BP@CSMA/CaCO₃ were exposed to 808 nm NIR irradiation at 1 W/cm² for 1 min once every three days.

2.9.1 ALP activity

ALP activity is a well-established early-stage marker of osteogenic differentiation. Briefly, BMSCs were seeded on the composite hydrogels at a density of 2 × 10⁴ cells/mL and cultured in growth medium until reaching 60-70% confluence, after which the medium was replaced with mineralizing medium. On days 5 and 7, cells were washed with PBS, lysed with RIPA buffer, and centrifuged at 5000 rpm for 10 min. The supernatants were collected for ALP activity measurement using a commercial pNPP-based ALP assay kit. After incubation with pNPP substrate at 37°C for 30 min, the reaction was stopped, and absorbance was measured spectrophotometrically. ALP activity was calculated from a p-nitrophenol standard curve and normalized to total protein content determined by BCA assay.

Meanwhile, ALP staining was performed on BMSCs cultured on each sample according to a standard protocol. On days 5 and 7, cells were fixed with 10% neutral formalin, washed with PBS, and incubated with ALP staining solution in the dark for 20 min. After two additional PBS washes, images were acquired using an inverted microscope (DS-Ri2, Nikon Corporation, Tokyo, Japan) equipped with a color camera.

2.9.2 The expression of osteogenesis-related genes

Quantitative real-time reverse transcription polymerase chain reaction (qRT‑PCR) was performed to analyze the mRNA expression levels of osteopontin (OPN) and osteocalcin (OCN) in BMSCs cultured on the samples on days 7 and 14. Total RNA was extracted using TRIzol reagent, and RNA concentration was measured spectrophotometrically. The primer sequences used for PCR amplification were listed in Table S2. Gene expression levels were quantified using the 2^⁻ΔΔCT^ method.

2.10 RNA sequencing analysis

BMSCs were cultured on CSMA and BP@CSMA/CaCO₃ hydrogels for 7 days. The BP@CSMA/CaCO₃ group was exposed to 808 nm NIR irradiation at 1 W/cm² for 1 min once every three days. Total RNA was extracted using TRIzol reagent and subjected to RNA sequencing by Aksomics Biotech Co., Ltd. (Shanghai, China). Differentially expressed genes were identified using an adjusted *p* value < 0.05 and an absolute fold change > 2 as the cutoff criteria.

2.11 *In vivo* bone formation ability

2.11.1 Surgical procedures

Male Sprague-Dawley rats (8 weeks old, weighing 200 ± 15 g) were randomly divided into three groups: control (defects implanted with CSMA hydrogel only), BP@CSMA and BP@CSMA/CaCO₃ (n = 6 per group). After induction of anesthesia via isoflurane inhalation, a circular calvarial defect (5 mm in diameter and 1.5 mm in depth) was created using a 5.0 mm trephine drill at 800 rpm under aseptic conditions. The corresponding hydrogel implants were placed into the defects, and the surgical area was rinsed with sterile saline. For BP@CSMA and BP@CSMA/CaCO₃, postoperative photothermal treatment was administered using an 808 nm laser at 1 W/cm² for 3 min at three-day intervals for 2 weeks [39]. At 4 and 8 weeks after implantation, all animals were euthanized for subsequent radiographical and histological analyses.

2.11.2 Micro-CT analysis

Block sections containing the implanted samples and surrounding tissues were harvested and fixed in a 10% neutral formalin solution for 14 days. The samples were then scanned using a micro-CT system (VIVA-40, Scanco Medical AG, Bruttisellen, Switzerland), and the acquired images were reconstructed using TRI/3D-BON software. Morphometric parameters, including bone volume (BV), bone surface (BS) and trabecular thickness (Tb.Th), were quantified to evaluate new bone formation within the region of interest (ROI).

2.11.3 Histological analysis

Tissue blocks were dehydrated through a graded ethanol series, cleared in xylene twice for 1 h each, and embedded in paraffin wax. Serial sections of 40 μm thickness were cut along the coronal plane and subjected to HE staining, Goldner’s trichrome or Masson’s staining for histological observation. For Goldner’s trichrome staining, osteoid tissue was identified by its orange-red coloration, whereas mature bone appeared green. In terms of Masson staining, collagen fibers were highlighted in blue, and soft tissues were stained in red or orange.

2.12 Statistical analysis

All data were depicted as mean ± standard deviation. Statistical significance among groups was determined by one-way ANOVA followed by Tukey's post hoc test. The difference was considered statistically significant at **P* < 0.05 and ***P* < 0.01.

**3. Results and discussion**

3.1 Characterization of BP and composite hydrogels

BP was well dispersed in the solution without obvious aggregation or sedimentation (Figure 2A). TEM image revealed a typical two-dimensional layered morphology with a lateral size of approximately 215 nm (Figure 2B) [40,41]. As shown in Figure 2C, lyophilized CSMA and CSMA/CaCO₃ hydrogels appeared transparent and milky, respectively, whereas BP incorporation resulted in a visibly darker appearance. SEM images showed all samples exhibited interconnected microporous structures (Figure 2D). Compared with CSMA, BP@CSMA displayed smaller pores, which might be related to interactions between BP and the CSMA matrix [42]. BP@CSMA/CaCO₃ exhibited a porous architecture with embedded particulate deposits. These mineral aggregates, being randomly dispersed within the CSMA matrix, were identified as calcite crystals in our previous study [13]. EDS mapping (Figure S1) revealed a homogeneous distribution of carbon (C), nitrogen (N), and oxygen (O) on the surface of the CSMA hydrogel. BP@CSMA exhibited an additional P signal with a content of 2.54%. In the BP@CSMA/CaCO₃ sample, the elemental mapping demonstrated a uniform presence of C, N, O, P, and calcium (Ca). The measured contents of P (2.77%) and Ca (25.85%) further verified the co-existence of BP and CaCO₃ within the CSMA matrix.


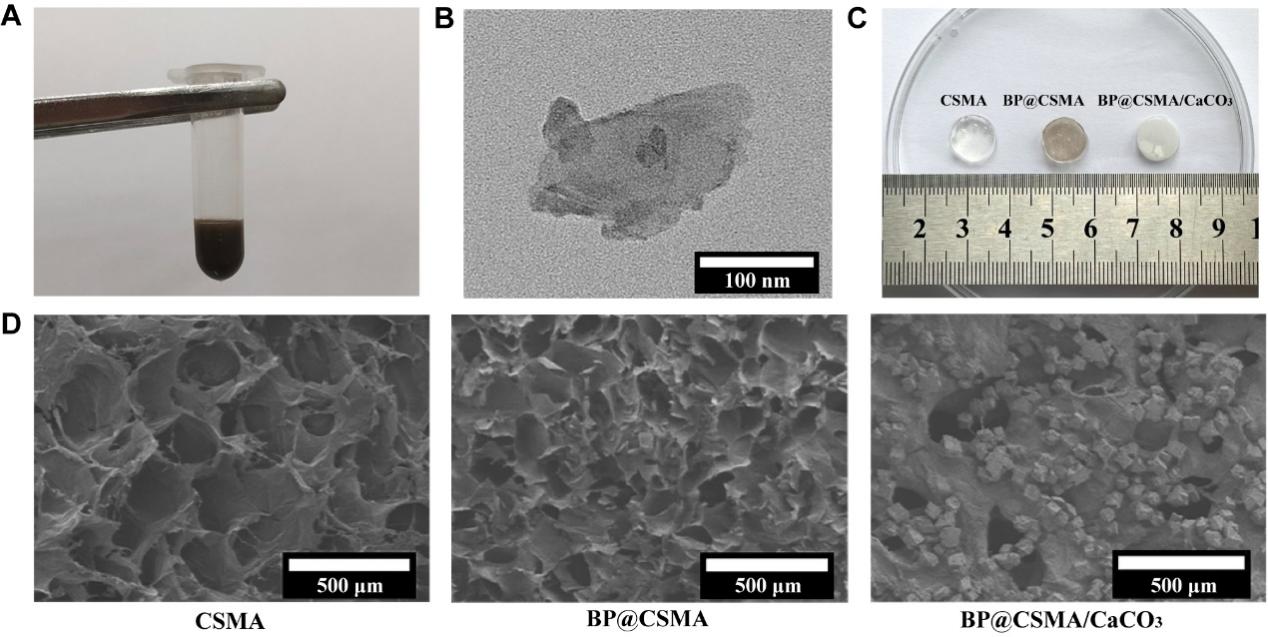


Figure 2. Morphological characteristics of BP and the composite hydrogels. (A) Representative photograph of BP dispersion. (B) Representative image of BP by TEM observation. (C) Representative photographs of lyophilized CSMA, BP@CSMA, and BP@CSMA/CaCO₃ hydrogels. (D) Representative SEM images of CSMA, BP@CSMA and BP@CSMA/CaCO_3_

XRD and ATR-FTIR analyses were performed to verify the phase composition and chemical structure of the CSMA-based hydrogels. As shown in Figure 3A, CSMA exhibited a broad and diffuse peak at approximately 20.3°, indicating its amorphous structure associated with hydrogen bonding and N-acylation within the polymer network [43,44]. In CSMA/CaCO₃, distinct diffraction peaks at 23.5° and 29.7° were assigned to the (012) and (104) planes of calcite, respectively, confirming the successful mineralization of CaCO₃ in the calcite phase [13]. In addition, characteristic BP peaks at 16.9°, 26.9°, and 40.5° were observed in BP-containing samples, indicating that the BP crystal structure was retained within the CSMA hydrogel. ATR-FTIR spectra further confirmed the successful fabrication of BP@CSMA/CaCO₃ (Figure 3B). The characteristic peaks of the CSMA matrix were the amide I (1658 cm⁻¹), amide II (1551 cm⁻¹), and amide III (1319 cm⁻¹) bands [43].Critically, the presence of CaCO₃ was confirmed by the appearance of peaks at 1398 cm⁻¹ (asymmetric stretching of CO₃²-), 827 cm-¹ (out-of-plane deformation of CO₃²⁻), and 717 cm⁻¹ (in-plane deformation of O-C-O) [13]. Furthermore, the BP incorporation was supported by a new peak at 1009 cm⁻¹, which was attributed to the P=O stretching vibration [45].


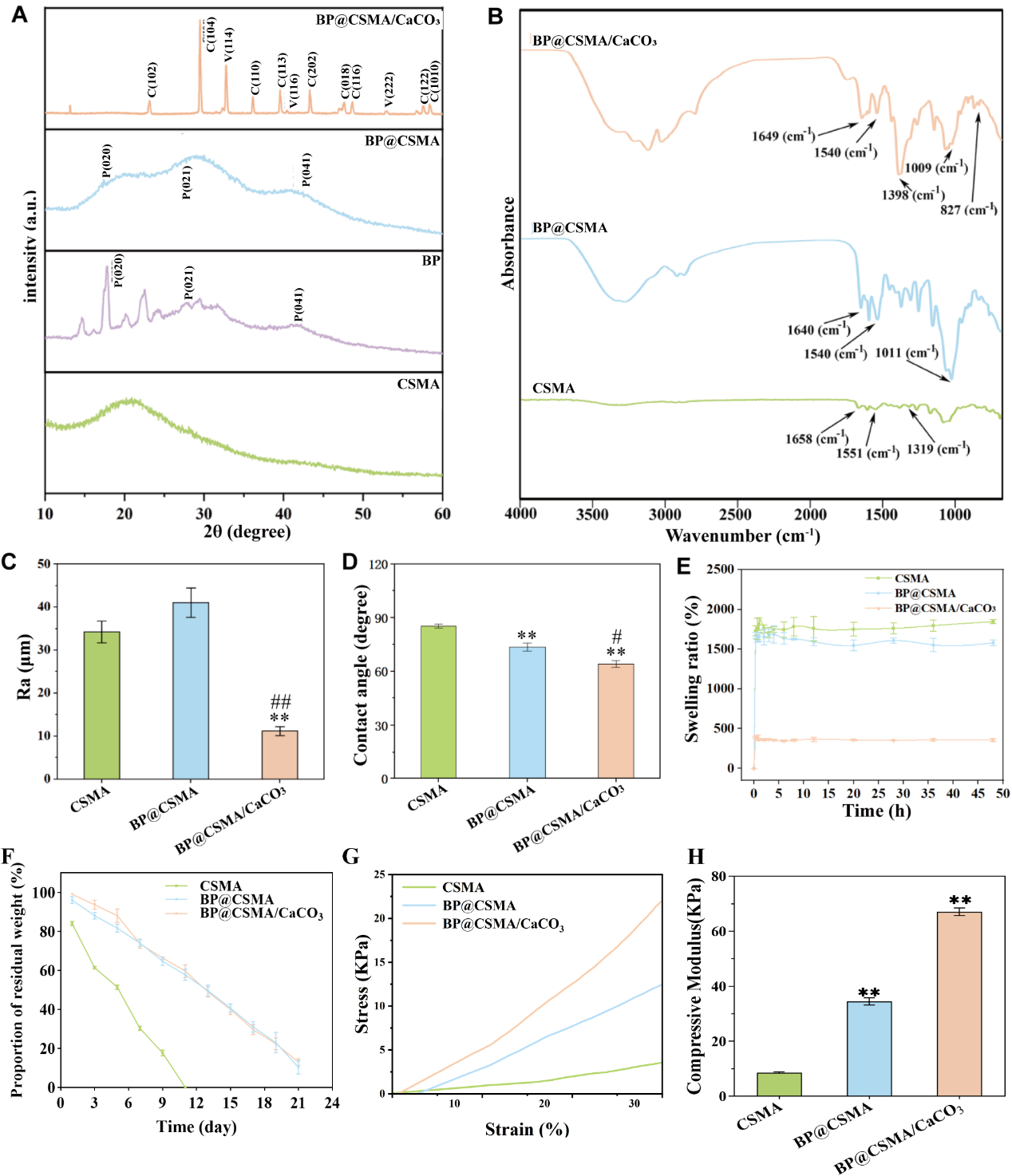


Figure 3. Physicochemical characterization of the composite hydrogels. (A) XRD pattern. (B) AIR- FTIR spectra. (C) Surface roughness. (D) Water contact angle. (E) swelling ratio. (F) *In vitro* degradation behavior. (G) Compressive stress-strain curves. (H) Compressive modulus. Data are presented as mean ± SD, n = 3.

The average surface roughness (Ra) values of CSMA, BP@CSMA and BP@CSMA/CaCO₃ were measured as 34.22 ± 2.55 μm, 41.02 ± 3.43 μm, and 11.19 ± 1.03 μm, respectively (Figure 3C). CaCO₃ mineralization markedly reduced Ra, which might be attributed to mineral deposits filling the porous structure and producing a more homogeneous surface [46]. In contrast, the introduction of BP led to a moderate increase in Ra values, suggesting that the distinct morphology of BP contributed to greater surface texture in the composite hydrogel [47].

BP@CSMA showed enhanced surface wettability compared with CSMA, while BP@CSMA/CaCO₃ exhibited the strongest hydrophilicity (Figure 3D). The initial enhancement in wettability from CSMA to BP@CSMA was primarily governed by an increase in surface roughness, which amplified the intrinsic hydrophilicity of the polymer matrix. The stronger hydrophilicity of BP@CSMA/CaCO₃ might be attributed to the mineralized layer formed during biomineralization, which created a more wettable surface interface.

Adequate swelling is important for hydrogels to maintain a moist microenvironment and support nutrient diffusion [48]. As illustrated in Figure 3E, all samples exhibited rapid swelling within the first 2 h, followed by a slower swelling phase by 50 h. Overall, BP@CSMA/CaCO₃ exhibited significantly lower swelling ratios compared to CSMA and BP@CSMA. In this study, CaCO₃ particles served as a physical filler within the CSMA network, occupying pore spaces and thereby reducing the available volume for water absorption and retention [49,50]. Fthermore, the addition of BP and CaCO₃ effectively reinforced the polymer network, enhancing the cross-linking density and structural integrity, which collectively restricted hydrogel expansion and led to the observed reduction in swelling capacity [51,52].

For degradation behavior, CSMA degraded rapidly and was almost completely degraded within approximately 11 days. In contrast, BP@CSMA and BP@CSMA/CaCO₃ exhibited slower degradation profiles, with approximately 10% and 15% of residual weight remaining at day 21, respectively. The delayed degradation of BP@CSMA might be attributed to the reinforcement of BP within the CSMA network [53,54]. BP acted as physical nanofillers and interacted with CSMA chains, thereby improving network stability. In BP@CSMA/CaCO₃, the mineralized phase might further occupy pores and strengthen the hydrogel matrix [55].

The mechanical properties of CSMA, BP@CSMA and BP@CSMA/CaCO₃ hydrogels were evaluated by compression testing. As shown in Figure 3G, BP@CSMA/CaCO₃ exhibited higher compressive stress than BP@CSMA and CSMA under the same strain. Consistently, the compressive modulus increased from 8.62 ± 0.20 kPa for CSMA to 34.48 ± 1.09 kPa for BP@CSMA, and further to 67.10 ±1.12 kPa for BP@CSMA/CaCO₃ (Figure 3H), indicating progressively enhanced mechanical strength. The improvement in BP@CSMA might be related to the reinforcing role of BP within the CSMA matrix, as BP has been reported to strengthen hydrogel-based constructs by facilitating stress transfer within polymer networks [56]. The introduction of CaCO₃ provided rigid inorganic phases into the hydrogel, further increasing its resistance to deformation, consistent with previous reports on CaCO₃-reinforced hydrogels [57].

Ca²⁺ release from BP@CSMA/CaCO₃ was measured to verify the ion-supplying capacity of the mineralized hydrogel. As shown in Figure S2A, BP@CSMA/CaCO₃ exhibited sustained Ca²⁺ release over time. The cumulative Ca²⁺ concentration increased from 0.49 ± 0.11 mmol/L on day 1 to 2.61 ± 0.13 mmol/L on day 13, indicating a gradual release behavior. This sustained release was likely derived from the progressive dissolution of the mineralized CaCO₃ phase, which might contribute to the formation of a favorable ionic microenvironment for osteogenic differentiation.

As shown in Figure S2B, BP@CSMA exhibited continuous Pi accumulation, reaching approximately 65 mmol/L by day 13, whereas BP@CSMA/CaCO₃ released a lower amount of Pi, approximately 45.3 ± 4.63 mmol/L at the same time point. After NIR irradiation, Pi release was markedly promoted, increasing to approximately 95 mmol/L in BP@CSMA and 61.89 ± 3.67 mmol/L in BP@CSMA/CaCO₃ by day 13. This NIR-enhanced Pi release indicated that photothermal stimulation might accelerate BP degradation and phosphorus diffusion. The lower Pi release from BP@CSMA/CaCO₃ compared with BP@CSMA suggested that CaCO₃ mineralization could modulate the Pi release behavior within the hydrogel matrix.

3.2 Photothermal performance of BP-loaded hydrogels

BP typically exhibits excellent photothermal capacity under NIR irradiation [58]. As shown in Figure 4A, the temperatures of CSMA remained near ambient temperature (∼20°C) under both power densities. In contrast, BP@CSMA and BP@CSMA/CaCO₃ displayed a rapid temperature increase from 20°C to approximately 60°C under 2 W/cm² NIR irradiation, reaching final temperatures of 59.3°C and 56.1°C after 5 min, respectively. Moreover, the temperature increase was power- and time-dependent, suggesting that the photothermal response could be regulated by adjusting NIR parameters. It was noteworthy that CaCO₃ reduced the photothermal conversion efficiency of BP, presumably due to increased light scattering and reflection within the mineral-composite matrix (Figure 4A and 4D) [59]. Additionally, BP@CSMA and BP@CSMA/CaCO_3_ also demonstrated consistent photothermal stability over three on/off heating-cooling cycles (Figure 4B). It should be noted that the photothermal test was based on direct surface temperature measurement of the hydrated hydrogels rather than on bulk solution temperature. Therefore, the results mainly reflected the intrinsic photothermal response of the hydrogel surface. Although released BP in a surrounding liquid phase could theoretically contribute to NIR-induced heating, such an effect was expected to be minimal under the present measurement condition.

To preliminarily evaluate trans-tissue photothermal performance, a 5-mm-thick agar block was used as a simplified tissue-mimicking barrier (Figure 4C). Agar phantoms are widely used in biomedical optical and thermal studies owing to their water-rich composition, reproducibility, and tunable properties [60]. Under this condition, the temperatures measured at the hydrogel surfaces remained close to those obtained without the agar barrier, indicating that agar did not markedly attenuate the photothermal effect of BP@CSMA or BP@CSMA/CaCO₃. This might be attributed to the effective penetration of 808 nm NIR light within the biological window [61]. A clear thermal gradient was observed, with the upper agar surface reaching 40-45°C and the hydrogel-agar interface reaching 55-65°C (Figure 4D), confirming efficient photothermal conversion and heat conduction through the hydrated agar matrix. Importantly, this profile suggested that bactericidal temperatures could be maintained at the implant surface while the superficial tissue-facing side remained at a relatively tolerable temperature (~40°C) [62].


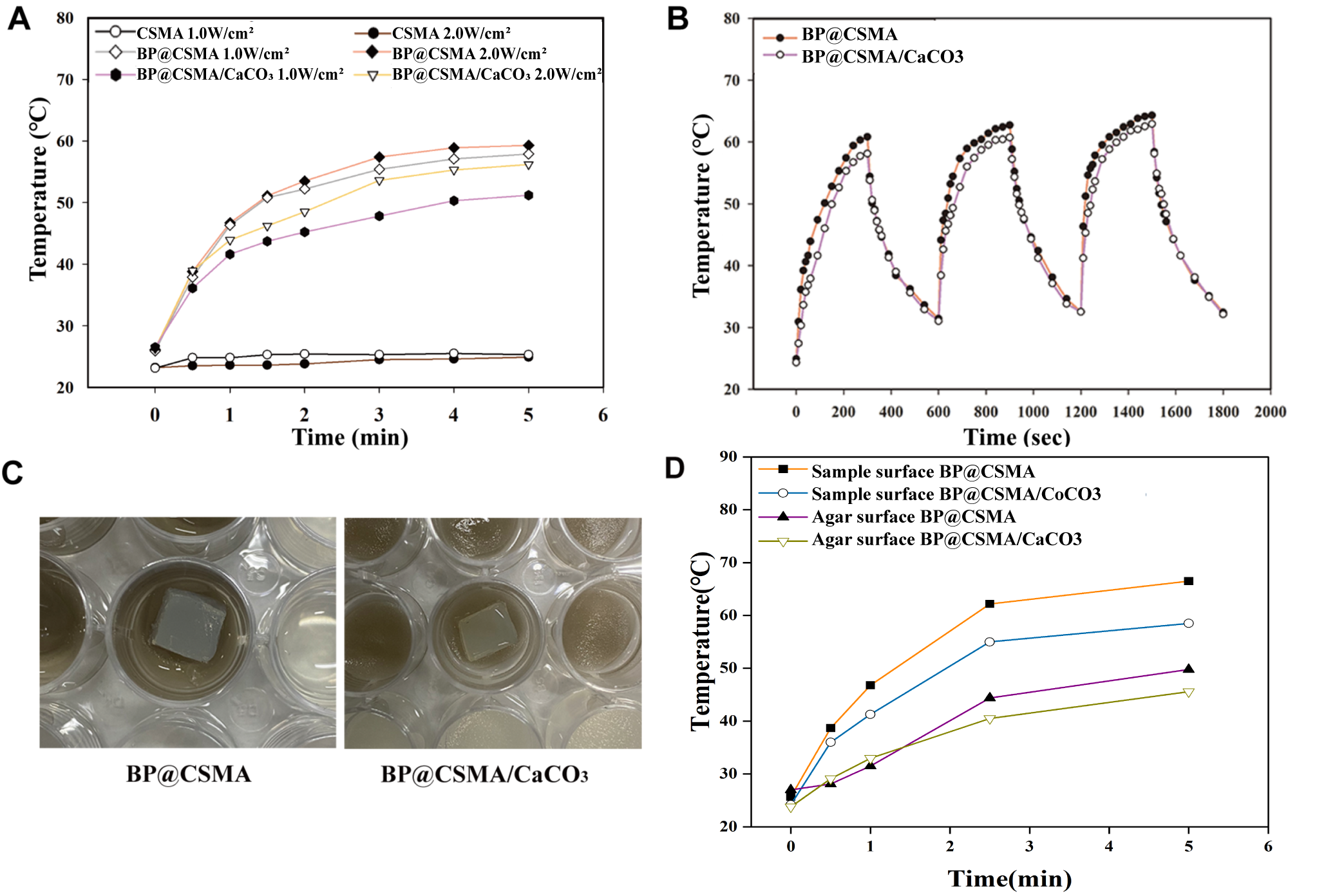


Figure 4. Photothermal performance of the composite hydrogels. (A) Temperature profiles of CSMA, BP@CSMA and BP@CSMA/CaCO₃ under 808 nm NIR irradiation at different laser power densities. (B) Photothermal stability of BP@CSMA and BP@CSMA/CaCO_3_ over three heating-cooling cycles under 808 nm NIR irradiation at 2 W/cm². (C) Representative photographs of BP@CSMA and BP@CSMA/CaCO_3_ covered with a 5-mm-thick agar block as a simplified tissue-mimicking barrier. (D) Temperature profiles of the corresponding samples irradiated through the agar barrier under 808 nm NIR irradiation at 2 W/cm². Data are presented as mean ± SD, n = 3.

3.3 *In vitro* antibacterial performance of the composite hydrogels

As shown in Figure 5A and B, CSMA exhibited moderate antibacterial activity compared with the negative control group, which could be attributed to the inherent antibacterial properties of chitosan. Upon incorporation of BP, the antibacterial rates of BP@CSMA and BP@CSMA/CaCO₃ increased to 66.03% and 59.57%, respectively, even without NIR irradiation. This improvement might be related to the inherent antibacterial activity of BP, including physical disruption of bacterial membranes by its ultrathin layered structure and sharp edges [63]. SEM images further showed distorted and ruptured bacterial membranes after exposure to BP-containing hydrogels (Figure 5C). Under NIR irradiation, the corresponding antibacterial rates reached to 99.28% and 97.84%, exhibiting superior antibacterial effects. This enhanced antibacterial effect was mainly associated with BP-mediated photothermal heating, which rapidly increased the local temperature to approximately 60°C and induced bacterial damage through protein denaturation and membrane disruption [64].

To further clarify the antibacterial mechanism, singlet oxygen generation was quantified using SOSG fluorescence (Figure S3). The CSMA group showed only a weak and stable background signal, whereas BP-containing hydrogels exhibited gradually increased fluorescence intensity, indicating that BP contributed to singlet oxygen generation in composite hydrogels. Upon NIR irradiation, both BP@CSMA and BP@CSMA/CaCO₃ displayed enhanced fluorescence signals, confirming the NIR-responsive ROS-generating capability of BP. Notably, the signal intensity of BP@CSMA was higher than that of BP@CSMA/CaCO₃ under the same condition, which might be attributed to the modulatory effect of CaCO₃ mineralization on BP photoactivity. Therefore, the antibacterial activity of BP-containing hydrogels under NIR irradiation can be attributed to the combined effects of photothermal heating and ROS generation [65,66].


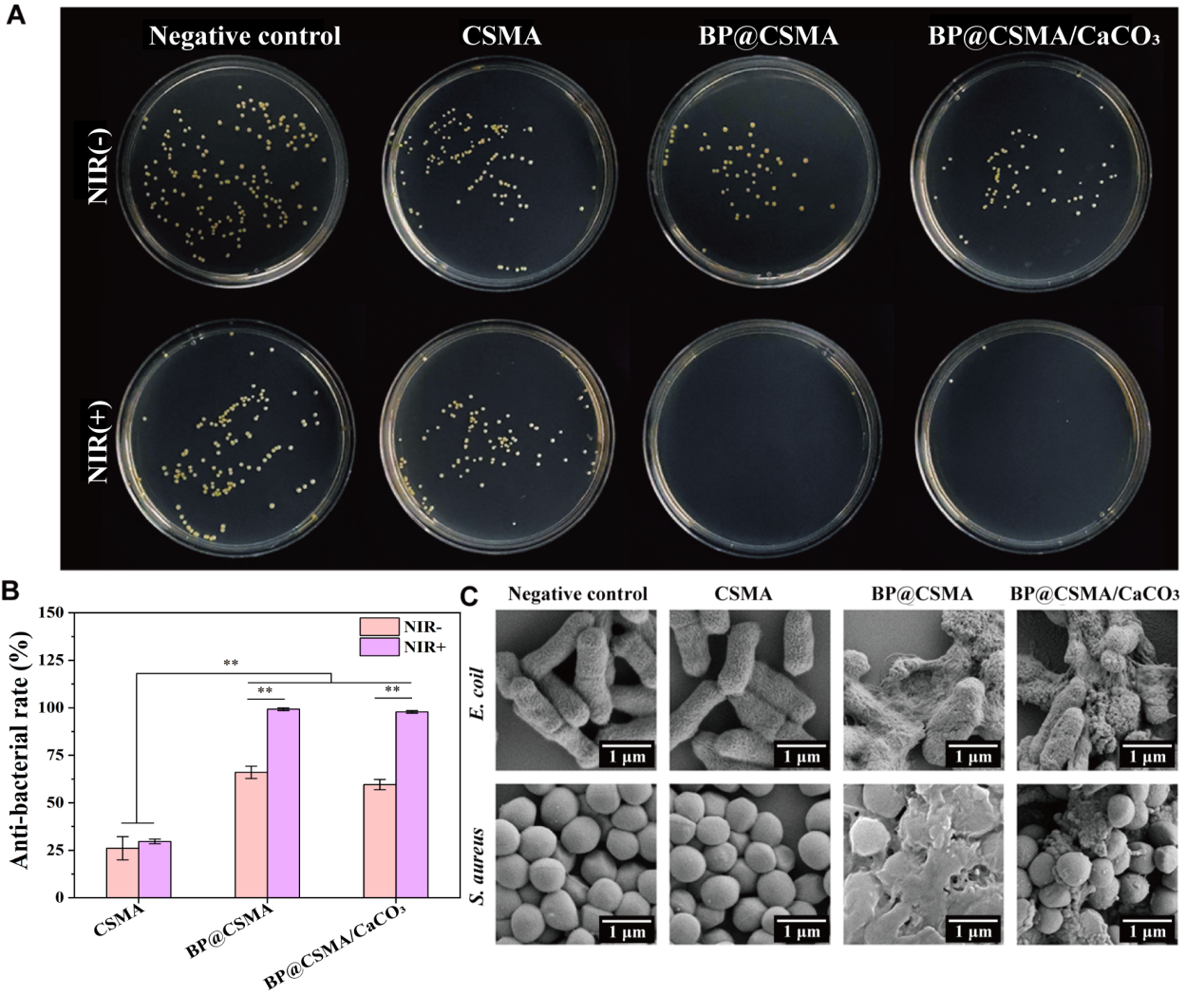


Figure 5. Antibacterial performance of the composite hydrogels. (A) Representative images of *S. aureus* colonies collected from hydrogels with or without NIR irradiation (808 nm, 2 W/cm², 5 min). (B) Quantitative antibacterial rates calculated from colony-forming unit (CFU) counts. (C) Representative SEM images of bacterial morphologies on the composite hydrogels. Data are presented as mean ± SD, n = 3. ^*^*P* < 0.05 and ^**^*P* < 0.01 indicate significant differences between groups.

3.4 *In vitro* biocompatibility of the composite hydrogels

The cytocompatibility of BP@CSMA and BP@CSMA/CaCO_3_ was illustrated in Figure S4. All samples showed cell viabilities above 90%, exceeding the 70% threshold required by ISO 10993-5 (Figure S4A) [67]. Live/dead staining was consistent with the CCK-8 results (Figure S4C). The presence of live cells (green) in all group indicated BP@CSMA and BP@CSMA/CaCO_3_ were non-cytotoxic. To verify the suitability of the NIR parameter used for osteogenic stimulation, the effects of irradiation at 1 W/cm² for 1 min on BMSC viability were evaluated (Figure S4B). An obvious reduction in cell viability was observed at 24 h after irradiation in both BP@CSMA and BP@CSMA/CaCO₃, which might be attributed to the acute photothermal effect and singlet oxygen generated by BP under NIR exposure [68]. However, cell viability recovered significantly by 48 h, particularly in the BP@CSMA/CaCO₃, indicating this irradiation condition did not cause irreversible cytotoxicity. Combined with the enhanced cell proliferation and osteogenic differentiation observed in subsequent assays, these findings supported that 1 W/cm² for 1 min represented an appropriate mild photothermal stimulation parameter for promoting osteogenesis while maintaining acceptable cytocompatibility, which was in line with previous studies [69,70].

The initial adhesion and subsequent proliferation of cells at the material-tissue interface are critical for the early stages of bone regeneration. As shown in Figure 6B, BMSCs displayed characteristic cytoskeletal morphology and were evenly distributed across all hydrogel surfaces. Notably, cells cultured on BP@CSMA/CaCO₃ exhibited more developed actin filaments with extensive filopodia extensions. The formation of this interconnected cellular network demonstrated favorable adhesion and effective spreading on the composite hydrogel surface.

Since NIR irradiation was confirmed to have negligible side effects on cell viability (Figure S4B), the photothermal treatment (1 W/cm², 1 min) was applied to all groups at three-days intervals. Over a 7-days culture period, all groups exhibited time-dependent increases (Figure 6A). On day 1, both BP-loaded hydrogels exhibited significantly higher proliferation rates than the CSMA group. By day 3, BP@CSMA showed relatively low proliferation rate, while BP@CSMA/CaCO₃ maintained a consistent increase, exhibiting significantly higher metabolic activity than both BP@CSMA and CSMA. Since day 5, BP@CSMA/CaCO_3_ and CSMA groups exhibited similar proliferation rates, both significantly exceeding that of BP@CSMA. Notably, on day 7, BP@CSMA/CaCO₃ achieved the highest proliferation level, reaching approximately 1.7-fold that of the BP@CSMA group. The relatively lower proliferation observed in the BP@CSMA might be mainly related to its higher photothermal response under the same NIR irradiation condition. As discussed above, BP@CSMA reached a higher temperature than BP@CSMA/CaCO₃, which might impose greater thermal stress on adherent cells during the early stage of culture. In contrast, the incorporation of CaCO₃ moderated the photothermal effect and simultaneously provided a more favorable mineral-associated microenvironment, which together supported by the enhanced cell proliferation in the BP@CSMA/CaCO₃.


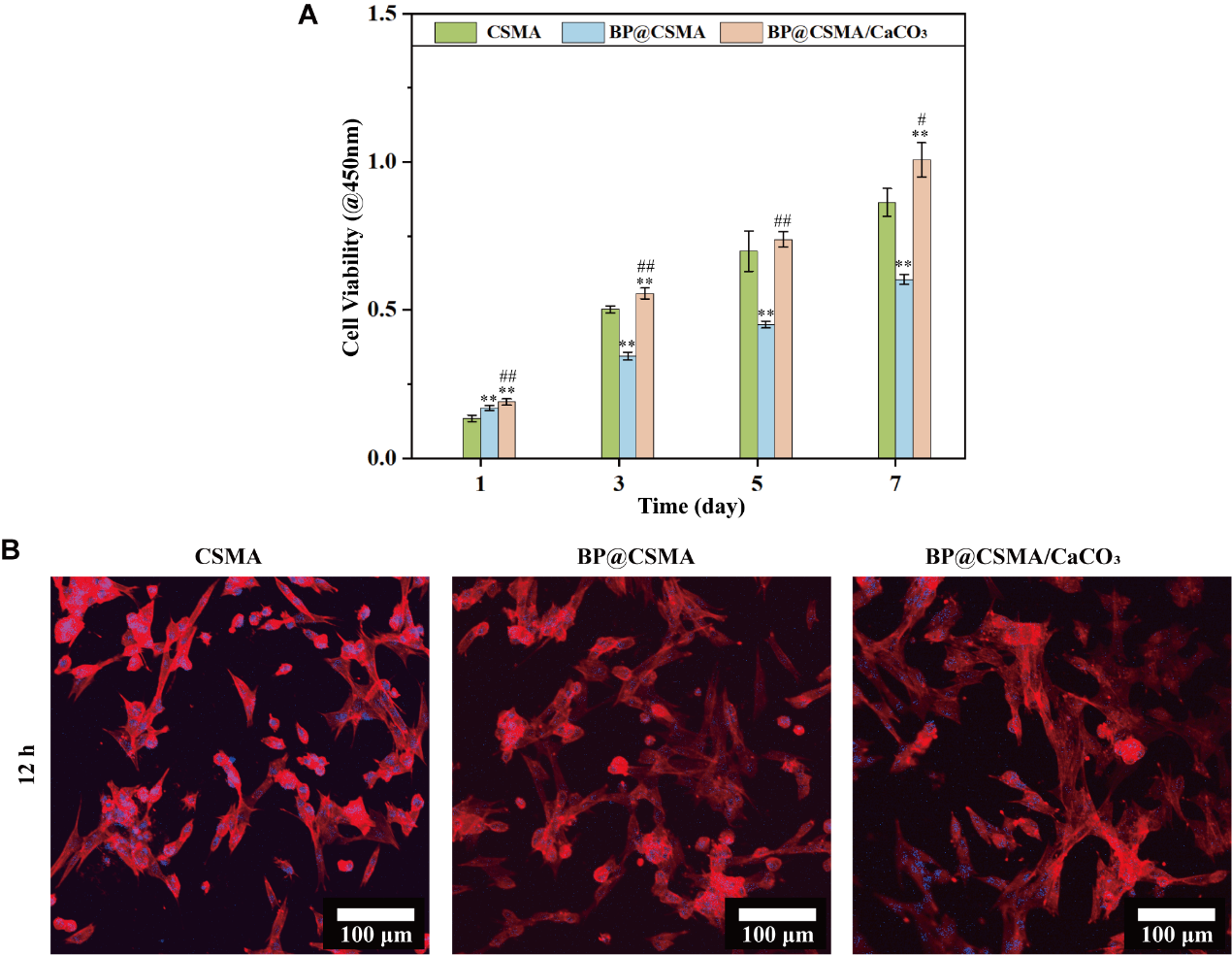


Figure 6. Proliferation and morphology of BMSCs cultured on composite hydrogels. (A) Quantitative analysis of BMSC proliferation on CSMA, BP@CSMA and BP@CSMA/CaCO₃ hydrogels for 7 days using CCK-8 assay. (B) Representative fluorescence images of BMSCs cultured on CSMA, BP@CSMA and BP@CSMA/CaCO₃ hydrogels for 12 h. F-actin was stained with rhodamine-phalloidin, and nuclei were stained with DAPI. Data are presented as mean ± SD, n = 3. ^*^*P* < 0.05 and ^**^*P* < 0.01 vs. CSMA; ^#^P < 0.05 and ^##^*P* < 0.01 vs. BP@CSMA.

3.5 Osteogenesis of the composite hydrogels

ALP, a secreted enzyme produced during early osteogenic differentiation of stem cells, plays a critical role in bone regeneration [71]. Semi-quantitative analysis of ALP activity in BMSCs cultured on all samples was presented in Figure 7B. On day 5, CSMA showed the lowest ALP activity, due to its lack of intrinsic osteoinductive properties. In contrast, BP@CSMA/CaCO₃ exhibited significantly higher ALP levels than all other groups. By day 7, the positive control demonstrated the most pronounced increase in ALP activity, reaching significantly higher levels when compared with BP@CSMA and CSMA. Similarly, BP@CSMA/CaCO₃ achieved nearly 1.3-fold higher levels than BP@CSMA. Additionally, the result of ALP staining (Figure 7A) was consistent with these semi-quantitative analyses, indicating both BP@CSMA and BP@CSMA/CaCO_3_ promoted ALP expression of BMSCs, suggesting their beneficial role in facilitating early osteogenic differentiation.

OPN, a secreted and phosphorylated glycoprotein, facilitates the deposition of mineralized extracellular matrix, which demonstrates a positive correlation with osteogenic activity [72]. As early as day 7, both BP@CSMA and BP@CSMA/CaCO₃ exhibited elevated OPN expression compared with CSMA. BP@CSMA/CaCO₃ reached its peak OPN level on day 7, reaching approximately 1.3-fold that of the positive control group (Figure 7C). By day 14, OPN expression in BP@CSMA and BP@CSMA/CaCO₃ had decreased slightly, whereas the positive control exhibited a continuous increase, and became significantly higher than the remaining groups. The decline in OPN levels by day 14 in BP@CSMA and BP@CSMA/CaCO₃ might be explained by its role in biomineralization. This temporal change might reflect the dynamic role of OPN during osteogenic differentiation. The early upregulation of OPN in BP-containing hydrogels suggested accelerated initiation of matrix mineralization, while the subsequent decrease might be associated with progression toward a more mature mineralized matrix.

Regarding OCN that is a marker of late-stage osteogenic differentiation secreted by mature osteoblasts [72], the positive control exhibited the highest level on day 7, and continued to increase until day 14. the BP@CSMA displayed similar OCN levels at two time points (Figure 7D). Whereas BP@CSMA/CaCO₃ group displayed a steady rise in OCN expression, reaching approximately twice the level of the BP@CSMA group by day 14. These results suggested that BP promoted BMSC osteogenic differentiation under mild NIR irradiation, which was further supported by ion release from the mineralized hydrogel. BP-mediated mild photothermal stimulation may activate thermal-response pathways and enhance osteogenic marker expression. In parallel, Ca²⁺ released from CaCO₃ and phosphorus from BP degradation provided complementary ionic cues for matrix mineralization and osteogenic regulation. Thus, BP@CSMA/CaCO₃ created a coordinated microenvironment that combined photothermal stimulation with Ca²⁺/Pi-related ionic support to promote bone regeneration.


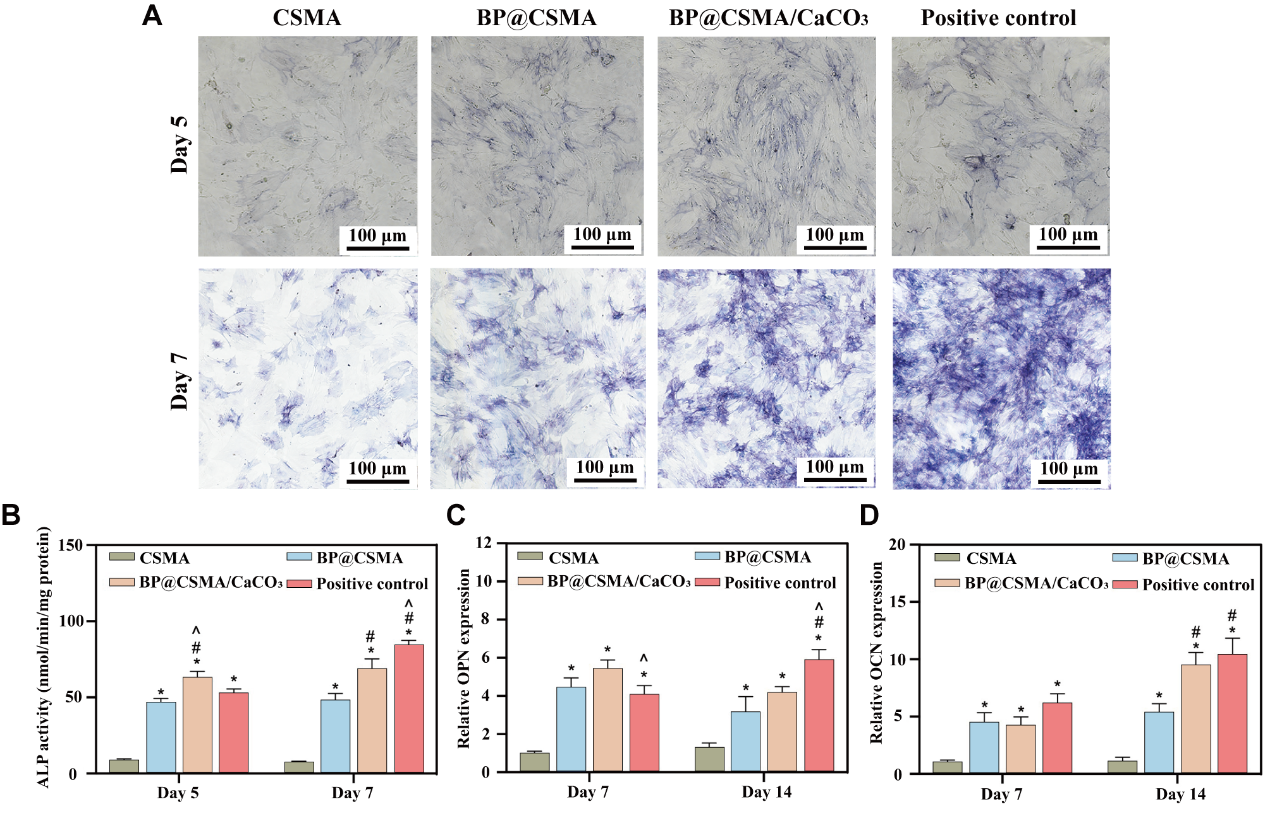


Figure 7. Osteogenic differentiation of BMSCs cultured on composite hydrogels. (A) Representative ALP staining images of BMSCs cultured on CSMA, BP@CSMA and BP@CSMA/CaCO₃ hydrogels. (B) Quantitative analysis of ALP activity normalized to total protein content. (C, D) Relative mRNA expression levels of OPN and OCN in BMSCs cultured on CSMA, BP@CSMA, and BP@CSMA/CaCO₃ hydrogels, determined by RT-qPCR. Data are presented as mean ± SD, n = 3. ^*^*P* < 0.05 vs. CSMA; ^#^*P* < 0.05 vs. BP@CSMA; ^^^*P* < 0.05 vs. BP@CSMA/CaCO₃.

It should be noted that all hydrogel groups were evaluated in a reduced-induction mineralizing medium without dexamethasone, whereas the positive control was cultured in complete osteogenic medium. This design was intended to minimize the dominant osteoinductive effect of Dex, thereby allowing a clear assessment of the intrinsic osteogenic contribution of the hydrogels. Since all hydrogel groups were cultured under the same medium conditions, the differences observed among CSMA, BP@CSMA, and BP@CSMA/CaCO₃ were mainly attributed to BP incorporation, CaCO₃ mineralization and their combined regulation of the cellular microenvironment.

Although BP-based materials and mineralized CSMA systems have both been reported previously [73,74], the present study was designed for infected bone defect repair, where antibacterial activity and osteogenic support were needed at different stages. In our system, BP served as the NIR-responsive component, while the introduction of CaCO₃ did more than provide mineral support. It also changed the physicochemical properties of the hydrogel, including its mechanical behavior, degradation behavior, phosphorus release profile and photothermal response. As a result, the role of CaCO₃ was not limited to simple composition addition, but extended to regulating the overall microenvironment and biological performance of the composite hydrogel.

3.6 Potential regulatory mechanism on osteogenesis effect of BP@CSMA/CaCO_3_

To elucidate the mechanism by which BP@CSMA/CaCO₃ enhanced osteogenic differentiation, RNA sequencing was performed to identify differentially expressed genes in BMSCs cultured on BP@CSMA/CaCO₃ compared with the CSMA (Figure 8A). As shown in Figure 8B, 175 genes were upregulated, and 93 genes were downregulated in the BP@CSMA/CaCO₃ group. Gene Ontology (GO) enrichment analysis revealed that the upregulated genes were primarily associated with immune response, extracellular matrix structural constitution, and cell migration (Figure 8C). Molecular functions critically involved in osteogenic regeneration, including structural molecule activity, extracellular matrix structural constituent binding, immunoglobulin receptor binding, and beta-tubulin binding, were significantly enriched (Figure 8E) [75]. Regarding to cellular components, BP@CSMA/CaCO₃ exhibited pronounced regulation of cytoskeletal elements, collagen trimers, and other key cellular structures (Figure 8D).


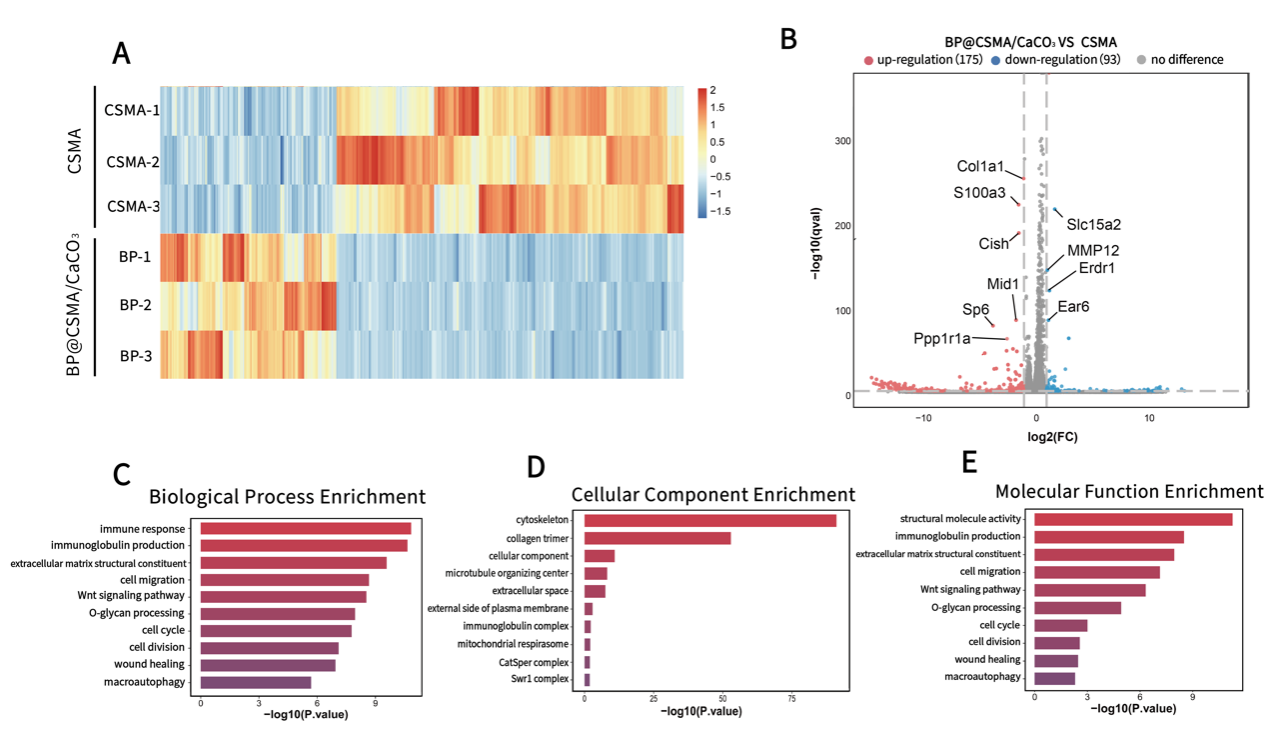


Figure 8. RNA sequencing analysis of BMSCs cultured on composite hydrogels. (A) Heatmap of differentially expressed genes between the CSMA and BP@CSMA/CaCO₃ groups. (B) Volcano plot of upregulated and downregulated genes in BP@CSMA/CaCO₃ compared with CSMA; (C-E) Gene Ontology (GO) enrichment analysis of differentially expressed genes, including biological process, cellular component and molecular function categories.

3.7 Bone formation ability of the composite hydrogels

The bone regeneration capacity was initially evaluated by micro-CT at 4- and 8- weeks post-implantation (Figure 9A). At 4 weeks, bone remodeling was primarily observed at the margins of the original defect across all groups. The control group displayed largely open defects with only 16.7% of the area filled by newly formed bone, and had little change by the 8-week time point. In contrast, both the BP@CSMA and BP@CSMA/CaCO₃ groups exhibited a time-dependent increase in new bone formation. Notably, the BP@CSMA/CaCO₃ group demonstrated the most substantial bone regeneration, with new bone extending from the defect margins toward the center and progressively filling the implantation site.

Both BP@CSMA and BP@CSMA/CaCO₃ exhibited significantly higher BV/TV values than the control group at both 4 and 8 weeks, with the highest value observed in the BP@CSMA/CaCO₃ (Figure 9B). The BS/BV value of BP@CSMA/CaCO₃ was also significantly higher than that of the other groups, especially at 4 weeks (Figure 9C), which might reflect abundant newly formed bone surfaces and active early remodeling within the defect region. In addition, Tb.Th in the BP@CSMA/CaCO₃ was significantly greater than that in the control group at both time points (Figure 9D). The further increases in BV/TV and Tb.Th at 8 weeks suggested progressive trabecular maturation and enhanced mineralized bone deposition. Together with the micro-CT reconstruction results, these findings supported the superior bone regeneration capacity of BP@CSMA/CaCO₃.


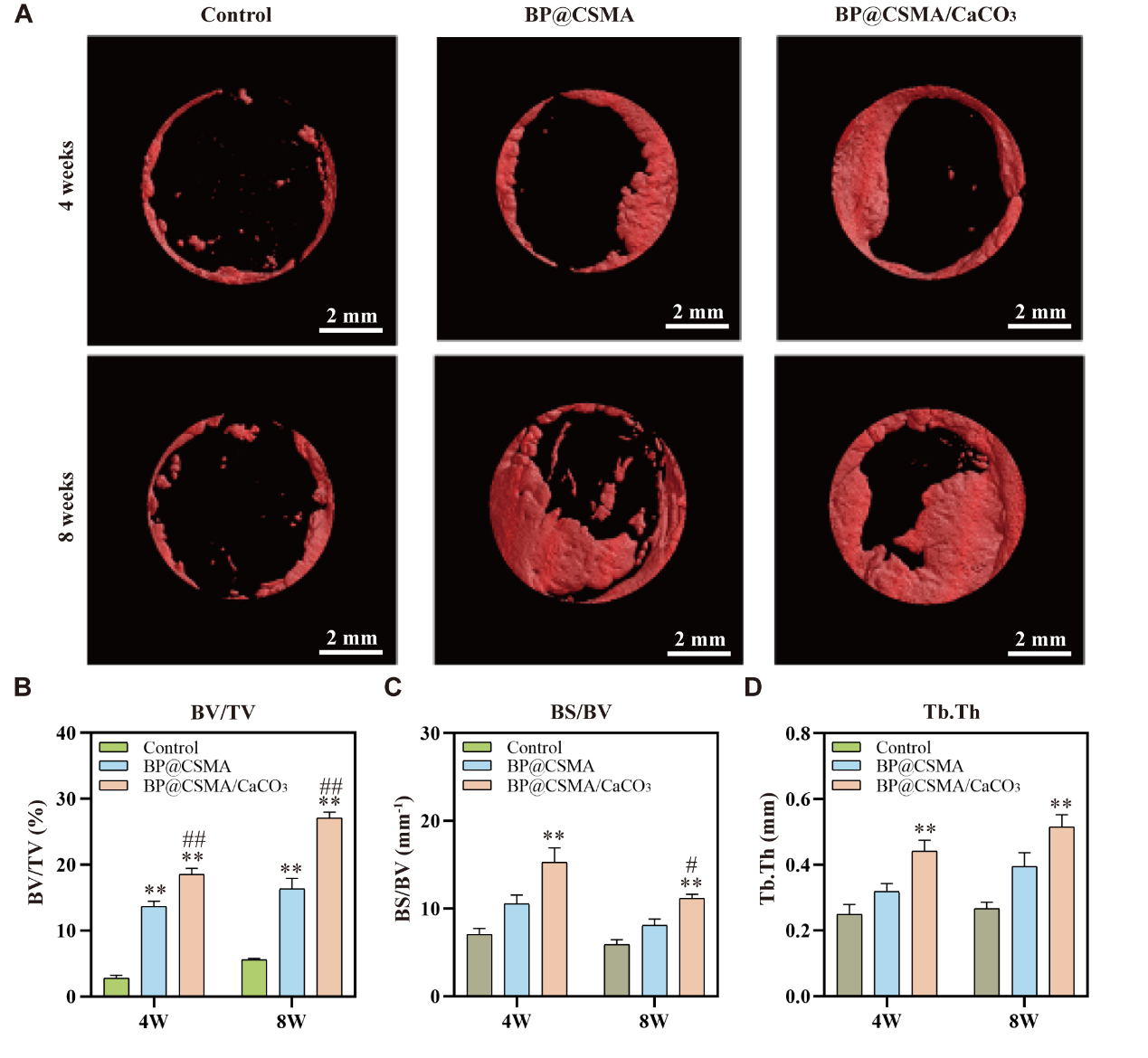


Figure 9. *In vivo* bone regeneration capacity of composite hydrogels by micro-CT. (A) Representative micro-CT images of calvarial defects in the control, BP@CSMA and BP@CSMA/CaCO₃ groups at 4 and 8 weeks after implantation. (B-D) Quantitative micro-CT analysis of bone volume fraction (BV/TV), bone surface density (BS/BV) and trabecular thickness (Tb.Th), respectively. Data are presented as mean ± SD, n = 6. ^*^*P* < 0.05 and ^**^*P* < 0.01 vs. control; ^#^*P* < 0.05 and ^##^*P* < 0.01 vs. BP@CSMA.

H&E staining showed no obvious inflammation, tissue necrosis, or rejection reaction in any group (Figure S5), indicating good in vivo biocompatibility. As illustrated in Figure 10A, newly formed bone was seldomly observed in control group, the defect area was mostly occupied by the fibrous tissue at 4 weeks. In contrast, BP@CSMA/CaCO₃ exhibited active osteogenesis at the host bone margins, with osteoid tissue distributed along the defect region. At 8 weeks, osteoid gradually transitioned into mineralized bone (green) in all groups. Specifically, greater bone thickness with higher degree of mineralization was observed in BP@CSMA/CaCO_3_ group, indicating its superior bone formation ability.

A similar osteogenic trend was observed using Masson staining (Figure 10B). At 4 weeks, only sparse and disorganized collagen fibers were observed in control and BP@CSMA groups, while BP@CSMA/CaCO₃ displayed more abundant collagen deposition and early mineralized bone formation. By 8 weeks, collagen fibers gradually became organized as time progressed. BP@CSMA/CaCO_3_ exhibited a highly ordered and layered arrangement of collagen fibers.


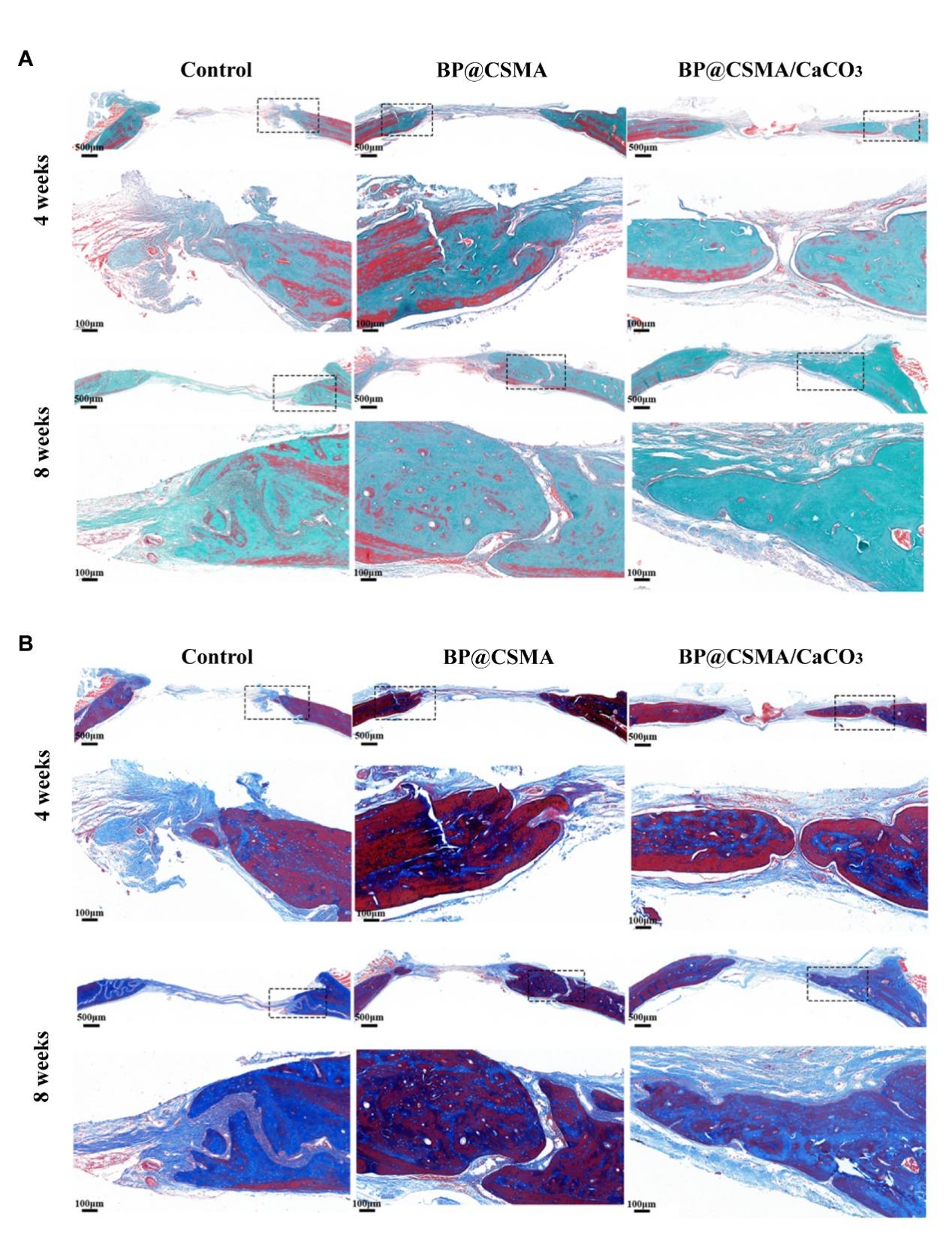


Figure 10. Histological evaluation of newly formed bone after composite hydrogel implantation. (A) Representative Goldner’s trichrome staining images of calvarial defects in the control, BP@CSMA, and BP@CSMA/CaCO₃ groups at 4 and 8 weeks (osteoid tissue is shown in orange/red, and mature mineralized bone is shown in green). (B) Representative Masson’s trichrome staining images of calvarial defects in the control, BP@CSMA and BP@CSMA/CaCO₃ groups at 4 and 8 weeks (collagen fibers are shown in blue, while soft tissues or cytoplasm are shown in orange/red). n = 6.

Nevertheless, several limitations should be acknowledged. The mechanistic investigation in this study remained preliminary, which required further in-depth pathway verification in our future work. Additionally, although BP@CSMA/CaCO₃ exhibited superior bone regeneration compared with CSMA and BP@CSMA, the respective *in vivo* contributions of CaCO₃ mineralization and BP incorporation could not be fully resolved since the CSMA/CaCO₃-only group was not included. In addition, the agar-based trans-tissue model was only a simplified proof-of-concept system *in vitro*, which could not reproduce the multilayer structure vascular perfusion, or heterogeneous properties of real tissues.

Despite these limitations, the present findings indicated the BP@CSMA/CaCO₃ hydrogel might represent a promising candidate for the treatment of bone defects complicated by infection, where simultaneous antibacterial activity and osteogenic support were both required. Its injectable and photo-responsive properties also facilitated localized administration and on-demand postoperative modulation.

**4. Conclusion**

In this study, BP@CSMA/CaCO₃ was developed as a NIR-responsive multifunctional hydrogel for infected bone defect repair. The incorporation of BP and *in situ*-mineralized CaCO₃ improved hydrogel hydrophilicity, mechanical strength, degradation stability, and sustained Ca²⁺/Pi release. Under NIR irradiation, the composite hydrogel exhibited strong antibacterial activity against *S. aureus* and *E. coli*, mainly through photothermal heating and ROS generation*.* Meanwhile, BP@CSMA/CaCO₃ promoted BMSC adhesion, proliferation and osteogenic differentiation by increased ALP activity, and upregulated OPN and OCN. RNA sequencing analysis revealed the involvement of cytoskeletal organization and immune-related pathways in the enhanced osteogenic process. The calvarial defect model confirmed its superior *in vivo* bone regeneration ability. These findings indicated that BP@CSMA/CaCO₃ provided a promising platform combining antibacterial protection and osteogenic support for infected bone defect repair.

**Funding**

This work was supported by National Natural Science Foundation of China (No. 82372135).

**Conflict of interest statement**

The author(s) declared no potential conflicts of interest with respect to the research, authorship, and/or publication of this article.

**References**

[1] Chandran M, Amour KB, Fujiwara S, Ha Y-C, Tang H, Hwang J-S, Tinker J, Eisman JA. Prevalence of osteoporosis and incidence of related fractures in developed economies in the Asia Pacific region: a systematic review. Osteoporosis International **2023**;34:1037-1053.

[2] Ferraz MP. Bone grafts in dental medicine: An overview of autografts, allografts and synthetic materials. Materials **2023**;16(11):4117.

[3] Feng P, Liu LX, Yang F, Min R, Wu P, Shuai CJ. Shape/properties collaborative intelligent manufacturing of artificial bone scaffold: structural design and additive manufacturing process. Biofabrication **2024**;17(1):012005.

[4] Cui Z-K, Kim S, J. Baljon J, M. Wu B, Aghaloo T, Lee M. Microporous methacrylated glycol chitosan-montmorillonite nanocomposite hydrogel for bone tissue engineering. Nature Communications **2019**;10:3523.

[5] Xue X, Hu Y, Deng YH, Su JC. Recent advances in design of functional biocompatible hydrogels for bone tissue engineering. Advanced Functional Materials **2021**;31(19):2009432.

[6] Mohammadi Z, Eini M, Rastegari A, Tehrani MR. Chitosan as a machine for biomolecule delivery: A review. Carbohydrate Polymers **2021**;256:117414.

[7] Zhou C-E, Kan C-W, Sun C, Du JM, Xu CH. A review of chitosan textile applications. AATCC Journal of Research **2019**;6:8-14.

[8] Sharkawy A, Barreiro MF, Rodrigues AE. Chitosan-based pickering emulsions and their applications: A review. Carbohydr Polym **2020**;250:116885.

[9] Pal P, Pal A, Nakashima K, Yadav BK. Applications of chitosan in environmental remediation: A review. Chemosphere **2021**;266:128934.

[10] Deng DF, Peng DY, Lv JH, Zhang WC, Tian HQ, Wang TQ, Wu M, Zhao Y. Double-network hydrogel based on methacrylated chitosan/hyaluronic acid coacervate for enhanced wet-tissue adhesion. Biomacromolecules **2025**;26(4):2317-2330.

[11] Feng PP, He CN, Li GR, Li J, Luo Y, Chen YQ, Tang Y, Ma JY, Ke CH. Light-cured injectable composite hydrogel based on chitosan and decellularized matrix modulates stem cell aggregation behavior for accelerating cartilage defect repair. International Journal of Biological Macromolecules **2025**;295:139711.

[12] Kim HD, Hong XC, An Y-H, Park MJ, Kim D-G, Greene AK, Padwa BL, Hwang NS, Lin R-Z, Melero-Martin JM. A biphasic osteovascular biomimetic scaffold for rapid and self-sustained endochondral ossification. Advanced Healthcare Materials **2021**;10(13):2100070.

[13] Yang JZ, Liu F, Zhou CS, Li HJ, Yang GL, Fang SY, Lee I-S, Liu Y, Bai H, Chen C. 3D printed porous titanium filled with mineralized UV-responsive chitosan hydrogel promotes cell proliferation and osteogenesis *in vitro.* Journal of Materials Science & Technology **2023**;142:34-44.

[14] Wang K, Jiang K, Luo C, You XH, Shen H-Y, Wu DW, Huang F-W, Li LL, Huang SS, Xu J-Z, Li Z-M. An osteoimmunomodulatory Ca^2+^/Zn^2+^-doped scaffold promotes M2 macrophage polarization via the src-mediated chemoking signaling pathway to enhance osteoinduction. Composites Part B: Engineering **2024**;284:111653.

[15] Jeyaraman M, Jeyaraman N, Konkathi VK, Nallakumarasamy A, Muthu S, Khanna M. Bacteriophage therapy in implant-related orthopedic infections. Indian Journal of Orthopaedics **2022**;56:1685-1693.

[16] Ojeda-Thies C, Rojo-Carpintero A, Soria-Perdomo F, Ramos-Pascua LR. Implant-associated infection after hip fracture surgery in elderly patients: Risk factors and mortality. Injury **2024**;55:111756.

[17] Gristina AG, Info A, Affiliations. Biomaterial-centered infection: Microbial adhesion versus tissue integration. Science **1987**;237(4822):1588-1595.

[18] Arciola CR, Campoccia D, Montanaro L. Implant infections: adhesion, biofilm formation and immune evasion. Nature Reviews Microbiology **2018**;16:397-409.

[19] Xu LJ, Gao S, Zhou RB, Zhou F, Qiao Y, Qiu D. Bioactive pore-forming bone adhesives facilitating cell ingrowth for fracture healing. Advanced Materials **2020**;32(10):1907491.

[20] Niu X, Xiao S, Li K, He W, Huang D, Yu H, Li X. Nanofiber‐reinforced self‐adaptive hydrogels with desired sequential delivery capability of bioactive factors and magnesium for vascularized osteochondral regeneration. Advanced Functional Materials **2025**;35(47):2503948.

[21] Niu X, Xiao S, Huang R, Huang D, Aifantis K E, Yu H, Li X. ZIF-8-modified hydrogel sequentially delivers angiogenic and osteogenic growth factors to accelerate vascularized bone regeneration. Journal of Controlled Release **2024**;374: 154-170.

[22] Wang B, Xiao S, Liao J, Huang Y, Guan X, Wang C, Li X. Directional biomimetic scaffold-mediated cell migration and pathological microenvironment regulation accelerate diabetic bone defect repair. ACS nano **2025**;19(36):32382-32404.

[23] Hanlon D, Backes C, Doherty E, Cucinotta CS, Berner NC, Boland C, Lee K, Harvey A, Lynch P, Gholamvand Z, Zhang SF, Wang KP, Moynihan G, Pokle A, Ramasse QM, McEvoy N, Blau WJ, Wang J, Abellan G, Hauke F, Hirsch A, Sanvito S, ORegan DD, Duesberg GS, Nicolosi V, Coleman JN. Liquid exfoliation of solvent-stabilized few-layer black phosphorus for applications beyond electronics. Nature Communications **2015**;6:9563.

[24] Ge XX, Xia ZH, Guo SJ. Recent advances on black phosphorus for biomedicine and biosensing. Advanced Functional Materials **2019**;29(29):1900318.

[25] Long J, Yao ZY, Zhang W, Liu B, Chen KM, Li L, Teng B, Du X-F, Li CR, Yu X-F, Qin L, Lai YX. Regulation of osteoimmune microenvironment and osteogenesis by 3D-printed PLAG/black phosphorus scaffolds for bone regeneration. Advanced Science **2023**;10(28):2302539.

[26] Qing Y, Li R, Li S, Li Y, Wang X, Qin Y. Advanced black phosphorus nanomaterials for bone regeneration. International Journal of Nanomedicine **2020**;15:2045-2058.

[27] Shao JD, Ruan CS, Xie HH, Li ZB, Wang HY, Chu PK, Yu X-F. Black-phosphorus-incorporated hydrogel as a sprayable and biodegradable photothermal platform for postsurgical treatment of cancer. Advanced Science **2018**;5:1700848.

[28] Wang F, Wu QH, Jia GP, Kong LC, Zuo RT, Feng K, Hou MF, Chai YM, Xu J, Zhang CF, Kang QL. Black phosphorus/MnO_2_ nanocomposite disrupting bacterial thermotolerance for efficient mild-temperature photothermal therapy. Advanced Science **2023**;10(30):2303911.

[29] Huang KQ, Wu J, Gu ZP. Black phosphorus hydrogel scaffolds enhance bone regeneration via a sustained supply of calcium-free phosphorus. ACS Applied Materials & Interfaces **2019**;11(3):2908-2916.

[30] Bai XS, Wang RX, Hu XH, Dai Q, Guo JX, Cao TY, Du WL, Cheng YN, Xia SX, Wang DD, Yang LY, Teng L, Chen DF, Liu YJ. Two-dimensional biodegradable black phosphorus nanosheets promote large full-thickness wound healing through in situ regeneration therapy. ACS NANO **2024**;18(4):3553-3574.

[31] Kuriakose S, Ahmed T, Balendhran S, Bansal V, Sriram S, Bhaskaran M, Walia S. Black phosphorus: ambient degradation and strategies for protection. 2D Materials **2018**;5:032001.

[32] Cao WY, Lai DW, Yang J, Liu L, Wu H, Wang J, Liu YJ. Research progress on the preparation methods for and flame retardant mechanism of black phosphorus and black phosphorus nanosheets. Nanomaterials **2024**;14(10):892.

[33] Ma PF, Da JL, Zhao GH, Suo FY, Li Y, Zhou XC, Li Y, Han YH, Zou MY, Dou XM. Injectable light-responsive hydrogel dressing promotes diabetic wound healing by enhancing wound angiogenesis and inhibiting inflammation. Polymers **2025**;17(5):607.

[34] Qiu ML, Tulufu N, Tang GQ, Ye WK, Qi J, Deng LF, Li CW. Black Phosphorus Accelerates Bone Regeneration Based on Immunoregulation. Advanced Science (Weinh) **2024**;11(1):2304824.

[35] Yang J, Liu F, Zhou C, Li H, Yang G, Fang S, Lee IS, Liu Y, Bai H, Chen C. 3D printed porous titanium filled with mineralized UV-responsive chitosan hydrogel promotes cell proliferation and osteogenesis in vitro. Journal of Materials Science & Technology **2023**;142:34-44.

[36] Banivaheb S, Dan S, Hashemipour H, Kalantari M. Maryam Synthesis of modified chitosan TiO2 and SiO2 hydrogel nanocomposites for cadmium removal. Journalof Saudi Chemical Society **2021**;25(8):101283.

[37] Bento de Carvalho T, Barbosa JB, Teixeira P. Assessing antimicrobial efficacy on plastics and other non-porous surfaces: A closer look at studies using the ISO 22196:2011 standard. Biology **2024**;13(1):59.

[38] Jung O, Smeets R, Hartjen P, Schnettler R, Feyerabend F, Klein M, Wegner N, Walther F, Stangier D, Henningsen A, Rendenbach C, Heiland M, Barbeck M, Kopp A. Improved *in vitro* test procedure for full assessment of the cytocompatibility of degradable magnesium based on ISO 10993-5/-12. International Journal of Molecular Sciences **2019**;20(2):255.

[39] Wu Y, Liao Q, Wu L, Luo Y, Zhang W, Guan M, Wang H. ZnL2-BPs integrated bone scaffold under sequential photothermal mediation: a win–win strategy delivering antibacterial therapy and fostering osteogenesis thereafter. ACS nano **2021**;15(11):17854-17869.

[40] Zhang BW, Sun ZJ, Chen WJ, Li Q, Yan QF. Environmental stability diversity in elemental phosphorus: The case of black, violet, and fibrous red phosphorus. Small **2024**;21(37):2505083.

[41] Huang SY, Zhang GW, Fan FR, Song CY, Wang FJ, Xing QX, Wang C, Wu H, Yan HG. Strain-tunable van der Waals interactions in few-layer black phosphorus. Nature Communications **2019**;10(1):2447.

[42] Lei DM, Xiao YC, Xi M, Jiang Y, Li Y. Thermochromic and conductive hydrogels with tunable temperature sensitivity for dual sensing of temperature and human motion. Journal of Materials Chemistry C **2024**;12:232-244.

[43] Dai PP, Ge X, Sun CX, Jiang H, Zuo WC, Wu PC, Liu C, Deng SY, Yang J, Dai JJ, Ju YM. A novel methacryloyl chitosan hydrogel microneedles patch with sustainable drug release property for effective treatment of psoriasis. Macromolecular Bioscience **2023**;23(12):2300194.

[44] Zhang YW, Li ZX, Wang ZQ, Yan BM, Shi A, Xu J, Guan JZ, Zhang L ,Zhou PH, Mao YJ. Mechanically enhanced composite hydrogel scaffold for *in situ* bone repairs. Biomaterials Advances **2022**;134:112700.

[45] Cao JY, He P, Brent JR, Yilmaz H, Lewis DJ, Kinloch IA, Derbye B. Supercapacitor electrodes from the *in situ* reaction between two-dimensional sheets of black phosphorus and graphene oxide. ACS Applied Materials & Interfaces **2018**;10(12):10330-10338.

[46] Du HC, Yuan TY, Zhao R, Hirsch M, Kessler M, Amstad E. Reinforcing hydrogels with *in situ* formed amorphous CaCO_3_. Biomaterials Science **2022**;10(17):4949-4958.

[47] Ding QY, Sun TF, Su WJ, Jing XR, Ye B, Su YL, Zeng L, Qu YZ, Yang X, Wu YZ, Luo ZQ, Guo XD. Bioinspired multifunctional black phosphorus hydrogel with antibacterial and antioxidant properties: A stepwise countermeasure for diabetic skin wound healing. Advanced Healthcare Materials **2022**;11(12):2102791.

[48] Li DK, Zhan W, Zuo W, Li LP, Zhang J, Cai GY, Tian Y. Elastic, tough and switchable swelling hydrogels with high entanglements and low crosslinks for water remediation. Chemical Engineering Journal **2022**;450(4):138417.

[49] Foudazi R, Zowada R, Manas-Zloczower I, Feke DL. Porous hydrogels: Present challenges and future opportunities. Langmuir **2023**;39(6):2092-2111.

[50] Mollajavadi MY, Tarigheh FF, Eslami-Farsani R. Self-healing polymers containing nanomaterials for biomedical engineering applications: A review. Polymer Composites **2023**;44(10):6869-6889.

[51] Lv XX, Huang Y, Hu MT, Wang YX, Wang HX, Chen H, Ma L, Zhang YH, Dai HJ. Biopolymer-based hydrogel beads with enhanced stability and pH/ion-responsive controlled release through a dual-cross-linking strategy. Biomacromolecules **2025**;26(10):6458-6474.

[52] Baek J, Fan Y, Jeong SH, Lee HY, Jung HD, Kim HE, Kim S, Jang TS. Facile strategy involving low-temperature chemical cross-linking to enhance the physical and biological properties of hyaluronic acid hydrogel. Carbohydrate Polymers **2018**;202:545-553.

[53] Miao Y, Chen Y, Luo J, Liu X, Yang Q, Shi X, Wang Y. Black phosphorus nanosheets-enabled DNA hydrogel integrating 3D-printed scaffold for promoting vascularized bone regeneration. Bioactive Materials **2023**;21:97-109.

[54] Dong M, Sun Y, Dunstan DJ, Young RJ, Papageorgiou DG. Mechanical reinforcement from two-dimensional nanofillers: model, bulk and hybrid polymer nanocomposites. Nanoscale **2024**;16:13019-13055.

[55] Du H, Yuan T, Zhao R, Hirsch M, Kessler M, Amstad E. Reinforcing hydrogels with in situ formed amorphous CaCO₃. Biomaterials Science **2022**;10(17):4949-4958.

[56] Miao Y, Chen Y, Luo J, Liu X, Yang Q, Shi X, Wang Y. Black phosphorus nanosheets-enabled DNA hydrogel integrating 3D-printed scaffold for promoting vascularized bone regeneration. Bioactive Materials **2023**; 21:97-109.

[57] Zeng X, Zhu Z, Chang W, Wu B, Huang W. Biomineralization process inspired in situ growth of calcium carbonate nanocrystals in chitosan hydrogels. Applied Sciences **2024**;14(20):9193.

[58] Xu DY, Liu J, Wang YX, Jian YY, Wu WW, Lv RC. Black phosphorus nanosheet with high thermal conversion efficiency for photodynamic/photothermal/immunotherapy. ACS Biomaterials Science & Engineering **2020**;6(9):4940-4948.

[59] Tan SJ, Yuan XN, Song ZY, Lin ZY, Zhao F, Wang L, Gao J. A sequential treatment strategy by copper ion-doped nanofiber dressing for highly efficient biofilm combating and rapid wound healing. Chemical Engineering Journal **2024**;500:156947.

[60] Menikou G, Damianou C. Acoustic and thermal characterization of agar based phantoms used for evaluating focused ultrasound exposures. Journal of Therapeutic Ultrasound **2017**;5:14.

[61] Wu X, Yang F, Cai S, Pu KY, Hong GS. Nanotransducer-enabled deep-brain neuromodulation with NIR-II light. ACS Nano **2023**;17(9):7941-7952.

[62] Fang J, Wan Y, Sun Y, Sun XL, Qi ML, Cheng S, Li CY, Zhou YM, Xu L, Dong B, Wang L. Near-infrared-activated nanohybrid coating with black phosphorus/zinc oxide for efficient biofilm eradication against implant-associated infections. Chemical Engineering Journal **2022**;435:134935.

[63] Du WX, Chen WJ, Wang J, Cheng L, Wang JW, Zhang HJ, Song L, Hu Y, Ma XP. Combined photothermal and sonodynamic therapy using a 2D black phosphorus nanosheets loaded coating for efficient bacterial inhibition and bone-implant integration. Biomaterials Advances **2022**;136:212794.

[64] Zhao P, Zhang Y, Chen XA, Xu C, Guo JZ, Deng MG, Qu XW, Huang PS, Feng ZJ, Zhang JM. Versatile hydrogel dressing with skin adaptiveness and mild photothermal antibacterial activity for methicillin-resistant staphylococcus aureus-infected dynamic wound healing. Advanced Science **2023**;10(11):2206585.

[65] Huang S, Xu S, Hu Y, Zhao X, Chang L, Chen Z, Mei X. Preparation of NIR-responsive, ROS-generating and antibacterial black phosphorus quantum dots for promoting the MRSA-infected wound healing in diabetic rats. Acta Biomaterialia **2022**;137:199-217.

[66] Wang F, Wu QH, Jia GP, Kong L, Zuo R, Feng K, Hou MF, Kang Q. Black phosphorus/MnO2 nanocomposite disrupting bacterial thermotolerance for efficient mild‐temperature photothermal therapy. Advanced Science **2023**;10(30):2303911.

[67] Miller F, Hinze U, Chichkov B, Leibold W, Lenarz T, Paasche G. Validation of eGFP fluorescence intensity for testing in vitro cytotoxicity according to ISO 10993-5. Journal of Biomedical Materials Research Part B: Applied Biomaterials **2015**;105(4):701-925.

[68] Younis MR, He G, Qu JL, Lin J, Huang P, Xia XH. Inorganic nanomaterials with intrinsic singlet oxygen generation for photodynamic therapy. Advanced Science **2021**;8(21):2102587.

[69] Cheng H, Chen J, Wang Y, Zhang Y, Qin T, Sun H, Liu J. Sr-doped surfaces with2D black phosphorus nanosheets for enhanced photothermal antibacterial activity and zirconia implant osseointegration. Regenerative Biomaterials **2025**;12:rbaf033.

[70] Na P, Jiang JL, Lv RP, Yang F, Li SF, Chen XZ. Advances in mild photothermal hydrogel-based therapies for bone and soft tissue injuries. Frontiers in Cell and Developmental Biology **2025**;13:1696209.

[71] Ansari S, Ito K, Hofmann S. Alkaline phosphatase activity of serum affects osteogenic differentiation cultures. ACS Omega **2022**;7(15):12724-12733.

[72] Lin X, Patil S, Gao YG, Qian A. The bone extracellular matrix in bone formation and regeneration. Frontiers in Pharmacology **2020**;11:757.

[73] Long J, Yao Z, Zhang W, Liu B, Chen K, Li L, Lai Y. Regulation of osteoimmune microenvironment and osteogenesis by 3D‐printed PLAG/black phosphorus scaffolds for bone regeneration. Advanced Science **2023**;10(28):2302539.

[74] Samani S, Nazbar A, Vasei M, Bonakdar S, Azami M. A cell-laden methacrylated chitosan-based photocrosslinkable hydrogel for bone tissue engineering and its in vitro structural and biological characterization. Scientific Reports **2026**.

[75] Zhou L, Fan M, Xia L, Chen Y, Han X, Fang B. B10 cell-induced PD-L1/PD-1-linked macrophage polarization in periodontitis. Journal of Translational Internal Medicine **2025**;13(4):338-348.
