# Supplementary material for: Black phosphorus-loaded mineralized UV-responsive chitosan hydrogel for enhanced osteogenesis and antibacterial activity
Source: Regen Biomater. 2026 Jun 17;13:rbag128. doi: 10.1093/rb/rbag128 (PMC13363252; doi:10.1093/rb/rbag128)
Supplement: rbag128_Supplementary_Data [file rbag128_supplementary_data.zip › Supplement-information-proof(revised).docx]

Supplement information

for

**Black phosphorus-loaded mineralized UV-responsive chitosan hydrogel for enhanced osteogenesis and antibacterial activity**


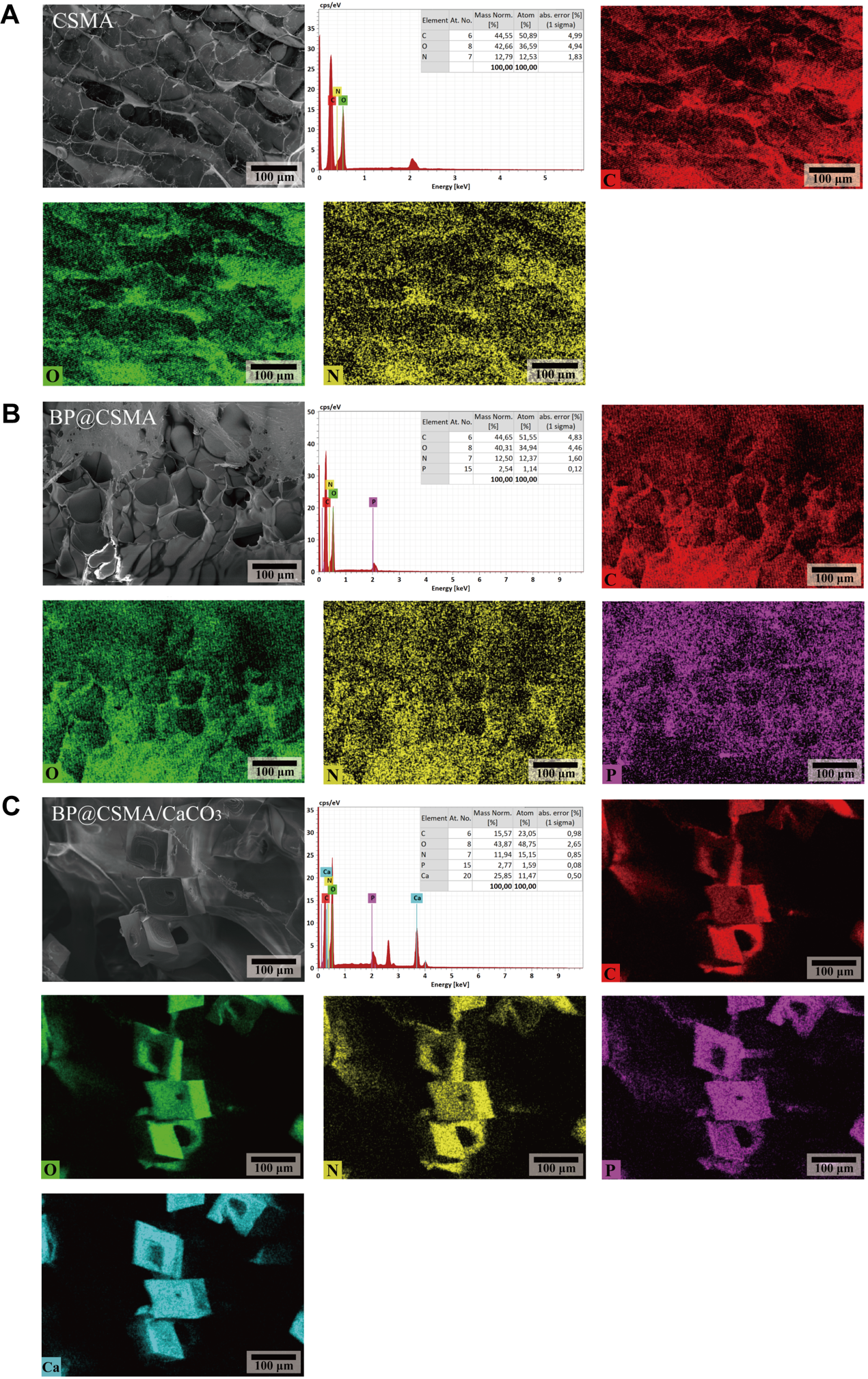


Figure S1. EDS elemental mapping of CSMA, BP@CSMA and BP@CSMA/CaCO_3_ hydrogels.


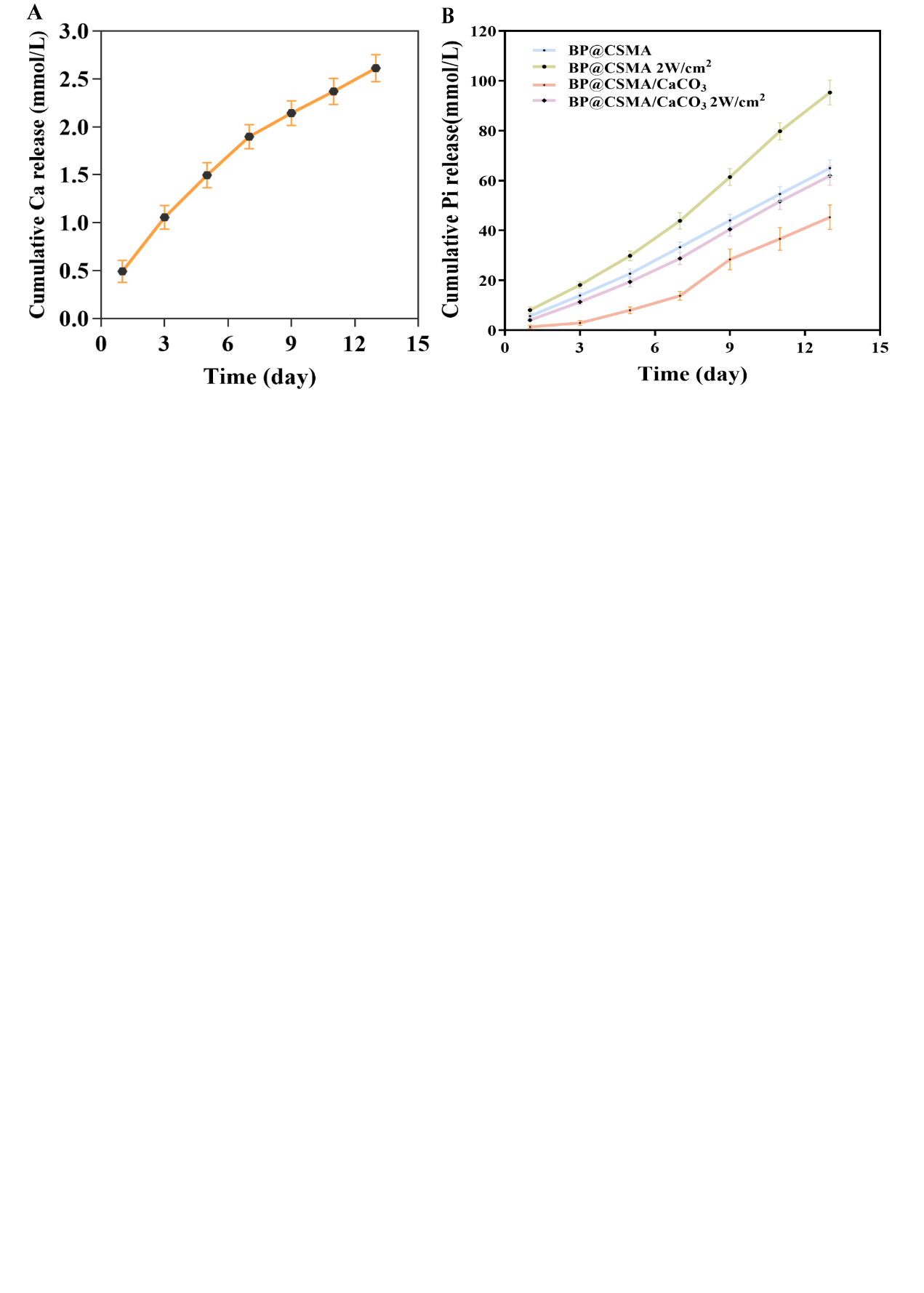


Figure S2. Ion release profiles of composite hydrogels. (A) Cumulative Ca²⁺ release from BP@CSMA/CaCO₃. (B) Cumulative inorganic phosphate (Pi) release from BP@CSMA and BP@CSMA/CaCO₃ hydrogels with or without NIR irradiation. Data are presented as mean ± SD, n = 3.


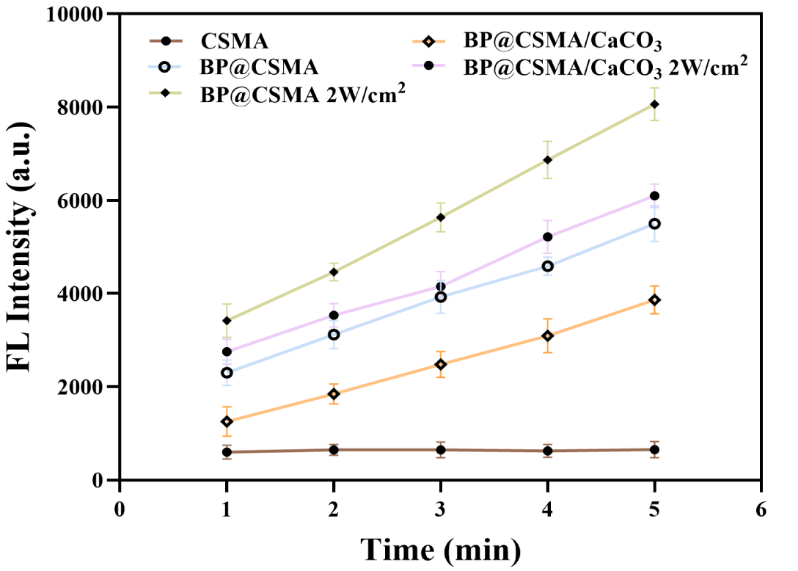


Figure S3. Singlet oxygen generation of composite hydrogels. Data are presented as mean ± SD, n = 3.


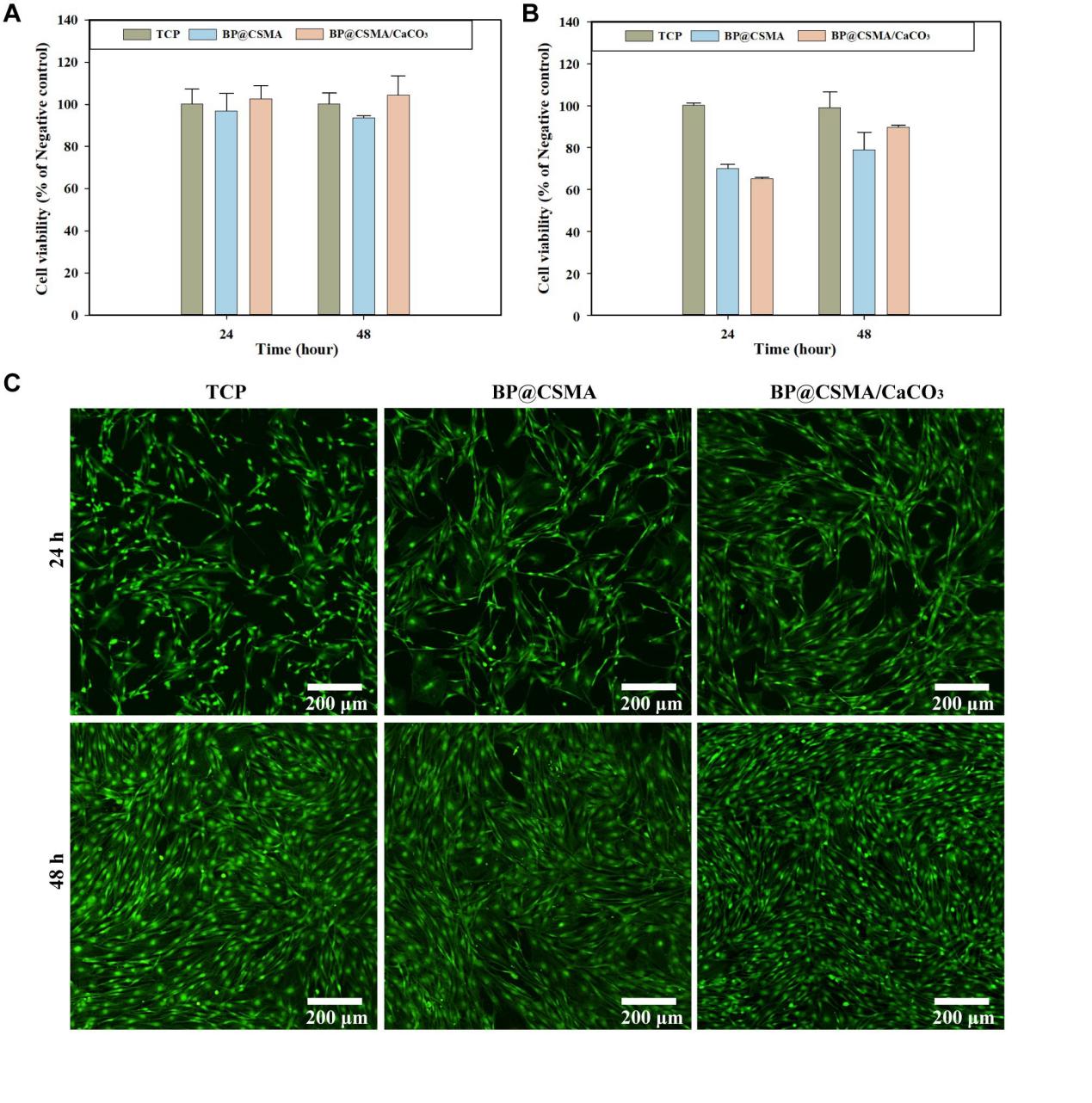


Figure S4. Cytocompatibility of BP@CSMA and BP@CSMA/CaCO₃ hydrogels. (A) Cell viability of BMSCs cultured with extracts from BP@CSMA and BP@CSMA/CaCO₃ hydrogels for 24 and 48 h by CCK-8 assay. (B) Relative cell viability of BMSCs cultured on BP@CSMA and BP@CSMA/CaCO₃ hydrogels after 808 nm NIR irradiation at 1.0 W/cm² for 1 min (Cell viability referred to the ratio of the OD value in irradiated samples to that in non-irradiated controls). (C) Representative live/dead staining images of BMSCs after 24 and 48 h of culture by Calcein-AM/PI staining. Data are presented as mean ± SD, n = 3.


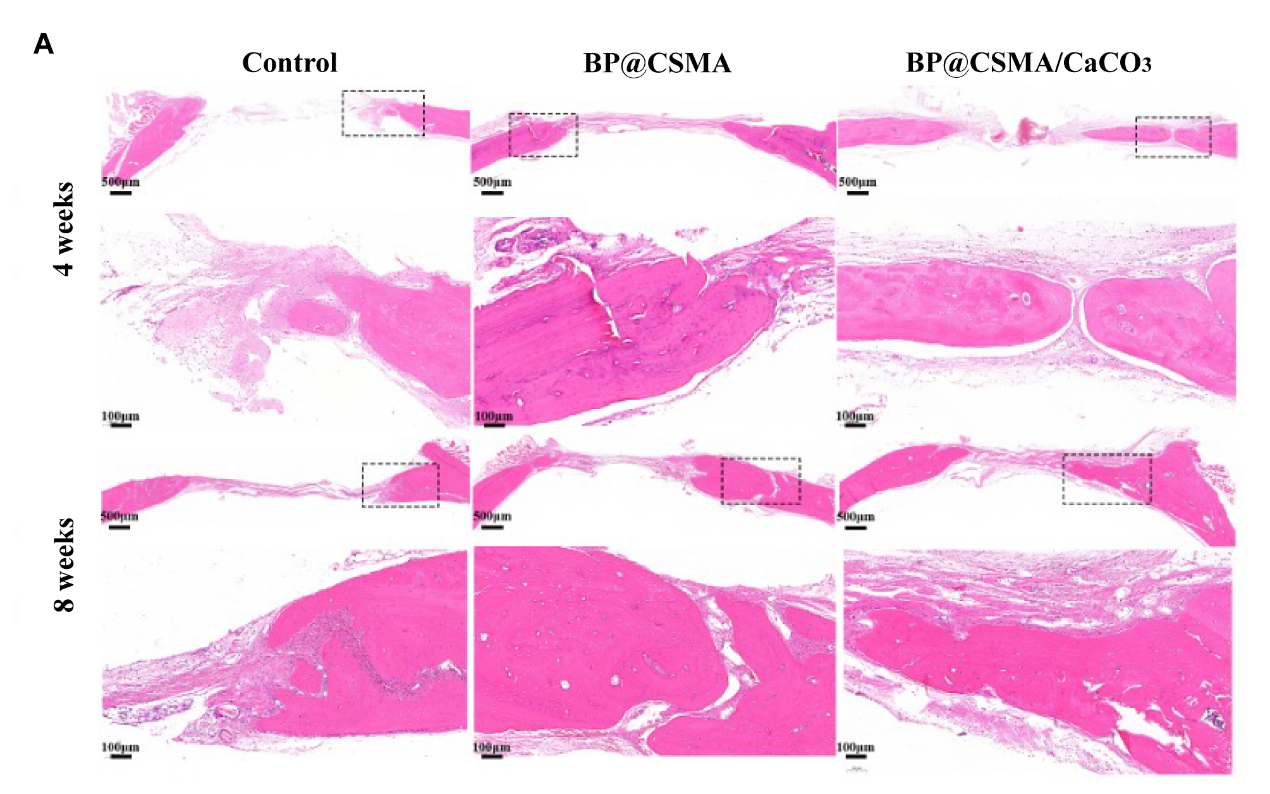


Figure S5. H&E staining of newly formed bone after composite hydrogel implantation at 4 and 8 weeks. n = 6.

Table S1. NIR irradiation parameters in different experiments.

| Experiment | Purpose | Power density | Duration | Frequency |
| --- | --- | --- | --- | --- |
| Photothermal performance | Evaluate temperature increase/photothermal stability | 1 or 2 W/cm² | 5 min | Single exposure; 3 on/off cycles for stability test |
| Agar-block simulation | Assess trans-tissue photothermal response | 2 W/cm² | 5 min | Single exposure |
| Antibacterial test | Antibacterial photothermal treatment | 2 W/cm² | 5 min | Single exposure |
| Cytocompatibility | Evaluate effect of mild NIR on BMSC viability | 1 W/cm² | 1 min | Single exposure |
| Cell proliferation | Mild photothermal stimulation | 1 W/cm² | 1 min | Once every 3 days |
| Osteogenic differentiation | Mild photothermal stimulation | 1 W/cm² | 1 min | Once every 3 days |
| Animal experiment | Post-implantation photothermal stimulation | 1 W/cm² | 3 min | Once every 3 days for 2 weeks |

Table S2. Primers for qRT-PCR

| Genes | Forward primer | Reverse primer |
| --- | --- | --- |
| OCN | CTCACTCTGCTGGCCCTGAC | CACCTTACTGCCCTCCTGCTTG |
| OPN | CAAGGTCATCCCAGTTGCCCAG | GCTTTGGAACTCGCCTGACTGTC |
| GAPDH | GAAGGTCGGTGTGAACGGATTTG | CATGTAGACCATGTAGTTGAGGTCA |
